# White matter alterations in pediatric brainstem glioma: An national brain tumor registry of China study

**DOI:** 10.3389/fnins.2022.986873

**Published:** 2022-09-09

**Authors:** Peng Zhang, Guocan Gu, Yunyun Duan, Zhizheng Zhuo, Changcun Pan, Pengcheng Zuo, Yi Wang, Xiaoou Li, Zhuang Jiang, Liying Qu, Yaou Liu, Liwei Zhang

**Affiliations:** ^1^Department of Neurosurgery, Beijing Tiantan Hospital, Capital Medical University, Beijing, China; ^2^Department of Radiology, Beijing Tiantan Hospital, Capital Medical University, Beijing, China; ^3^China National Clinical Research Center for Neurological Diseases, Beijing, China; ^4^Beijing Neurosurgical Institute, Capital Medical University, Beijing, China; ^5^Beijing Key Laboratory of Brain Tumor, Beijing, China

**Keywords:** brainstem glioma, child, white matter, tractography, DKI, diffusion MRI

## Abstract

**Background:**

Previous studies have identified alterations in structural connectivity of patients with glioma. However, white matter (WM) integrity measured by diffusion kurtosis imaging (DKI) in pediatric patients with brainstem glioma (BSG) was lack of study. Here, the alterations in WM of patients with BSG were assessed through DKI analyses.

**Materials and methods:**

This study involved 100 patients with BSG from the National Brain Tumor Registry of China (NBTRC) and 50 age- and sex-matched healthy controls from social recruitment. WM tracts were segmented and reconstructed using U-Net and probabilistic bundle-specific tracking. Next, automatic fiber quantitative (AFQ) analyses of WM tracts were performed using tractometry module embedded in TractSeg.

**Results:**

WM quantitative analysis identified alterations in DKI-derived values in patients with BSG compared with healthy controls. WM abnormalities were detected in the projection fibers involved in the brainstem, including corticospinal tract (CST), superior cerebellar peduncle (SCP), middle cerebellar peduncle (MCP) and inferior cerebellar peduncle (ICP). Significant WM alterations were also identified in commissural fibers and association fibers, which were away from tumor location. Statistical analyses indicated the severity of WM abnormality was statistically correlated with the preoperative Karnofsky Performance Scale (KPS) and symptom duration of patients respectively.

**Conclusion:**

The results of this study indicated the widely distributed WM alterations in patients with BSG. DKI-derived quantitative assessment may provide additional information and insight into comprehensively understanding the neuropathological mechanisms of brainstem glioma.

## Introduction

Brainstem gliomas (BSGs) are heterogeneous diseases that arise from the midbrain, pontine, and medulla oblongata. BSGs are characterized by clinical manifestations of cranial nerve deficits, long tract signs, and cerebellar signs (Korones, 2007). The Central Brain Tumor Registry of the United States (CBTRUS) reported that BSG takes about 1.5% of the primary central nervous system tumors (Ostrom et al., 2018). A total of 15–20% of BSGs in children are low-grade glial tumors, and the remaining 80% are diffusely located in the pontine, termed diffuse intrinsic pontine glioma (DIPG). The median age of onset is 6.5 years in children with DIPG (Fisher et al., 2000). The prognosis of children DIPG is poor, the 2-year mortality rate after diagnosis is of 90%, and the median survival is 9–12 months (Jones and Baker, 2014; Johung et al., 2017). Many types of therapies, including chemotherapy and small-molecule inhibitors, were identified to be ineffective, while radiotherapy only prolongs survival by a few months (Maria et al., 1993; Freeman and Perilongo, 1999). BSG involving the midbrain and medulla oblongata is still poorly characterized, mainly due to the rarity of the disease and the high risks of surgical resections and biopsies. The limitations of the current therapeutic strategy suggest that the underlying mechanism of tumor progression is not sufficiently elucidated, and the pathogenesis should be further investigated.

Previous studies have re-evaluated glioma as a systemic disease wherein tumor cells spread far beyond the macroscopically visible lesion and form a functional network throughout the entire brain (Sahm et al., 2012; Osswald et al., 2015). A postmortem study (Drumm et al., 2020) reported extensive brainstem infiltration along with destructions of the pontine and white matter (WM) tracts in patients with end-stage cerebral glioblastoma. Other studies (Hossain and Hrvoje, 2020) reported a similar cerebrum-to-brainstem infiltration pattern in the patient-derived xenograft (PDX) model. As the gateway connecting the cerebrum with the spine, multiple long tracts of myelinated axons extend from the cortex into the brainstem. Therefore, we hypothesized that the integrity of WM tracts connecting the brainstem and other brain regions could be compromised because of tumor.

To test our hypothesis, diffusion kurtosis imaging (DKI) was used to assess the changes in WM in patients with BSG. As a powerful imaging modality of diffusion magnetic resonance imaging (MRI), DKI provides great availabilities both for high diagnostic sensitivity and specificity in glioma grade differentiation and for WM tracts reconstruction (Maximov et al., 2017; Falk Delgado et al., 2018). Besides, to the best of our knowledge, no study has investigated the alterations in WM connectivity in BSG on a systemic level to date (Zhang et al., 2020). In this study, diffusion MRI techniques were used to identify the structural changes in patients with BSG compared with healthy controls. Moreover, the correlation between DKI-derived values and the preoperative Karnofsky Performance Scale (KPS) score as well as symptom duration time of patients were statistically analyzed respectively.

## Materials and methods

### Participants

**FIGURE 1:**
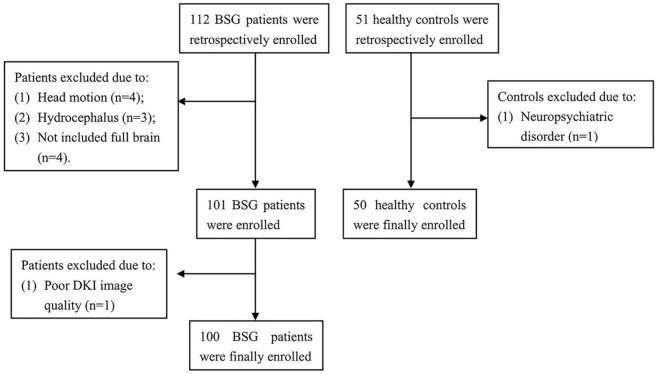
Flowchart of the study. BSG, brainstem glioma.

This study involved 112 BSG patients from the National Brain Tumor Registry of China (NBTRC) and 51 socially recruited healthy controls (Figure 1). The patients were recruited consecutively from December 2018 to October 2021 at the Neurosurgical Center of Beijing Tiantan Hospital, Capital Medical University, China. The demographic and clinical data (including age, gender, education level, handedness, main complaint, duration, and pathological diagnosis) were collected. Each case was histopathologically verified as BSG. The tumor-grade classification was made according to the revised version of the WHO classification system for CNS tumors of 2021 (Louis et al., 2021).

The inclusion criteria for BSG patients were as follows: (1) histological diagnosis of primary BSG; (2) children age 1–18 years; (3) prior MRI examination, including 3D T1 and DKI for statistical analyses. The exclusion criteria were as follows: (1) previous cranial surgery; (2) hydrocephalus; (3) history of traumatic brain injury; (4) neurological disorder; (5) neuropsychiatric comorbidities; (6) inability to undertake the MRI examination, and (7) any contraindications to MRI examination such as metal implants. (8) head motion exceeding ± 2 mm or ± 2°; (9) MRI examination not including the full brain.

Socially recruited healthy controls were required to fulfill the following inclusion criteria: (1) prior MRI examination, including 3D T1 and DKI; (2) children age 1–18 years. The exclusion criteria were as follows: (1) neurological disorder; (2) neuropsychiatric comorbidities; (3) head motion exceeding ± 2 mm or ± 2°; (4) MRI examination not including the full brain.

### Image acquisition

MRI examination of all participants was performed on a 3-T scanner (Ingenia CX, Philips Healthcare, Best, the Netherlands) with a 32-channel head receiver coil. As a result, the following sequences were acquired: sagittal 3D T1-weighted (3D-T1), post-contrast sagittal 3D T1-weighted (3D-T1 C+) images, and axial DKI.

The detailed imaging parameters were as follows: (1) 3D-T1: repetition time (TR) = 6.572 ms, echo time (TE) = 3.025 ms, flip angle (FA) = 8°, number of slices = 196, slice thickness = 1 mm, in-plane resolution = 1 × 1 mm. (2) DKI: TR/TE = 4,000/86.608 ms, flip angle (FA) = 90°, number of slices = 60, slice thickness = 2.5 mm, in-plane resolution = 2.27 × 2.27 mm, 97 directions (using *b*-value of 0 × 1 direction, 1,000 s/mm^2^ × 48 direction, and 2,000 s/mm^2^ × 48 directions).

### Diffusion kurtosis imaging preprocessing

After converting image data from DICOM to 4D Nifti format using dcm2niigui embedded in MRIcron, the preprocessing steps were conducted using Functional Magnetic Resonance Imaging of the Brain (FMRIB) Software Library (FSL, version 5.0.9) (Smith et al., 2004). The correction for the distortion of eddy current and head movement was applied, and the non-brain tissue was removed using a non-brain mask of the FSL Brain Extraction Tool (Smith, 2002). Then, the DKI-derived maps of fractional anisotropy (FA), mean diffusivity (MD), axial diffusivity (AD), radial diffusivity (RD), axial kurtosis (AK), radial kurtosis (RK), mean kurtosis (MK), and fractional axial kurtosis (Fak) were calculated using the Diffusion Kurtosis Estimator (DKE) (Tabesh et al., 2011).

### Tractography and quantitative analyses

WM bundles were segmented and reconstructed using the U-Net and probabilistic bundle-specific tracking method with TractSeg (Wasserthal et al., 2018, 2019). In detail, after Normalizing the Diffusion images to the standard Montreal Neurological Institute (MNI) coordinates using the linear registration tool embedded in FSL with 12 degrees-of-freedom, WM bundles were segmented using U-Net, and endpoint regions where bundle starting and ending were segmented using TractSeg. Then, tract orientation maps (TOMs) were created, which represent the main orientation of WM bundles per voxel. Then, the probabilistic fiber tracking was conducted based on TOMs, bundle masks, and endpoint regions. For each WM tract, the number of track was set at 5000 streamlines. The probabilistic fiber tracking procedure was conducted using RTX2060, NVIDIA graphic processing units (GPUs), the average time for tracking one fiber was 0.4–1.0 min. Next, automatic fiber quantitative (AFQ) analyses of specific WM tracts were performed using tractometry module, each fiber was resampled to 100 equally spaced nodes, then, diffusion properties were compared at each node of each fiber between patients and controls (Yeatman et al., 2012; Wasserthal et al., 2020).

### Statistical analysis

After the normality test, Spearman’s correlational analyses was performed between the severity of WM alterations and the preoperative KPS score as well as symptom duration time of patients before diagnosis. A two-sample *t*-test was applied to evaluate the differences in the DKI-derived values between patients and healthy controls [Family-wise error (FWE) corrected]. The significance value was set at *P*< 0.05.

## Results

### Demographic data and clinical information

A total of 112 patients with BSG and 51 healthy individuals were initially included in this retrospective study. In this cohort, 4 patients were excluded because of considerably head motion, 3 patients were excluded because of hydrocephalus, 4 patients were excluded because the MRI scan did not include the full brain, 1 patient was excluded because of poor image quality, and 1 control was excluded because of neuropsychiatric comorbidities. Finally, 100 patients and 50 healthy controls were included. Demographic and clinical characteristics of the 100 patients (51 females and 49 males, mean age: 8.72 ± 4.09 years) and 50 healthy controls (17 females and 33 males, mean age: 8.98 ± 2.96 years) were described in Table 1. All participants were right-handed. No significant difference was found in the age (*P* = 0.66), sex ratio (*P* = 0.05), and education level (*P* = 0.74) between patients and controls.

**TABLE 1:** Demographic and clinical characteristics of patients and healthy controls.

Variable	Patients (*n* = 100)	Healthy controls (*n* = 50)	*P*-value
Sex, *n* (%)			0.05
Male	49 (49%)	33 (33%)	
Female	51 (51%)	17 (17%)	
Age, years			0.66
Median (range)	8.00 (1–18)	9.00 (4–18)	
Mean ± SD	8.72 ± 4.09	8.98 ± 2.96	
Education (years)			0.74
Median (range)	3.00 (0–12)	4.00 (0–12)	
Mean ± SD	3.74 ± 3.72	3.92 ± 2.80	
Right-handedness	100 (100%)	50 (100%)	–
Main complaints			
Ataxia	53 (53%)	–	–
Choking	46 (46%)	–	–
Motor disorder	42 (42%)	–	–
Diplopia	38 (38%)	-	–
Dysphasia	31 (31%)	–	–
Headache	30 (30%)	–	–
Dysphagia	25 (25%)	–	–
Nausea	25 (25%)	–	–
Duration (months)			–
Median (range)	2.00 (0.25–74)	–	
Mean ± SD	5.14 ± 12.02	–	
KPS score			-
Median (range)	70 (40–100)	-	
Mean ± SD	67.20 ± 14.47	-	
WHO grade			
I	12 (12%)	-	-
II	10 (10%)	-	-
III	1 (1%)	-	-
IV	77 (77%)	-	-

In the patients’ group, the major symptoms were ataxia (53 patients, 53%) caused by deep sensory deficit, choking (46 patients, 46%) caused by lower cranial nerve dysfunction, and motor disorder (42 patients, 42%) caused by motor function deficit. The next common symptoms were diplopia (38 patients, 38%) caused by oculomotor nerve dysfunction, dysphasia (31 patients, 31%) caused by lower cranial nerve dysfunction, and headache associated with increased intracranial pressure (30 patients, 30%). Other common symptoms included dysphagia (25 patients, 25%) and nausea (25 patients, 25%) associated with the dysfunction of lower cranial nerve and autonomic nervous system. The mean duration time of symptoms was 5.14 ± 12.02 months (range: 0.25–74 months). The mean preoperative KPS score was 67.20 ± 14.47 (range: 40–100). A majority of the patients (77/100, 77%) were diagnosed as diffuse midline glioma with H3K27M mutant (WHO grade IV), 1 patient did not harbor H3K27M mutation and was diagnosed as anaplastic astrocytoma (WHO grade III), the remaining patients (22/100, 22%) were diagnosed as low-grade glioma (WHO grade I–II).

### Tractography-based white matter tracts quantitative analysis

**FIGURE 2:**
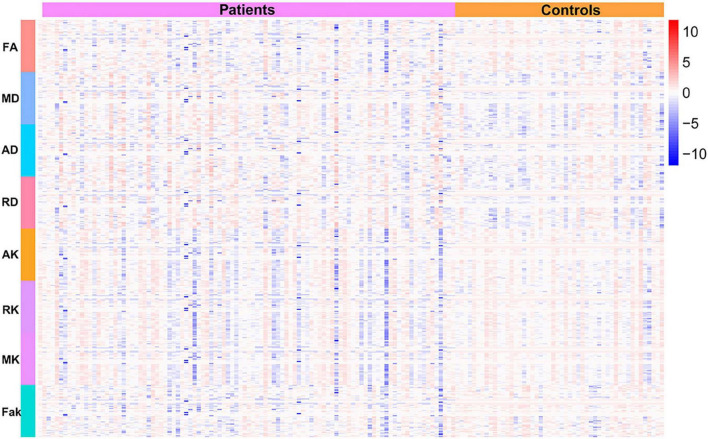
Heat map of the DKI-derived values in 150 participants.

Using the protocol illustrated in the methods, the probabilistic fiber tracking works well for all bundles except for the corticospinal tract (CST), fronto-pontine tract (FPT), inferior cerebellar peduncle (ICP), middle cerebellar peduncle (MCP), parieto-occipital pontine tract (POPT), and the superior cerebellar peduncle (SCP) which were incomplete sometimes, the details of reconstructed tracts were listed in Supplementary File 1. WM quantitative analysis showed alterations in DKI-derived values (including FA, MD, AD, RD, AK, RK, MK, and Fak) in patients with BSG compared with healthy controls (Figure 2).

**FIGURE 3:**
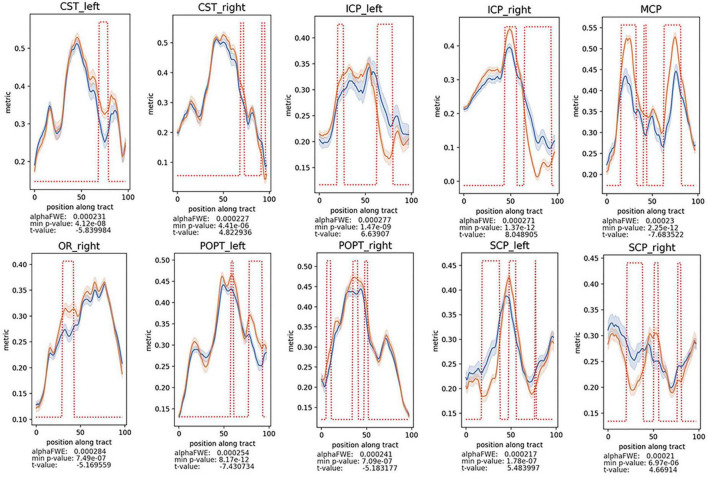
Decreased FA values of the CST, cerebrospinal tract; MCP, middle cerebellar peduncle; ICP, inferior cerebellar peduncle; OR, optic radiation; POPT, parieto-occipital pontine tract; SCP, superior cerebellar peduncle, were found in patients vs. healthy controls (*P* < 0.05, FWE-corrected). The alphaFWE is the alpha value corrected for multiple comparison (multiple parts per tract and multiple tracts). The min *p*-value is the minimal *p*-value which was calculated for each tract. If min *p*-value < alphaFWE, the tract contains significant results. The red dotted lines above of the lines of the patients’ group and the controls indicates all positions with the tract where *p*-value < alphaFWE.

**FIGURE 4:**
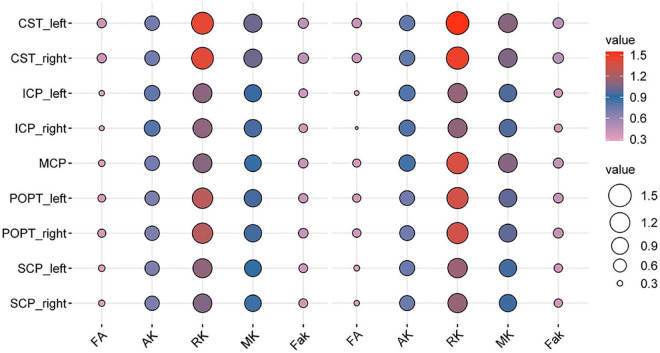
Bubble plot of the DKI-derived values of the specific white matter (WM) tracts between the patients with BSG and controls (the left five columns were the DKI-derived values of the patients’ group, the right five columns were the DKI-derived values of healthy controls).

To further illustrate the difference of WM alterations between the two groups and which part of the tract could be responsible for these differences, two-sample *t*-test on the tractometry data was performed (Figure 3 and Supplementary Figures 1–8). Considering that the gender difference between the two groups, we conducted analysis between groups considering factors including age, gender, and education level as covariates. After analysis, decreased FA values of these following WM tracts were identified in the patients’ group: anterior thalamic radiation (ATR), callous corpus (CC), CST, FPT, ICP, MCP, optic radiation (OR), parieto-occipital pontine tract (POPT), SCP, superior longitudinal fascicle (SLF), thalamo-occipital (T_OCC), and striato-fronto-orbital (ST_FO) (*P* < 0.05, FWE-corrected). And the AK, RK, MK, and Fak values of most of the above-mentioned tracts were also identified as significantly decreased, similar to the results of FA values (Figures 3, 4 and Supplementary Figures 1–8). Besides, increased MD, AD, and RD values of the following tracts were identified in patients’ group, including: CC, CST, FPT, ICP, inferior occipito-frontal fascicle (IFO), MCP, POPT, SCP, thalamo-parietal (T_PAR), and T_OCC (Supplementary Figures 2–4).

Interestingly, consistent alterations of decreased FA, AK, RK, MK, Fak and increased MD, AD, RD were identified in CST and MCP. In addition, bidirectional alterations of FA, MD, AD, RD, AK, RK, MK, and Fak were identified in bilateral ICP and SCP (Supplementary Figures 1–8).

### Correlation test between diffusion kurtosis imaging-derived values and clinical data

**FIGURE 5:**
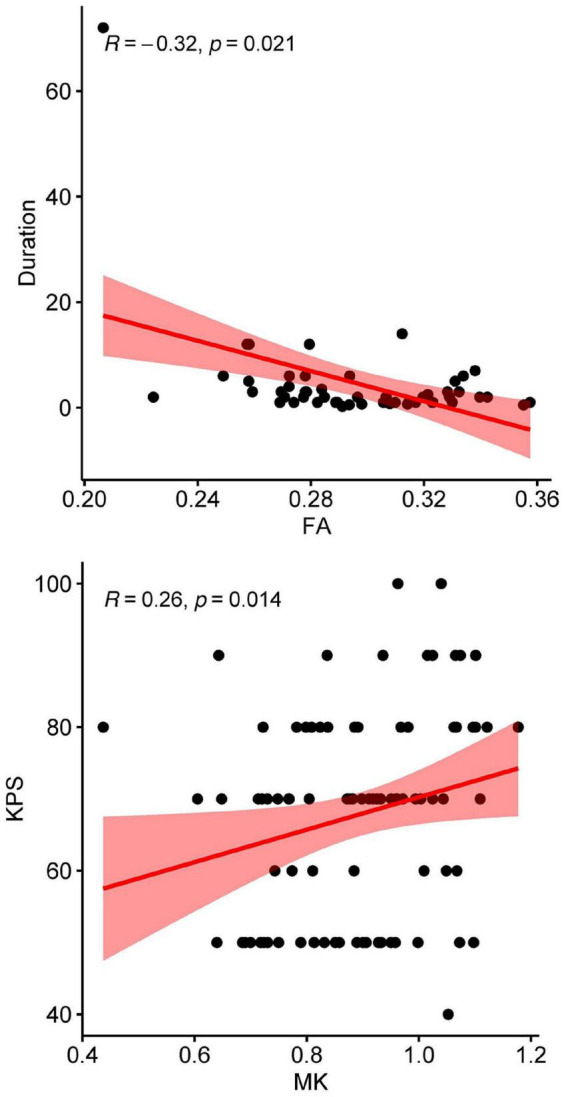
Correlation between the DKI-derived values and symptom duration and KPS scores of patients: top: correlation between FA of the right ICP and symptom duration; bottom: correlation between MK of the MCP and the preoperative KPS score (as right ICP in 43 cases failed to be reconstructed, the right ICP of 57 cases were used for correlation analysis).

DKI-derived values were stratified analyzed based on tumor grade (low grade glioma vs. high grade glioma, LGG vs. HGG). Decreased FA, AK, RK, and MK values of CST, MCP, POPT were identified in HGG compared with LGG (Supplementary Figures 9–16). The results of Spearman’s correlational analyses showed that the abnormally higher FA value of the right ICP was negatively associated with symptom duration (*r* = –0.32, *P* = 0.021) among patients’ group, MK of MCP was positively associated with preoperative KPS score (*r* = 0.26, *P* = 0.014) among patients’ group. The higher AK value of MCP was positively associated with preoperative KPS score (*r* = 0.26, *P* = 0.016). The higher RK value of MCP was positively associated with preoperative KPS score (*r* = 0.25, *P* = 0.019) (Figure 5).

## Discussion

In this study, we analyzed structural alterations in patients with BSG. The major findings were as follows: The quantitative measurements of WM tracts demonstrated a significant decrease in FA, AK, RK, MK, and Fak with a concomitant increase in RD, AD, and MD in patients with BSG. These alterations were correlated with pathological features of the disease. The quantitative measurements of WM tracts quantified undetectable disruptions of WM tracts along the cerebrum and cerebellum. The DKI-derived values were correlated with symptom duration time and preoperative KPS score.

### Diffusion kurtosis imaging alterations in whole brain white matter tracts in brainstem glioma

Anatomically, the brainstem serves as a gateway for all ascending, descending, and traversed tracts connecting the cerebrum, spinal cord, and cerebellum. The descending tracts include the corticospinal, frontopontine, parietal-pontine, occipitopontine, and temporopontine tracts arising from the cortex. The ascending tracts include the spinothalamic tracts, medial lemniscus, and lateral lemniscus, as well as traversed superior, middle, and inferior cerebellar peduncles connecting the brainstem with the cerebellum. Therefore, the BSGs tumor cells have the possibility of infiltrating and migrating along WM tracts.

Our results identified alterations in the widespread WM tracts in patients with BSG, including the association, commissural, and projection fibers, which may indicate axonal damage and myelin loss (Figures 2–4 and Supplementary Figures 1–8). Previous studies have identified a series of tract alteration patterns including deviation, deformation, infiltration, and apparent tract interruption in adult brainstem tumors (Chen et al., 2007). Recent studies showed diffusion MRI was powerful in tumor grade classification, prediction of molecular biomarker, patients’ therapy response and functional recovery (Lupo, 2020; Jiang et al., 2021; Sun et al., 2022). One previous study of our team has identified clinically that CST could be damaged by BSGs both in adults and in pediatric patients using DTI techniques (Xiao et al., 2021). The alterations of widespread tracts may influence the function of the entire brain, which needs further investigation. Previous studies mainly focused on the area of interest in solid tumoral lesion or WM tracts around the tumor, but seldomly considered the whole brain structural connectivity alterations due to primary tumor. Recent studies have re-evaluated glioma as a systemic disease with tumor cells spreading far beyond the macroscopically visible lesion along tracts, perivascular space or meninges with specific biological mechanisms (Cuddapah et al., 2014; Caverzasi et al., 2016; Sharifi et al., 2019; Wang et al., 2019). Glioma cells also biologically connect with neuron and behave invasiveness, which could further influence the structure-function networks throughout the whole brain (Venkataramani et al., 2019, 2022; Venkatesh et al., 2019). A postmortem study of patients with cerebral glioblastoma showed tumor cells infiltrating along the cerebral WM tracts into the brainstem, and the infiltrating tumor cells within the brainstem had atypical nuclei similar to original tumor cells (Drumm et al., 2020). This extensive infiltration property was confirmed by another study, which showed similar brainstem infiltration in the cerebrum GBM PDX-derived models, and the infiltrating tumor cells in the mesencephalon/pontine had severe invasive features than primary tumors with significant less angiogenesis (Hossain and Hrvoje, 2020). These results indicated that fibers away from the tumor could also be affected by tumor infiltrating and migrating, which is consistent with this study and needs further investigation in the future.

### Alterations in diffusion kurtosis imaging-derived values and possible neurophysiological mechanism

The quantitative measurements of DKI-derived values of WM tracts showed significant WM alterations in patients with BSG (Figures 2–4 and Supplementary Figures 1–8). DKI is an extension of conventional diffusion tensor imaging (DTI). DTI assumes the diffusion of water molecules to be random and unrestricted but potentially hindered process. The diffusion probability distribution function (PDF) of water molecules is considered Gaussian PDF. The presence of cell membranes, intracellular organelles, and the rapid exchange of protons among different biologic cytoarchitectures can restrict the random Brownian motion of water molecules (Le Bihan et al., 1992). The deviation from Gaussian PDF can be measured using the apparent excess kurtosis coefficient (AKC). Several diffusion parameters can be derived from DKI, which include conventional DTI parameters (FA, AD, RD, and MD) and DKI-specific kurtosis parameters (Fak, AK, RK, and MK).

In this study, we found a significant decrease in FA, AK, RK, MK, and Fak values with a concomitant increase in RD, AD, and MD values in patients with BSG. FA is a marker of WM integrity, and reduced FA values have been found in patients with pontine tumors, which indicate the impairment of the integrity of WM. A positive correlation between FA values and tumor cell density has been reported (Helton et al., 2006; Kinoshita et al., 2008). AD and AK indicate axonal integrity and WM density. RD and RK indicate myelin integrity and axonal density (Mori et al., 2002; Alexander et al., 2007; Wu and Cheung, 2010). Increased AD, RD, and MD and decreased AK, RK, and MK may correlate with axonal damage, demyelination, gliomas invasive growth, and progression. Previous research has reported electrochemical communication between neuron and glioma cell through bona fide AMPA receptor-dependent neuron-glioma synapses, which indicated that the synaptic and electrical integration in neural circuits promotes glioma progression (Venkataramani et al., 2019, 2022; Venkatesh et al., 2019). The alterations of DKI-derived parameters indicated the extensive impairments of specific tracts in patients with BSG, with underlying mechanisms need to be further investigated. Besides, the next-generation sequencing technology has revealed that the BSGs frequently harbor *H3K27M*, *TP53*, and *ATRX* mutation, which is different from cerebral gliomas that harbored IDH-mutant frequently (Wu et al., 2012, 2014; Saratsis et al., 2014; Mackay et al., 2017). Whether these specific genetic mutations can affect the integrity of WM is still unclear, and needs further investigation.

### Diffusion kurtosis imaging-derived values correlated with preoperative status

The results of correlation analyses between the DKI-derived values and preoperative status showed that the quantitative measurement of WM tracts can serve as an imaging biomarker for evaluating preoperative status. The Spearman’s correlation analyses results showed a positive correlation between AK, RK, and MK values of MCP and preoperative KPS scores. These results are reasonable, because higher AK, RK and MK values indicate better axonal integrity and less damage of the whole brain structures, correlated with better brain function. And higher KPS score also indicate a better overall functional status. Therefore, higher AK, RK and MK values should be in accordance with higher preoperative KPS scores, which is in accordance with this present study. MCP is one of the major tracts located in the brainstem, consisting of the transversely coursing pontocerebellar fibers that arch across the midline and gather on each side, and it serves as an important hub connecting the bilateral cerebellum. Therefore, MCP was commonly influenced by BSGs, besides, tracts (CST, POPT, SCP, and ICP) anatomically connected with brainstem were also easily to be influenced by tumor migrating and infiltrating (Figure 5).

The study also indicate higher FA value of the right ICP was associated with shorter symptom duration in patients’ group. Higher value of FA indicated a less severe damage of ICP fibers, and a short symptom duration before diagnosis indicated that tumor may have not invaded the fibers nearby in a relative short time, which means the fibers among tumors (such as MCP) may firstly be affected, and then the fibers nearby the tumor (such as ICP) was secondly affected (Wang et al., 2019). The underlying mechanisms are still not clear and need further investigation.

The comparisons of DKI-derived parameters between LGG and HGG identified decreased FA, AK, RK, and MK values of CST, MCP, POPT in HGG compared with LGG (Supplementary Figures 9–16). The results indicate the axon integrity was more severely damaged in HGG group.

## Conclusion

The results of our study showed the widely distributed WM alterations in patients with BSG. DKI-derived diffusion parameters were correlated with patient symptom duration time and preoperative KPS score, indicating less damage to WM tracts integrity was associated with better clinical status. The new insights from our study provide a fundamental perspective on BSGs. Brainstem dysfunction is a common cause of disability and mortality of patients, the presumable extensive infiltration of tumor to cerebrum and cerebellum may also serve as important influencing factors contributing to disease progression and death (Zhang et al., 2020). Until now, a variety of therapies failed to improve overall survival in patients with BSG, although some encouraging improvements were achieved in preclinical studies. We postulated that PDX models or transgenic models are not sufficient enough to recapitulate tumor infiltration features and widespread WM alterations characteristics. Additionally, traditional radiologic evaluation of BSGs may be inadequate in evaluating disease progression and therapeutic effectiveness. Based on these assumptions, further investigations are needed to explore the possible mechanisms and therapeutic biomarkers of tumor infiltrating and migrating through humanized animal models and preclinical studies.

## Data availability statement

The original contributions presented in this study are included in the article/Supplementary material, further inquiries can be directed to the corresponding author/s.

## Ethics statement

The studies involving human participants were reviewed and approved by the Ethics Committee of Beijing Tiantan Hospital, Capital Medical University. Written informed consent to participate in this study was provided by the participants’ legal guardian/next of kin.

## Author contributions

PZ and LZ carried out the research conceptualization, data collecting and analyzing, manuscript drafting, editing and proofs, and funds supporting. GG carried out the data collecting and analyzing, manuscript drafting, and editing. YD and ZZ carried out the data collecting supporting. CP, PCZ, YW, XL, ZJ, and LQ carried out data collecting and analyzing. YL carried out data collecting and editing of manuscript. All authors contributed to the article and approved the submitted version.
