# Supplementary material for: White matter alterations in pediatric brainstem glioma: An national brain tumor registry of China study
Source: Front Neurosci. 2022 Sep 9;16:986873. doi: 10.3389/fnins.2022.986873 (PMC9500240; doi:10.3389/fnins.2022.986873)
Supplement: Supplementary file 1 [file Data_Sheet_1.pdf]

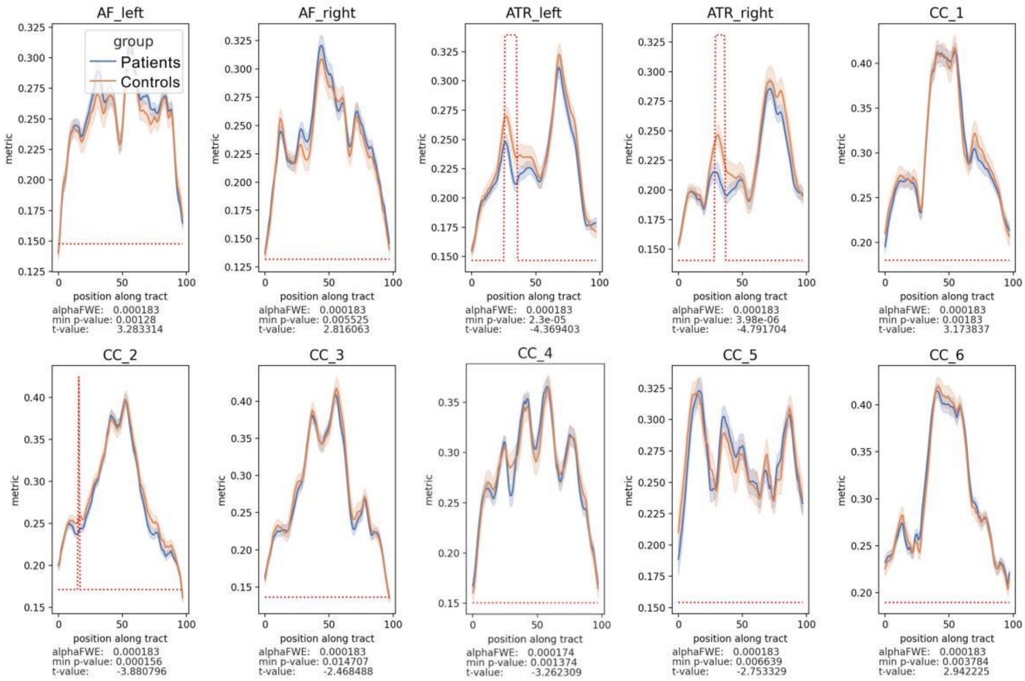
AF_left
group
Patients
Controls
position along tract
metric
alphaFWE: 0.000183
min p-value: 0.00128
t-value: 3.283314
AF_right
alphaFWE: 0.000183
min p-value: 0.005525
t-value: 2.816063
ATR_left
alphaFWE: 0.000183
min p-value: 2.3e-05
t-value: -4.369403
ATR_right
alphaFWE: 0.000183
min p-value: 3.98e-06
t-value: -4.791704
CC_1
alphaFWE: 0.000183
min p-value: 0.00183
t-value: 3.173837
CC_2
alphaFWE: 0.000183
min p-value: 0.000156
t-value: -3.880796
CC_3
alphaFWE: 0.000183
min p-value: 0.014707
t-value: -2.468488
CC_4
alphaFWE: 0.000174
min p-value: 0.001374
t-value: -3.262309
CC_5
alphaFWE: 0.000183
min p-value: 0.006639
t-value: -2.753329
CC_6
alphaFWE: 0.000183
min p-value: 0.003784
t-value: 2.942225

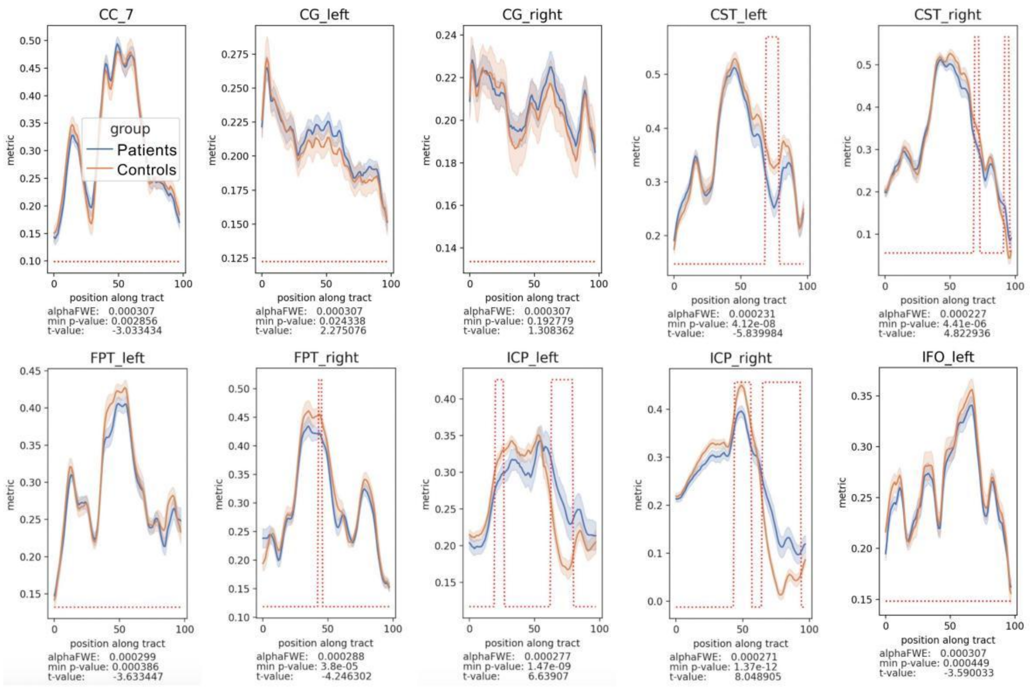
CC_7
group
Patients
Controls
position along tract
metric
alphaFWE: 0.000307
min p-value: 0.002856
t-value: -3.033434
CG_left
alphaFWE: 0.000307
min p-value: 0.024338
t-value: 2.275076
CG_right
alphaFWE: 0.000307
min p-value: 0.192779
t-value: 1.308362
CST_left
alphaFWE: 0.000231
min p-value: 4.12e-08
t-value: -5.839984
CST_right
alphaFWE: 0.000227
min p-value: 4.41e-06
t-value: 4.822936
FPT_left
alphaFWE: 0.000299
min p-value: 0.000386
t-value: -3.633447
FPT_right
alphaFWE: 0.000288
min p-value: 3.8e-05
t-value: -4.246302
ICP_left
alphaFWE: 0.000277
min p-value: 1.47e-09
t-value: 6.63907
ICP_right
alphaFWE: 0.000271
min p-value: 1.37e-12
t-value: 8.048905
IFO_left
alphaFWE: 0.000307
min p-value: 0.000449
t-value: -3.590033

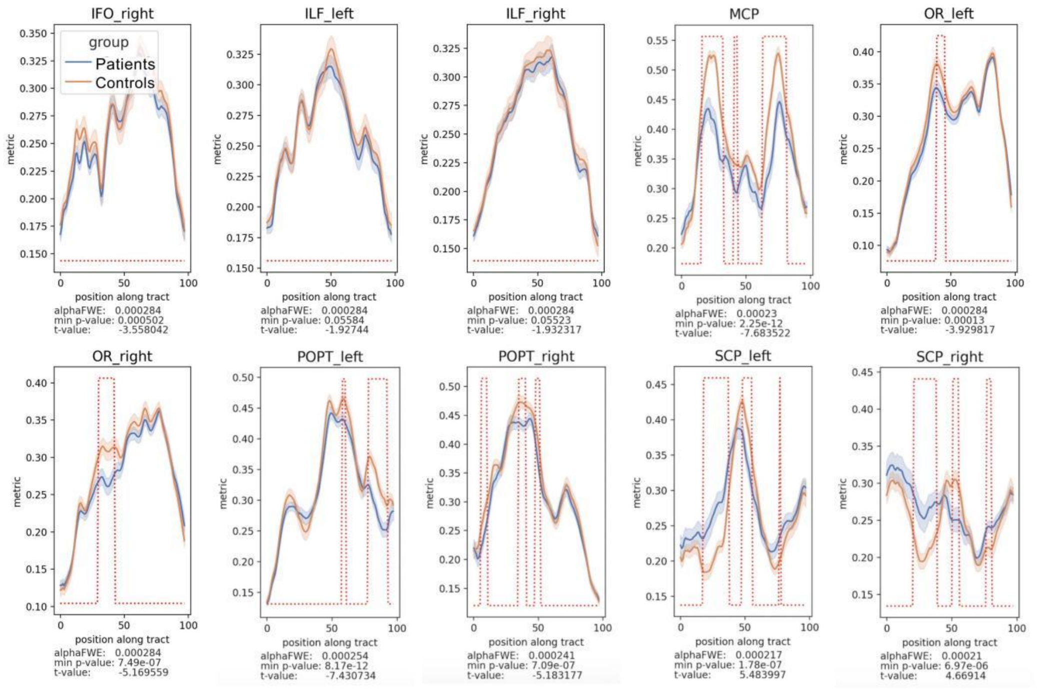
IFO_right
group
Patients
Controls
metric
position along tract
alphaFWE: 0.000284
min p-value: 0.000502
t-value: -3.558042
ILF_left
alphaFWE: 0.000284
min p-value: 0.05584
t-value: -1.92744
ILF_right
alphaFWE: 0.000284
min p-value: 0.05523
t-value: -1.932317
MCP
alphaFWE: 0.00023
min p-value: 2.25e-12
t-value: -7.683522
OR_left
alphaFWE: 0.000284
min p-value: 0.00013
t-value: -3.929817
OR_right
alphaFWE: 0.000284
min p-value: 7.49e-07
t-value: -5.169559
POPT_left
alphaFWE: 0.000254
min p-value: 8.17e-12
t-value: -7.430734
POPT_right
alphaFWE: 0.000241
min p-value: 7.09e-07
t-value: -5.183177
SCP_left
alphaFWE: 0.000217
min p-value: 1.78e-07
t-value: 5.483997
SCP_right
alphaFWE: 0.00021
min p-value: 6.97e-06
t-value: 4.66914

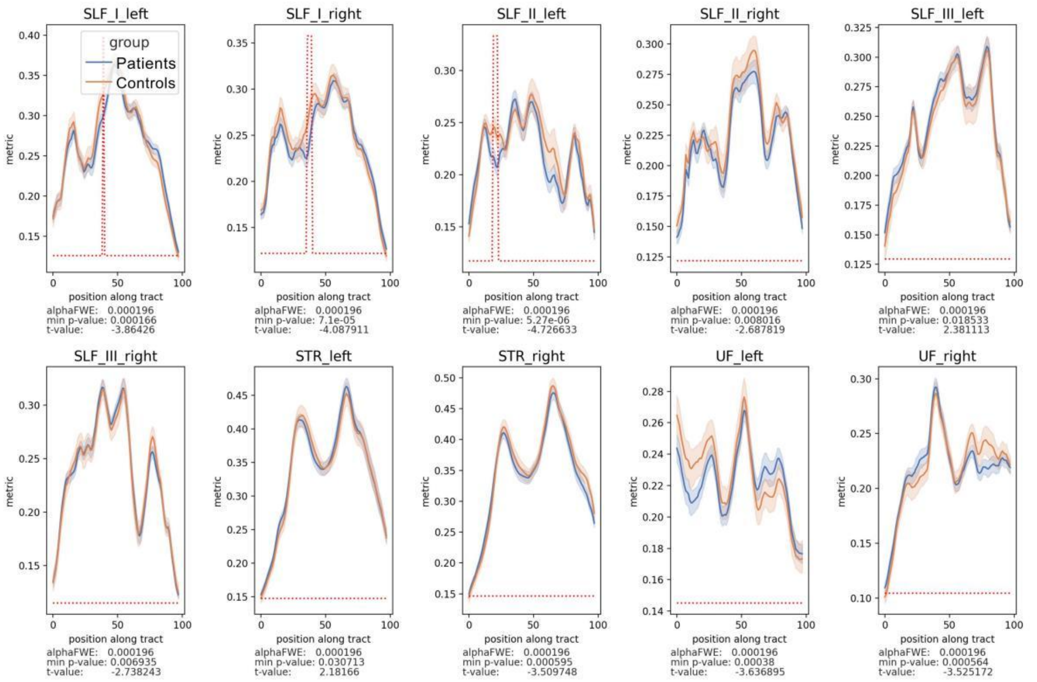
SLF_I_left
group
Patients
Controls
metric
position along tract
alphaFWE: 0.000196
min p-value: 0.000166
t-value: -3.86426
SLF_I_right
alphaFWE: 0.000196
min p-value: 7.1e-05
t-value: -4.087911
SLF_II_left
alphaFWE: 0.000196
min p-value: 5.27e-06
t-value: -4.726633
SLF_II_right
alphaFWE: 0.000196
min p-value: 0.008016
t-value: -2.687819
SLF_III_left
alphaFWE: 0.000196
min p-value: 0.018533
t-value: 2.381113
SLF_III_right
alphaFWE: 0.000196
min p-value: 0.006935
t-value: -2.738243
STR_left
alphaFWE: 0.000196
min p-value: 0.030713
t-value: 2.18166
STR_right
alphaFWE: 0.000196
min p-value: 0.000595
t-value: -3.509748
UF_left
alphaFWE: 0.000196
min p-value: 0.00038
t-value: -3.636895
UF_right
alphaFWE: 0.000196
min p-value: 0.000564
t-value: -3.525172

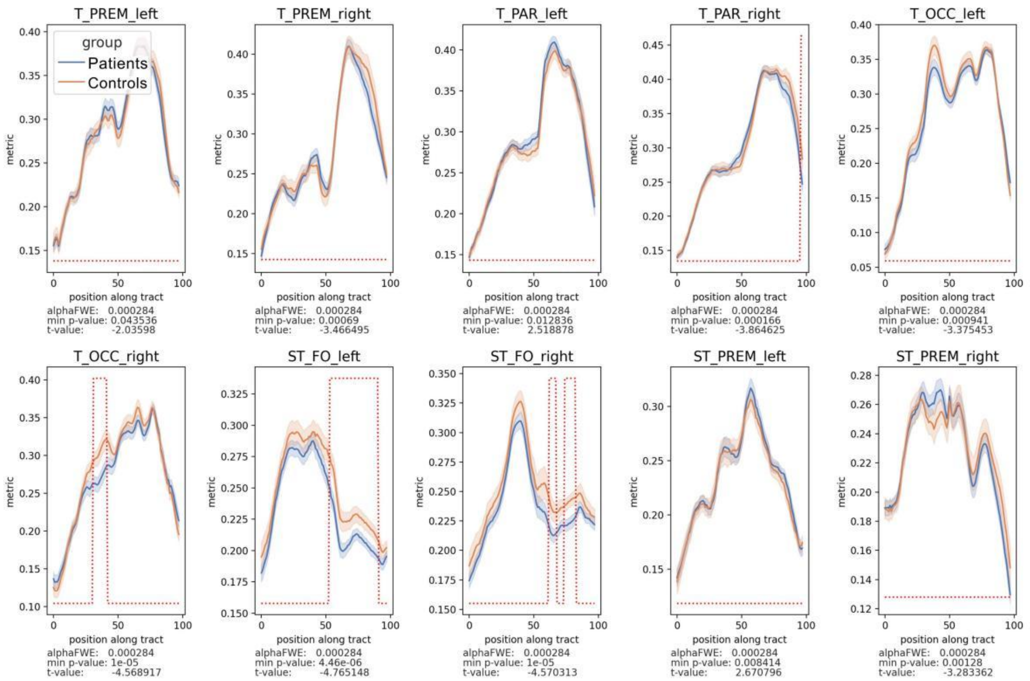

Supplementary Figure 1. Quantitative analysis of FA values of fifty white matter tracts in patients versus healthy controls.

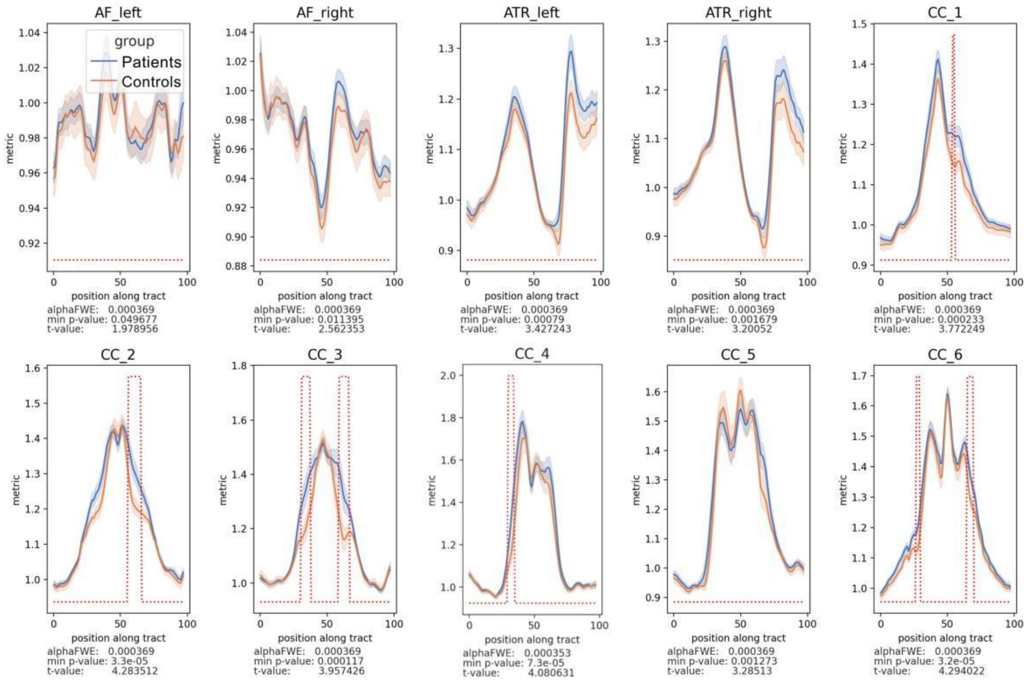
AF_left
AF_right
ATR_left
ATR_right
CC_1
group
Patients
Controls
metric
position along tract
alphaFWE: 0.000369
min p-value: 0.049677
t-value: 1.978956
alphaFWE: 0.000369
min p-value: 0.011395
t-value: 2.562353
alphaFWE: 0.000369
min p-value: 0.00079
t-value: 3.427243
alphaFWE: 0.000369
min p-value: 0.001679
t-value: 3.20052
alphaFWE: 0.000369
min p-value: 0.000233
t-value: 3.772249
CC_2
CC_3
CC_4
CC_5
CC_6
alphaFWE: 0.000369
min p-value: 3.3e-05
t-value: 4.283512
alphaFWE: 0.000369
min p-value: 0.000117
t-value: 3.957426
alphaFWE: 0.000353
min p-value: 7.3e-05
t-value: 4.080631
alphaFWE: 0.000369
min p-value: 0.001273
t-value: 3.28513
alphaFWE: 0.000369
min p-value: 3.2e-05
t-value: 4.294022

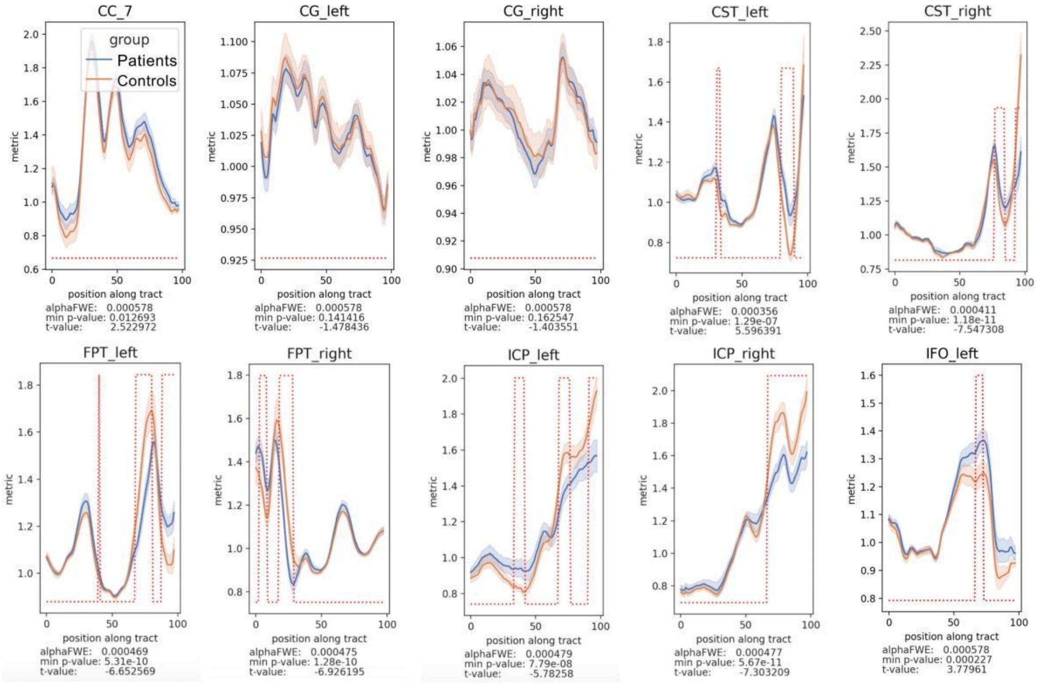
CC_7
CG_left
CG_right
CST_left
CST_right
group
Patients
Controls
metric
position along tract
alphaFWE: 0.000578
min p-value: 0.012693
t-value: 2.522972
alphaFWE: 0.000578
min p-value: 0.141416
t-value: -1.478436
alphaFWE: 0.000578
min p-value: 0.162547
t-value: -1.403551
alphaFWE: 0.000356
min p-value: 1.29e-07
t-value: 5.596391
alphaFWE: 0.000411
min p-value: 1.18e-11
t-value: -7.547308
FPT_left
FPT_right
ICP_left
ICP_right
IFO_left
alphaFWE: 0.000469
min p-value: 5.31e-10
t-value: -6.652569
alphaFWE: 0.000475
min p-value: 1.28e-10
t-value: -6.926195
alphaFWE: 0.000479
min p-value: 7.79e-08
t-value: -5.78258
alphaFWE: 0.000477
min p-value: 5.67e-11
t-value: -7.303209
alphaFWE: 0.000578
min p-value: 0.000227
t-value: 3.77961

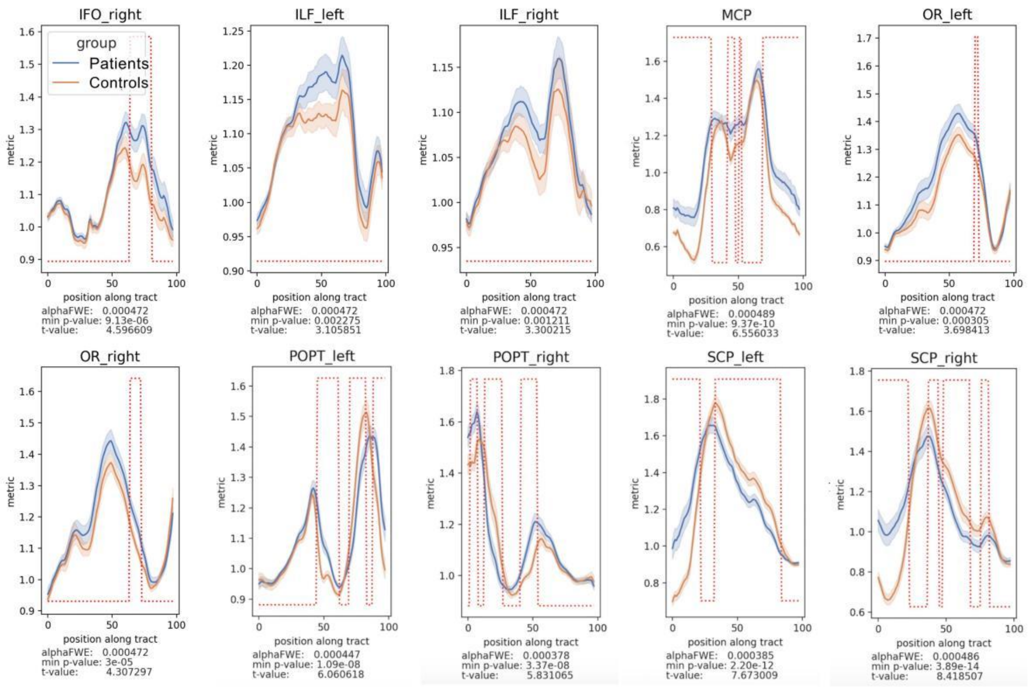
IFO_right
group
Patients
Controls
metric
position along tract
alphaFWE: 0.000472
min p-value: 9.13e-06
t-value: 4.596609
ILF_left
alphaFWE: 0.000472
min p-value: 0.002275
t-value: 3.105851
ILF_right
alphaFWE: 0.000472
min p-value: 0.001211
t-value: 3.300215
MCP
alphaFWE: 0.000489
min p-value: 9.37e-10
t-value: 6.556033
OR_left
alphaFWE: 0.000472
min p-value: 0.000305
t-value: 3.698413
OR_right
alphaFWE: 0.000472
min p-value: 3e-05
t-value: 4.307297
POPT_left
alphaFWE: 0.000447
min p-value: 1.09e-08
t-value: 6.060618
POPT_right
alphaFWE: 0.000378
min p-value: 3.37e-08
t-value: 5.831065
SCP_left
alphaFWE: 0.000385
min p-value: 2.20e-12
t-value: 7.673009
SCP_right
alphaFWE: 0.000486
min p-value: 3.89e-14
t-value: 8.418507

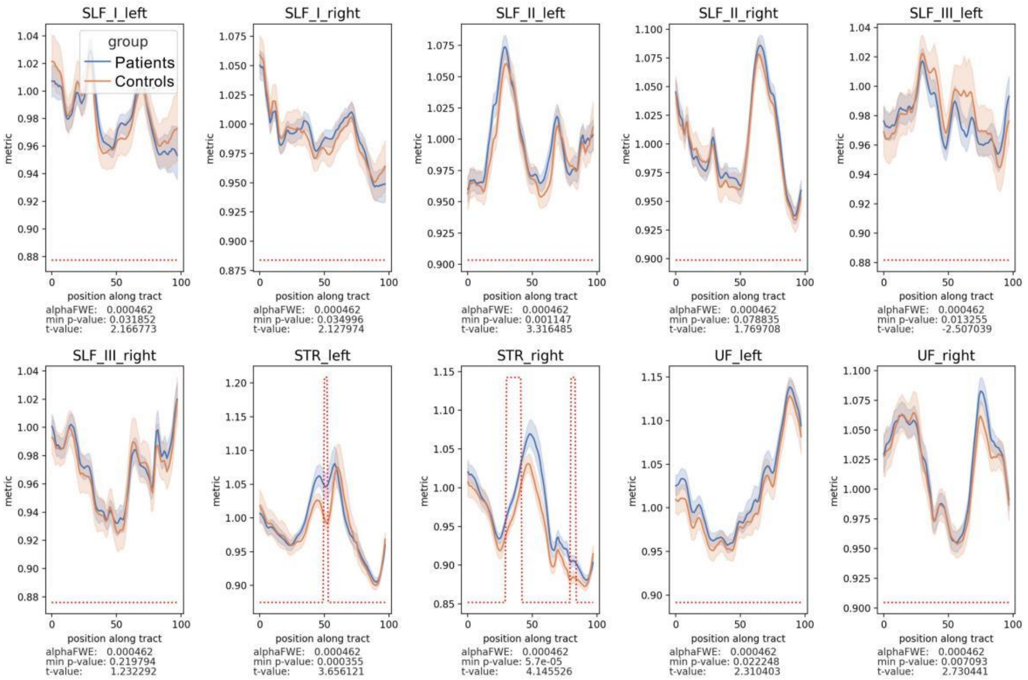
SLF_I_left
group
Patients
Controls
metric
position along tract
alphaFWE: 0.000462
min p-value: 0.031852
t-value: 2.166773
SLF_I_right
alphaFWE: 0.000462
min p-value: 0.034996
t-value: 2.127974
SLF_II_left
alphaFWE: 0.000462
min p-value: 0.001147
t-value: 3.316485
SLF_II_right
alphaFWE: 0.000462
min p-value: 0.078835
t-value: 1.769708
SLF_III_left
alphaFWE: 0.000462
min p-value: 0.013255
t-value: -2.507039
SLF_III_right
alphaFWE: 0.000462
min p-value: 0.219794
t-value: 1.232292
STR_left
alphaFWE: 0.000462
min p-value: 0.000355
t-value: 3.656121
STR_right
alphaFWE: 0.000462
min p-value: 5.7e-05
t-value: 4.145526
UF_left
alphaFWE: 0.000462
min p-value: 0.022248
t-value: 2.310403
UF_right
alphaFWE: 0.000462
min p-value: 0.007093
t-value: 2.730441

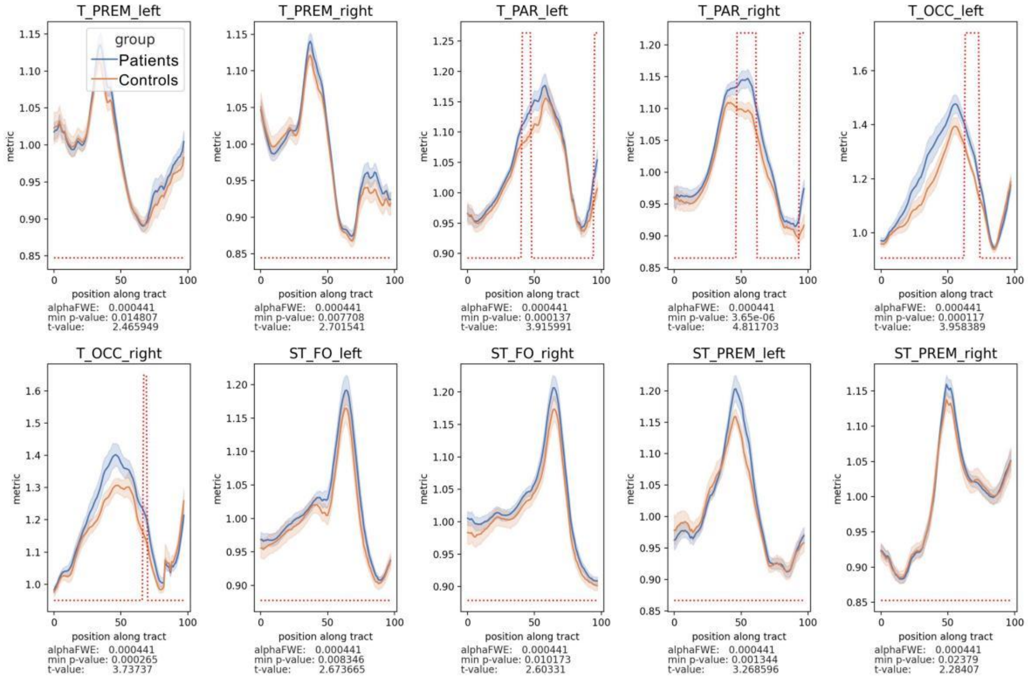

Supplementary Figure 2. Quantitative analysis of MD values of fifty white matter tracts in patients versus healthy controls.

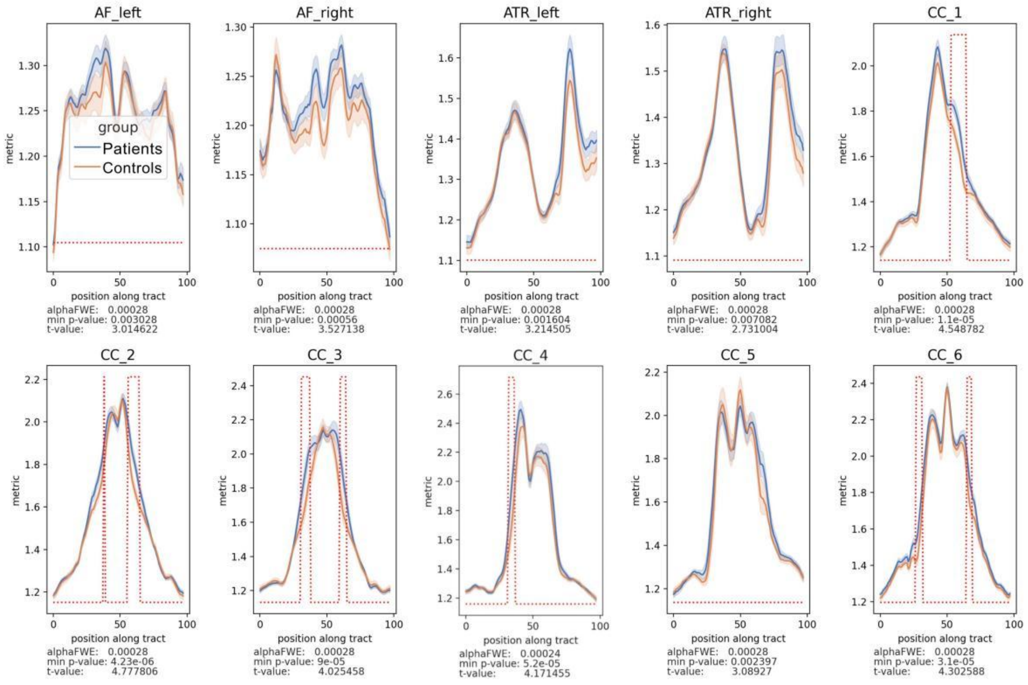
AF_left
group
Patients
Controls
metric
position along tract
alphaFWE: 0.00028
min p-value: 0.003028
t-value: 3.014622
AF_right
alphaFWE: 0.00028
min p-value: 0.00056
t-value: 3.527138
ATR_left
alphaFWE: 0.00028
min p-value: 0.001604
t-value: 3.214505
ATR_right
alphaFWE: 0.00028
min p-value: 0.007082
t-value: 2.731004
CC_1
alphaFWE: 0.00028
min p-value: 1.1e-05
t-value: 4.548782
CC_2
alphaFWE: 0.00028
min p-value: 4.23e-06
t-value: 4.777806
CC_3
alphaFWE: 0.00028
min p-value: 9e-05
t-value: 4.025458
CC_4
alphaFWE: 0.00024
min p-value: 5.2e-05
t-value: 4.171455
CC_5
alphaFWE: 0.00028
min p-value: 0.002397
t-value: 3.08927
CC_6
alphaFWE: 0.00028
min p-value: 3.1e-05
t-value: 4.302588

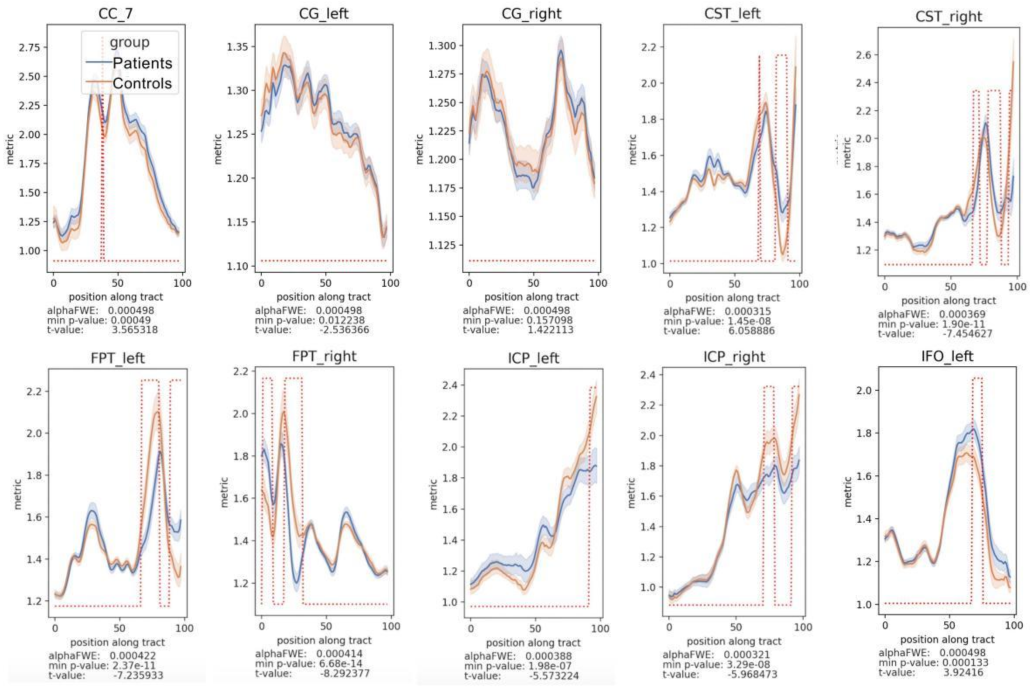
CC_7
group
Patients
Controls
metric
position along tract
alphaFWE: 0.000498
min p-value: 0.00049
t-value: 3.565318
CG_left
alphaFWE: 0.000498
min p-value: 0.012238
t-value: -2.536366
CG_right
alphaFWE: 0.000498
min p-value: 0.157098
t-value: 1.422113
CST_left
alphaFWE: 0.000315
min p-value: 1.45e-08
t-value: 6.058886
CST_right
alphaFWE: 0.000369
min p-value: 1.90e-11
t-value: -7.454627
FPT_left
alphaFWE: 0.000422
min p-value: 2.37e-11
t-value: -7.235933
FPT_right
alphaFWE: 0.000414
min p-value: 6.68e-14
t-value: -8.292377
ICP_left
alphaFWE: 0.000388
min p-value: 1.98e-07
t-value: -5.573224
ICP_right
alphaFWE: 0.000321
min p-value: 3.29e-08
t-value: -5.968473
IFO_left
alphaFWE: 0.000498
min p-value: 0.000133
t-value: 3.92416

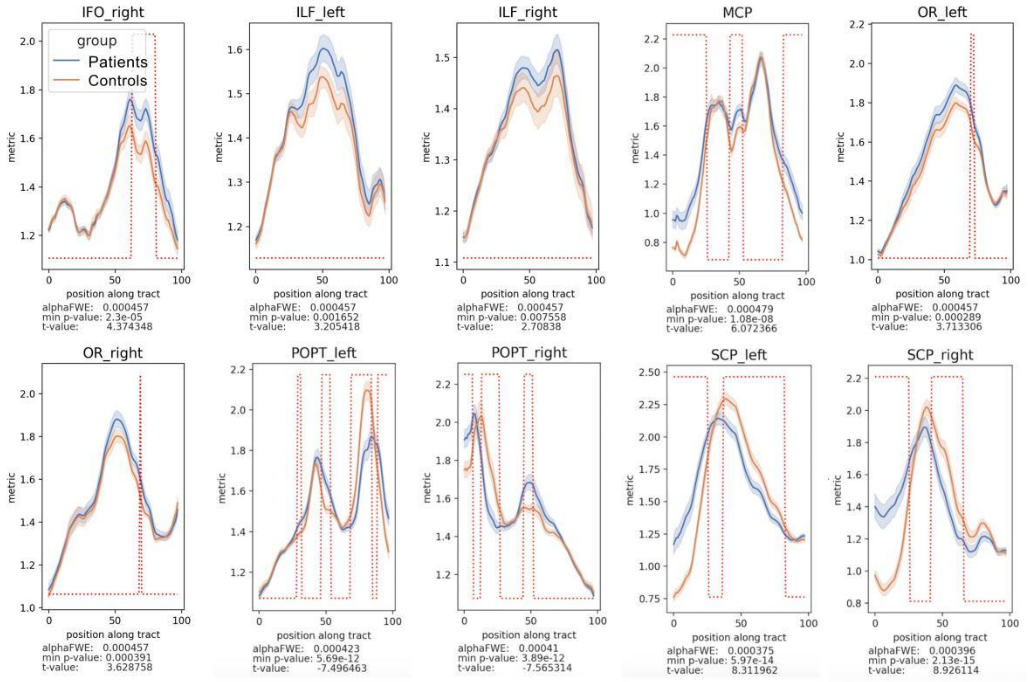
IFO_right
group
Patients
Controls
metric
position along tract
alphaFWE: 0.000457
min p-value: 2.3e-05
t-value: 4.374348
ILF_left
alphaFWE: 0.000457
min p-value: 0.001652
t-value: 3.205418
ILF_right
alphaFWE: 0.000457
min p-value: 0.007558
t-value: 2.70838
MCP
alphaFWE: 0.000479
min p-value: 1.08e-08
t-value: 6.072366
OR_left
alphaFWE: 0.000457
min p-value: 0.000289
t-value: 3.713306
OR_right
alphaFWE: 0.000457
min p-value: 0.000391
t-value: 3.628758
POPT_left
alphaFWE: 0.000423
min p-value: 5.69e-12
t-value: -7.496463
POPT_right
alphaFWE: 0.00041
min p-value: 3.89e-12
t-value: -7.565314
SCP_left
alphaFWE: 0.000375
min p-value: 5.97e-14
t-value: 8.311962
SCP_right
alphaFWE: 0.000396
min p-value: 2.13e-15
t-value: 8.926114

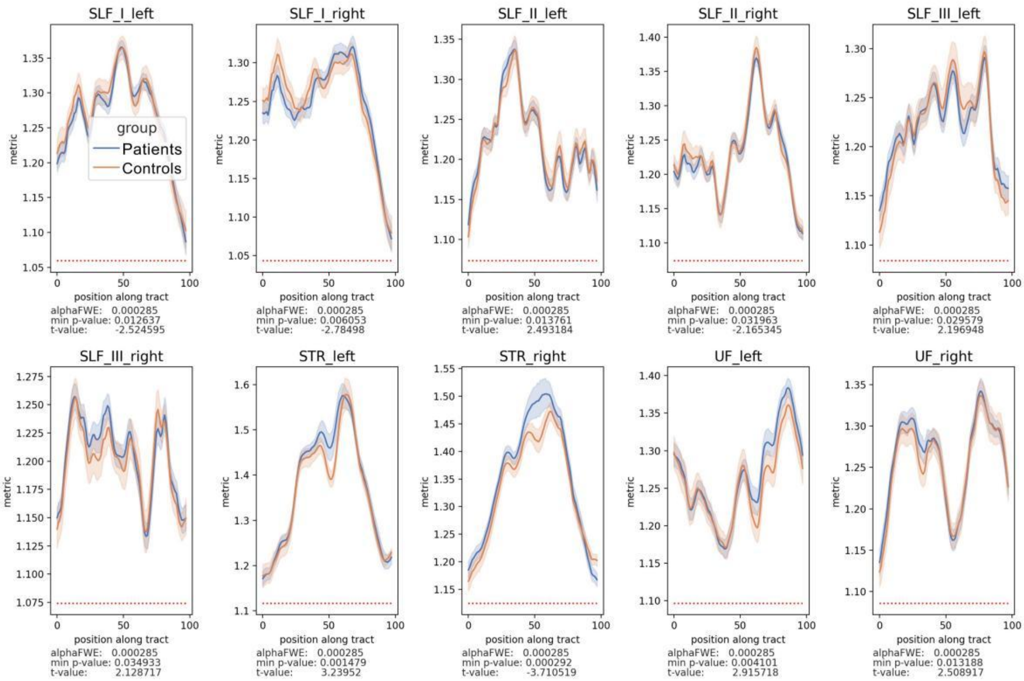
SLF_I_left
group
Patients
Controls
metric
position along tract
alphaFWE: 0.000285
min p-value: 0.012637
t-value: -2.524595
SLF_I_right
alphaFWE: 0.000285
min p-value: 0.006053
t-value: -2.78498
SLF_II_left
alphaFWE: 0.000285
min p-value: 0.013761
t-value: 2.493184
SLF_II_right
alphaFWE: 0.000285
min p-value: 0.031963
t-value: -2.165345
SLF_III_left
alphaFWE: 0.000285
min p-value: 0.029579
t-value: 2.196948
SLF_III_right
alphaFWE: 0.000285
min p-value: 0.034933
t-value: 2.128717
STR_left
alphaFWE: 0.000285
min p-value: 0.001479
t-value: 3.23952
STR_right
alphaFWE: 0.000285
min p-value: 0.000292
t-value: -3.710519
UF_left
alphaFWE: 0.000285
min p-value: 0.004101
t-value: 2.915718
UF_right
alphaFWE: 0.000285
min p-value: 0.013188
t-value: 2.508917

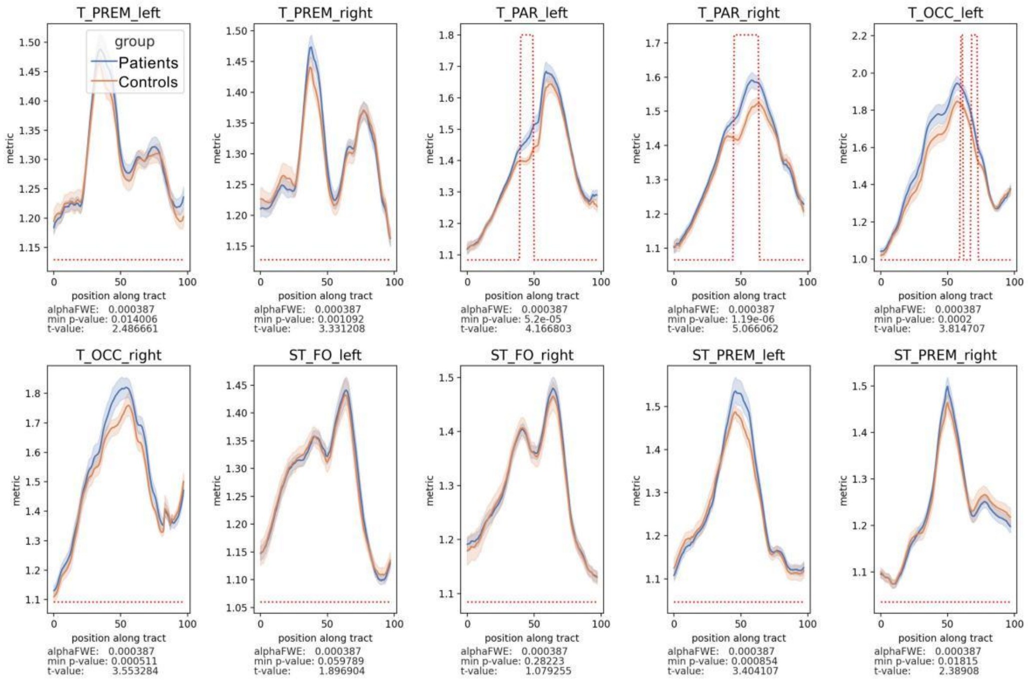

Supplementary Figure 3. Quantitative analysis of AD values of fifty white matter tracts in patients versus healthy controls.

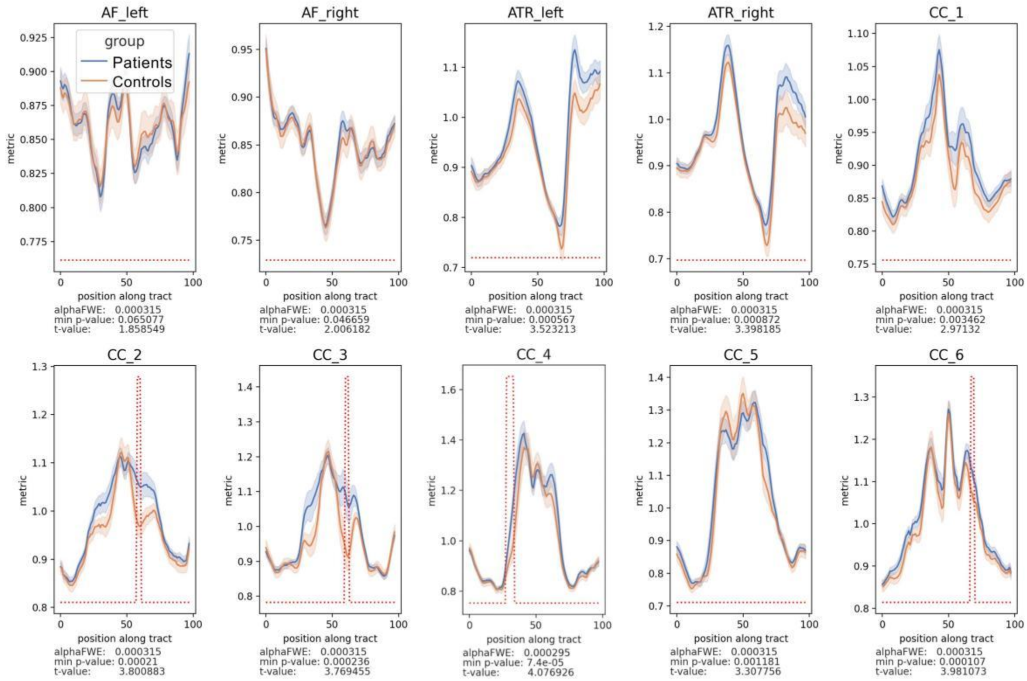
group
Patients
Controls
metric
position along tract
AF_left
alphaFWE: 0.000315
min p-value: 0.065077
t-value: 1.858549
AF_right
alphaFWE: 0.000315
min p-value: 0.046659
t-value: 2.006182
ATR_left
alphaFWE: 0.000315
min p-value: 0.000567
t-value: 3.523213
ATR_right
alphaFWE: 0.000315
min p-value: 0.000872
t-value: 3.398185
CC_1
alphaFWE: 0.000315
min p-value: 0.003462
t-value: 2.97132
CC_2
alphaFWE: 0.000315
min p-value: 0.00021
t-value: 3.800883
CC_3
alphaFWE: 0.000315
min p-value: 0.000236
t-value: 3.769455
CC_4
alphaFWE: 0.000295
min p-value: 7.4e-05
t-value: 4.076926
CC_5
alphaFWE: 0.000315
min p-value: 0.001181
t-value: 3.307756
CC_6
alphaFWE: 0.000315
min p-value: 0.000107
t-value: 3.981073

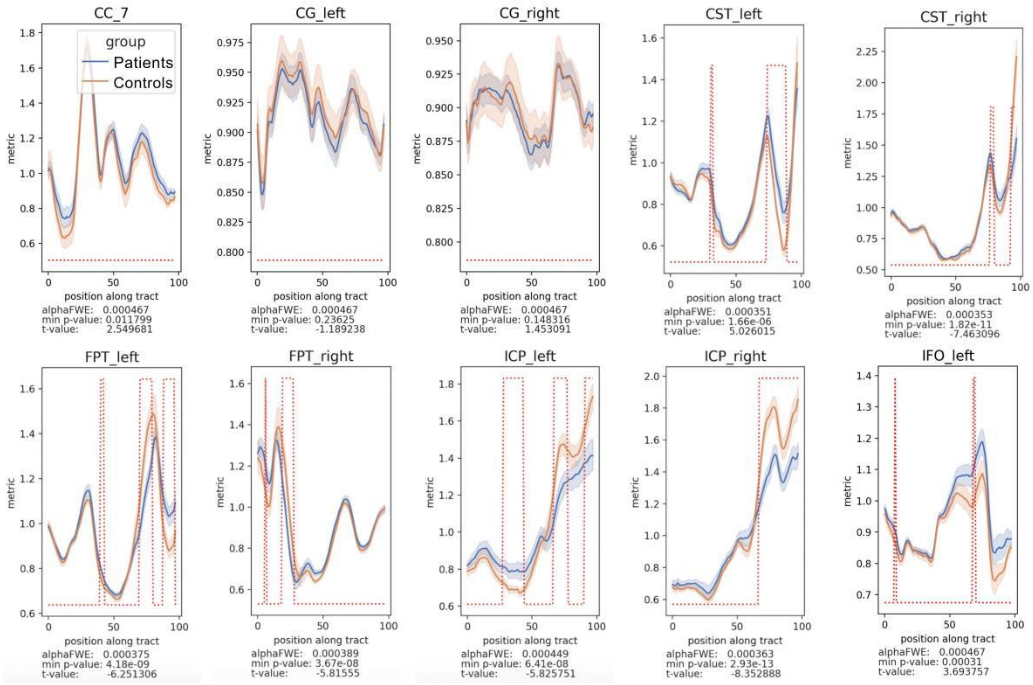
group
Patients
Controls
metric
position along tract
CC_7
alphaFWE: 0.000467
min p-value: 0.011799
t-value: 2.549681
CG_left
alphaFWE: 0.000467
min p-value: 0.23625
t-value: -1.189238
CG_right
alphaFWE: 0.000467
min p-value: 0.148316
t-value: 1.453091
CST_left
alphaFWE: 0.000351
min p-value: 1.66e-06
t-value: 5.026015
CST_right
alphaFWE: 0.000353
min p-value: 1.82e-11
t-value: -7.463096
FPT_left
alphaFWE: 0.000375
min p-value: 4.18e-09
t-value: -6.251306
FPT_right
alphaFWE: 0.000389
min p-value: 3.67e-08
t-value: -5.81555
ICP_left
alphaFWE: 0.000449
min p-value: 6.41e-08
t-value: -5.825751
ICP_right
alphaFWE: 0.000363
min p-value: 2.93e-13
t-value: -8.352888
IFO_left
alphaFWE: 0.000467
min p-value: 0.00031
t-value: 3.693757

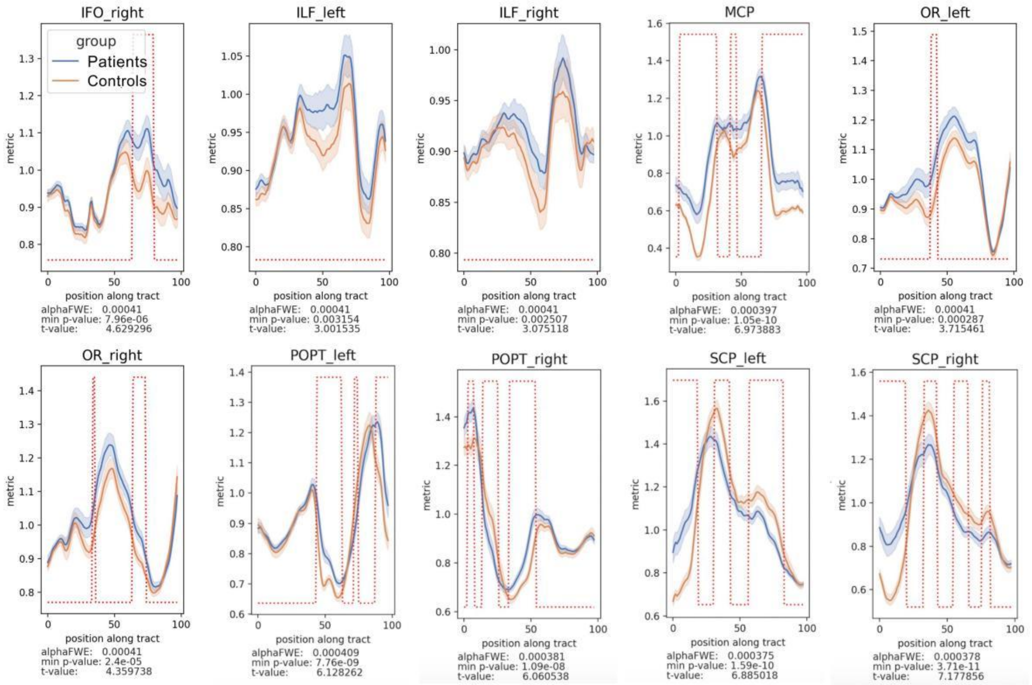
IFO_right
group
Patients
Controls
alphaFWE: 0.00041
min p-value: 7.96e-06
t-value: 4.629296
ILF_left
alphaFWE: 0.00041
min p-value: 0.003154
t-value: 3.001535
ILF_right
alphaFWE: 0.00041
min p-value: 0.002507
t-value: 3.075118
MCP
alphaFWE: 0.000397
min p-value: 1.05e-10
t-value: 6.973883
OR_left
alphaFWE: 0.00041
min p-value: 0.000287
t-value: 3.715461
OR_right
alphaFWE: 0.00041
min p-value: 2.4e-05
t-value: 4.359738
POPT_left
alphaFWE: 0.000409
min p-value: 7.76e-09
t-value: 6.128262
POPT_right
alphaFWE: 0.000381
min p-value: 1.09e-08
t-value: 6.060538
SCP_left
alphaFWE: 0.000375
min p-value: 1.59e-10
t-value: 6.885018
SCP_right
alphaFWE: 0.000378
min p-value: 3.71e-11
t-value: 7.177856
metric
position along tract

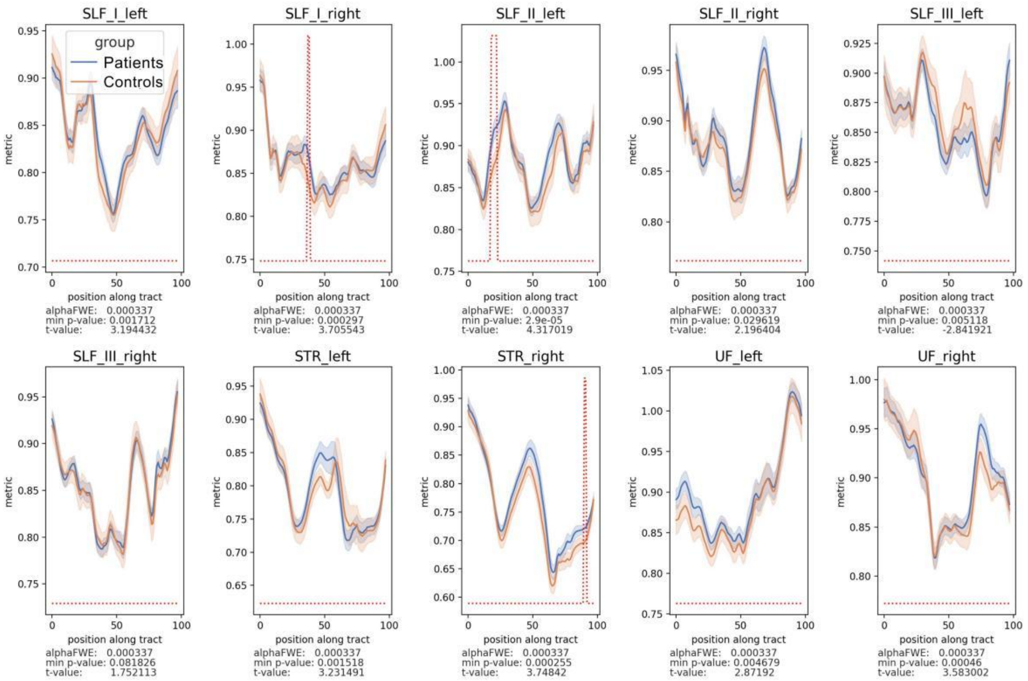
SLF_I_left
group
Patients
Controls
alphaFWE: 0.000337
min p-value: 0.001712
t-value: 3.194432
SLF_I_right
alphaFWE: 0.000337
min p-value: 0.000297
t-value: 3.705543
SLF_II_left
alphaFWE: 0.000337
min p-value: 2.9e-05
t-value: 4.317019
SLF_II_right
alphaFWE: 0.000337
min p-value: 0.029619
t-value: 2.196404
SLF_III_left
alphaFWE: 0.000337
min p-value: 0.005118
t-value: -2.841921
SLF_III_right
alphaFWE: 0.000337
min p-value: 0.081826
t-value: 1.752113
STR_left
alphaFWE: 0.000337
min p-value: 0.001518
t-value: 3.231491
STR_right
alphaFWE: 0.000337
min p-value: 0.000255
t-value: 3.74842
UF_left
alphaFWE: 0.000337
min p-value: 0.004679
t-value: 2.87192
UF_right
alphaFWE: 0.000337
min p-value: 0.00046
t-value: 3.583002
metric
position along tract

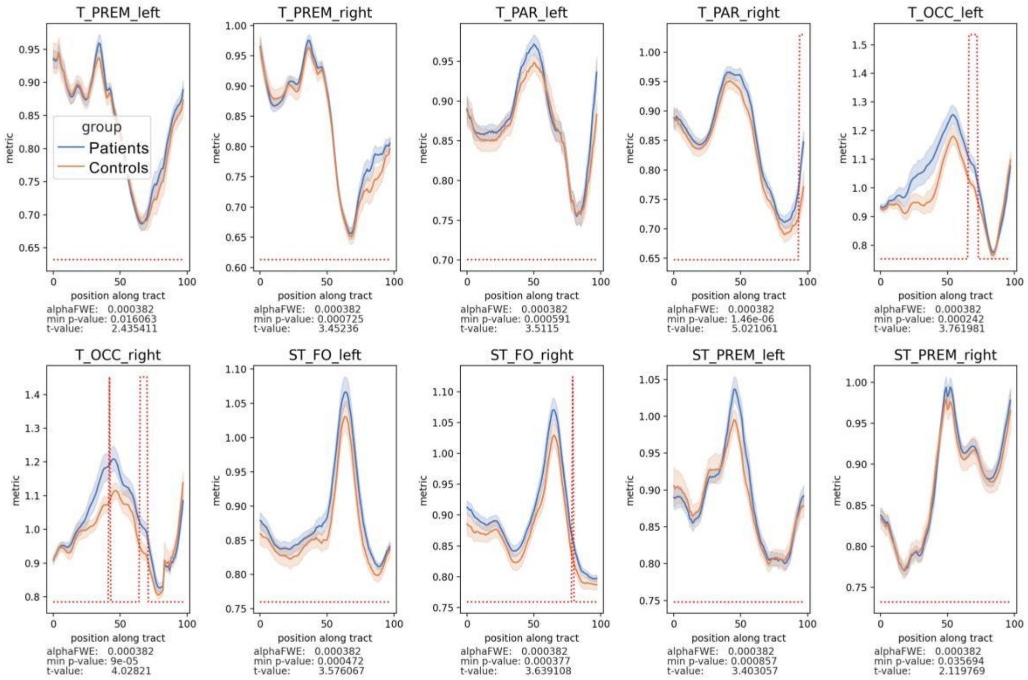

Supplementary Figure 4. Quantitative analysis of RD values of fifty white matter tracts in patients versus healthy controls.

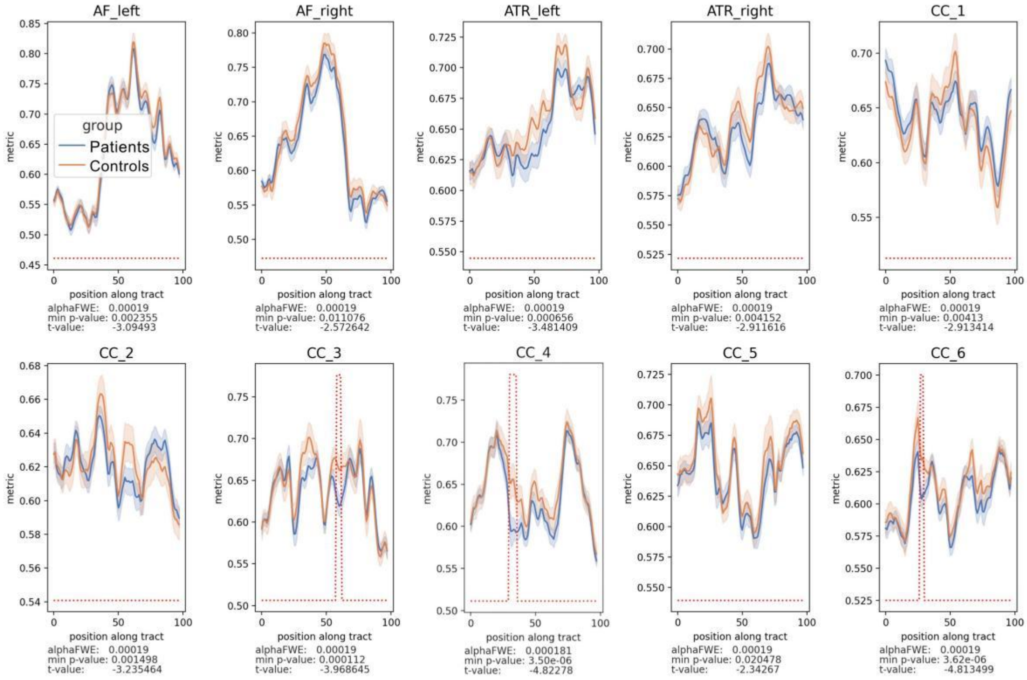
AF_left
alphaFWE: 0.00019
min p-value: 0.002355
t-value: -3.09493
AF_right
alphaFWE: 0.00019
min p-value: 0.011076
t-value: -2.572642
ATR_left
alphaFWE: 0.00019
min p-value: 0.000656
t-value: -3.481409
ATR_right
alphaFWE: 0.00019
min p-value: 0.004152
t-value: -2.911616
CC_1
alphaFWE: 0.00019
min p-value: 0.00413
t-value: -2.913414
CC_2
alphaFWE: 0.00019
min p-value: 0.001498
t-value: -3.235464
CC_3
alphaFWE: 0.00019
min p-value: 0.000112
t-value: -3.968645
CC_4
alphaFWE: 0.000181
min p-value: 3.50e-06
t-value: -4.82278
CC_5
alphaFWE: 0.00019
min p-value: 0.020478
t-value: -2.34267
CC_6
alphaFWE: 0.00019
min p-value: 3.62e-06
t-value: -4.813499
group
Patients
Controls
metric
position along tract

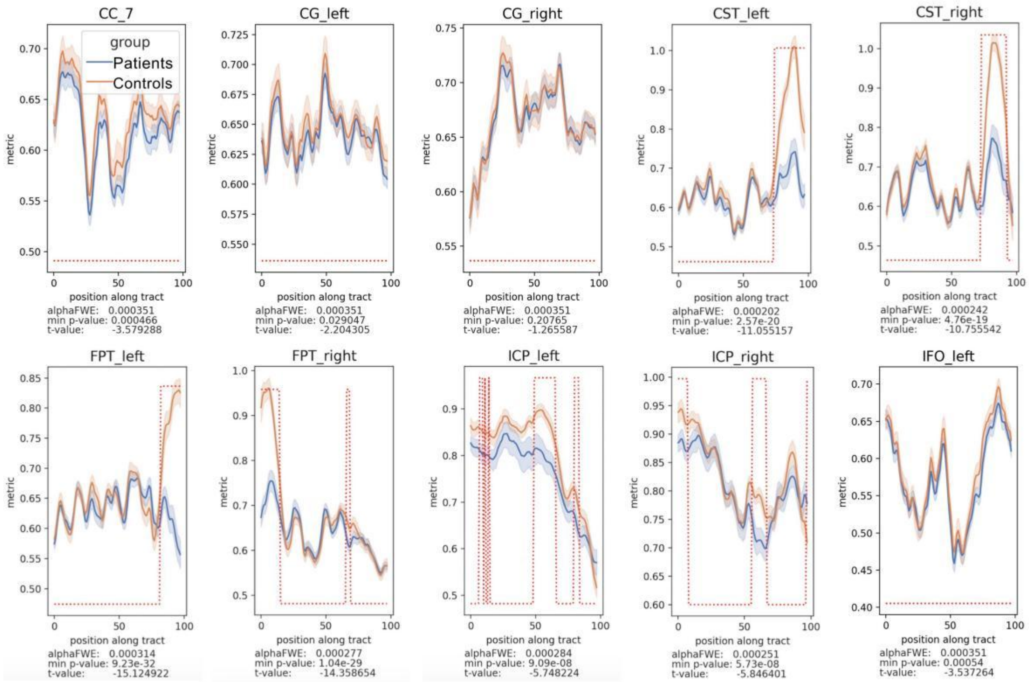
CC_7
alphaFWE: 0.000351
min p-value: 0.000466
t-value: -3.579288
CG_left
alphaFWE: 0.000351
min p-value: 0.029047
t-value: -2.204305
CG_right
alphaFWE: 0.000351
min p-value: 0.20765
t-value: -1.265587
CST_left
alphaFWE: 0.000202
min p-value: 2.57e-20
t-value: -11.055157
CST_right
alphaFWE: 0.000242
min p-value: 4.76e-19
t-value: -10.755542
FPT_left
alphaFWE: 0.000314
min p-value: 9.23e-32
t-value: -15.124922
FPT_right
alphaFWE: 0.000277
min p-value: 1.04e-29
t-value: -14.358654
ICP_left
alphaFWE: 0.000284
min p-value: 9.09e-08
t-value: -5.748224
ICP_right
alphaFWE: 0.000251
min p-value: 5.73e-08
t-value: -5.846401
IFO_left
alphaFWE: 0.000351
min p-value: 0.00054
t-value: -3.537264
group
Patients
Controls
metric
position along tract

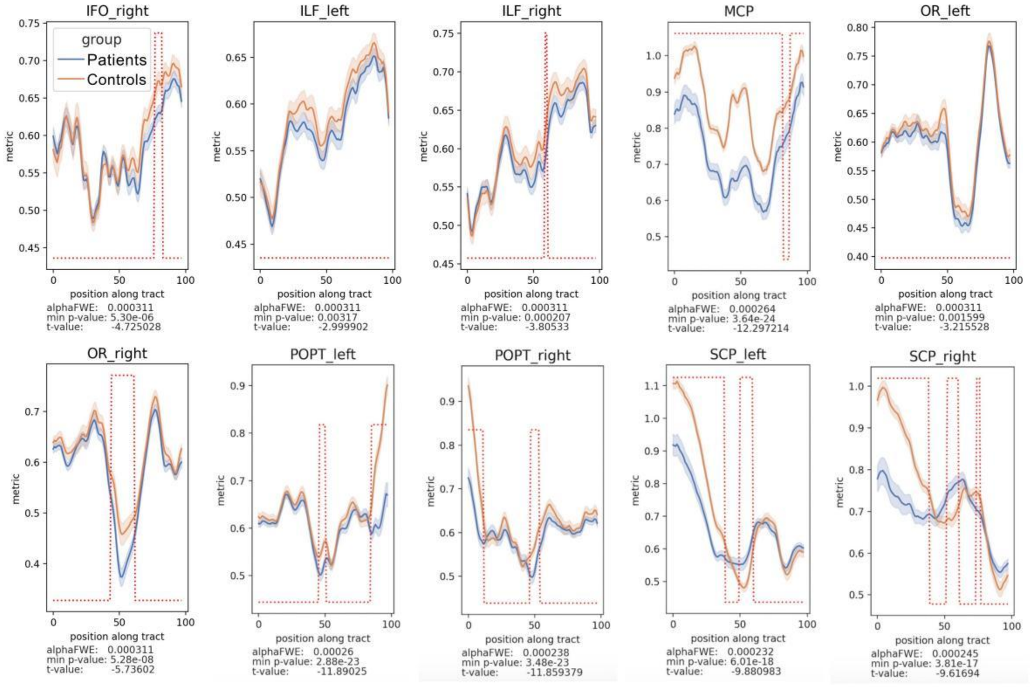
IFO_right
group
Patients
Controls
metric
position along tract
alphaFWE: 0.000311
min p-value: 5.30e-06
t-value: -4.725028
ILF_left
alphaFWE: 0.000311
min p-value: 0.00317
t-value: -2.999902
ILF_right
alphaFWE: 0.000311
min p-value: 0.000207
t-value: -3.80533
MCP
alphaFWE: 0.000264
min p-value: 3.64e-24
t-value: -12.297214
OR_left
alphaFWE: 0.000311
min p-value: 0.001599
t-value: -3.215528
OR_right
alphaFWE: 0.000311
min p-value: 5.28e-08
t-value: -5.73602
POPT_left
alphaFWE: 0.00026
min p-value: 2.88e-23
t-value: -11.89025
POPT_right
alphaFWE: 0.000238
min p-value: 3.48e-23
t-value: -11.859379
SCP_left
alphaFWE: 0.000232
min p-value: 6.01e-18
t-value: -9.880983
SCP_right
alphaFWE: 0.000245
min p-value: 3.81e-17
t-value: -9.61694

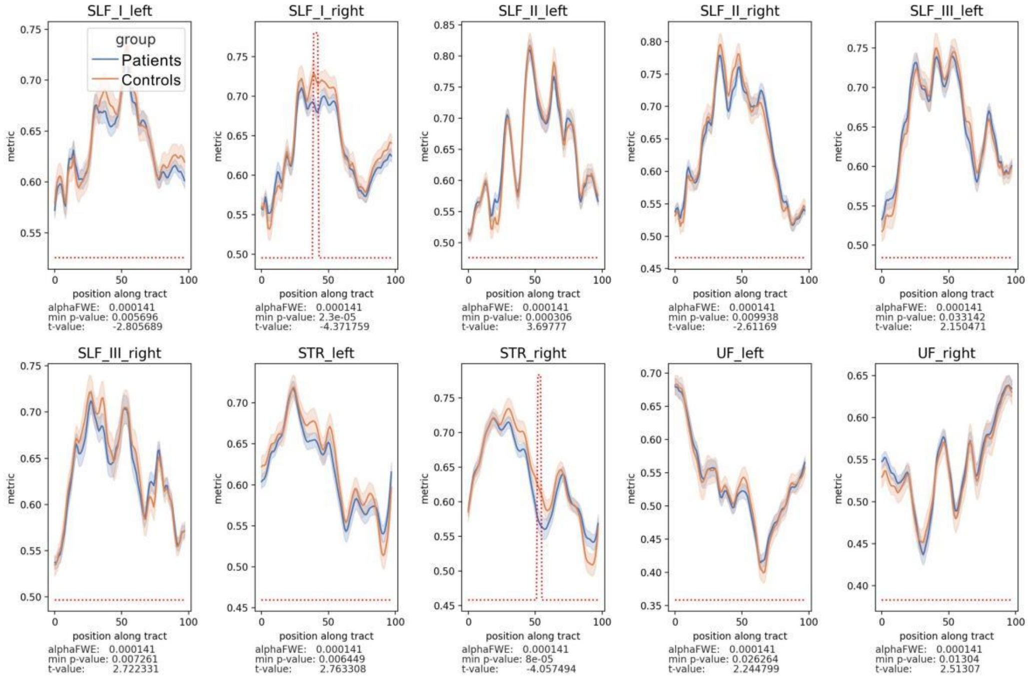
SLF_I_left
group
Patients
Controls
metric
position along tract
alphaFWE: 0.000141
min p-value: 0.005696
t-value: -2.805689
SLF_I_right
alphaFWE: 0.000141
min p-value: 2.3e-05
t-value: -4.371759
SLF_II_left
alphaFWE: 0.000141
min p-value: 0.000306
t-value: 3.69777
SLF_II_right
alphaFWE: 0.000141
min p-value: 0.009938
t-value: -2.61169
SLF_III_left
alphaFWE: 0.000141
min p-value: 0.033142
t-value: 2.150471
SLF_III_right
alphaFWE: 0.000141
min p-value: 0.007261
t-value: 2.722331
STR_left
alphaFWE: 0.000141
min p-value: 0.006449
t-value: 2.763308
STR_right
alphaFWE: 0.000141
min p-value: 8e-05
t-value: -4.057494
UF_left
alphaFWE: 0.000141
min p-value: 0.026264
t-value: 2.244799
UF_right
alphaFWE: 0.000141
min p-value: 0.01304
t-value: 2.51307

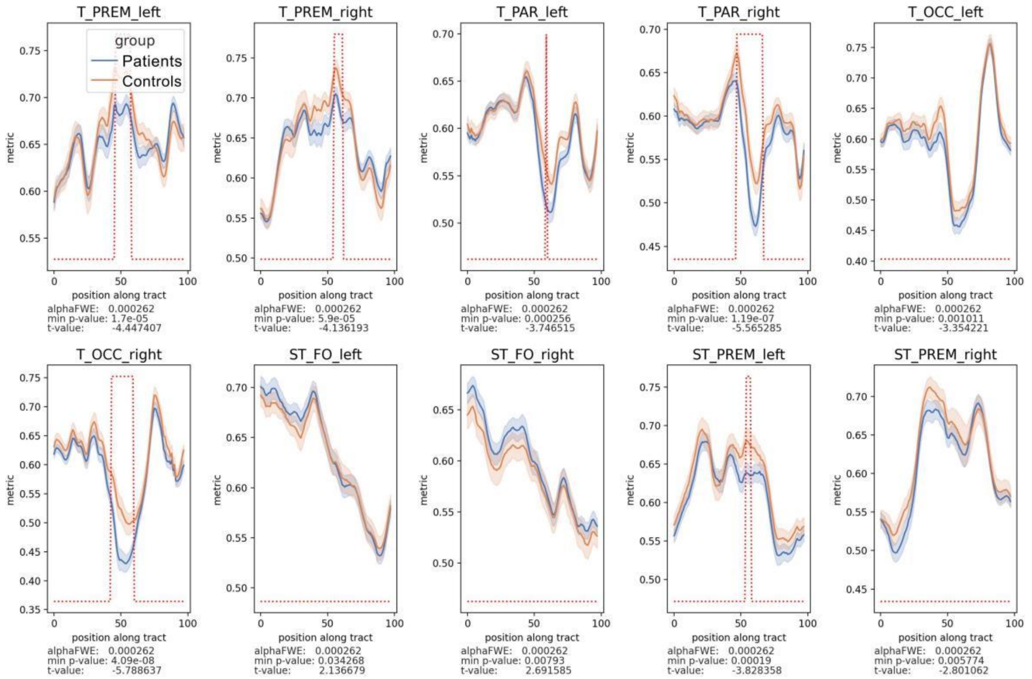

Supplementary Figure 5. Quantitative analysis of AK values of fifty white matter tracts in patients versus healthy controls.

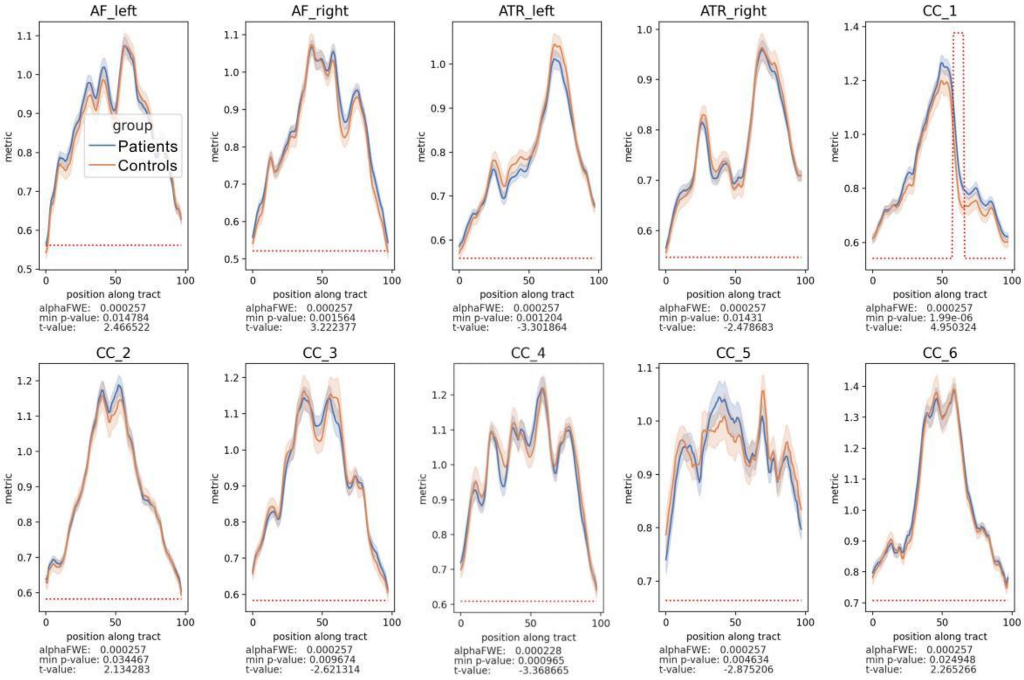

AF_left
AF_right
ATR_left
ATR_right
CC_1
CC_2
CC_3
CC_4
CC_5
CC_6
group
Patients
Controls
metric
position along tract
alphaFWE: 0.000257
min p-value: 0.014784
t-value: 2.466522
alphaFWE: 0.000257
min p-value: 0.001564
t-value: 3.222377
alphaFWE: 0.000257
min p-value: 0.001204
t-value: -3.301864
alphaFWE: 0.000257
min p-value: 0.01431
t-value: -2.478683
alphaFWE: 0.000257
min p-value: 1.99e-06
t-value: 4.950324
alphaFWE: 0.000257
min p-value: 0.034467
t-value: 2.134283
alphaFWE: 0.000257
min p-value: 0.009674
t-value: -2.621314
alphaFWE: 0.000228
min p-value: 0.000965
t-value: -3.368665
alphaFWE: 0.000257
min p-value: 0.004634
t-value: -2.875206
alphaFWE: 0.000257
min p-value: 0.024948
t-value: 2.265266

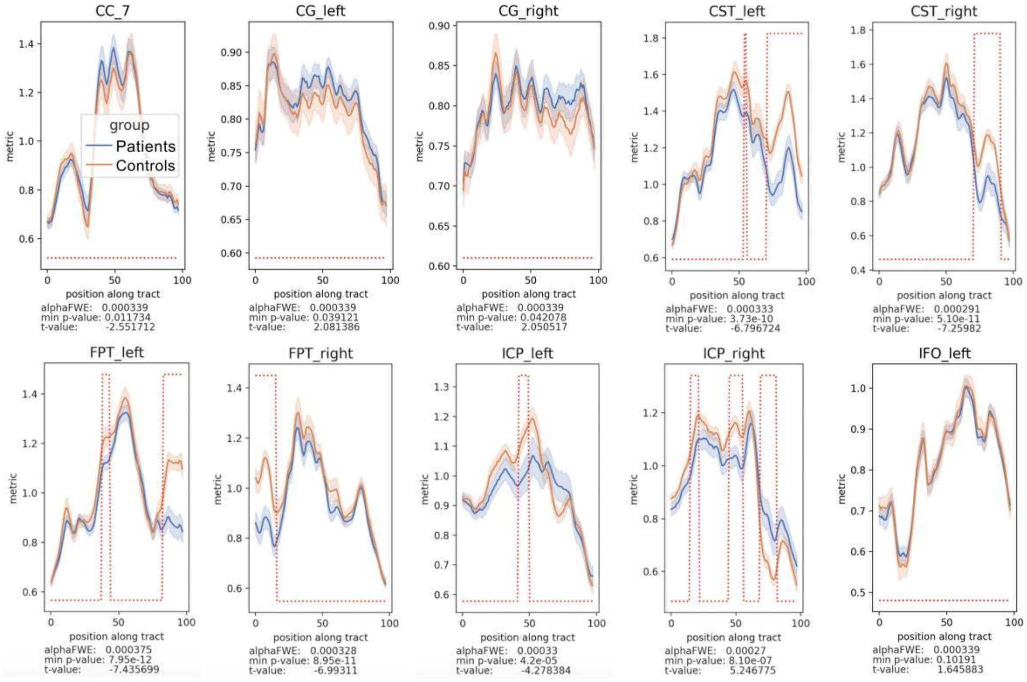

CC_7
CG_left
CG_right
CST_left
CST_right
FPT_left
FPT_right
ICP_left
ICP_right
IFO_left
group
Patients
Controls
metric
position along tract
alphaFWE: 0.000339
min p-value: 0.011734
t-value: -2.551712
alphaFWE: 0.000339
min p-value: 0.039121
t-value: 2.081386
alphaFWE: 0.000339
min p-value: 0.042078
t-value: 2.050517
alphaFWE: 0.000333
min p-value: 3.73e-10
t-value: -6.796724
alphaFWE: 0.000291
min p-value: 5.10e-11
t-value: -7.25982
alphaFWE: 0.000375
min p-value: 7.95e-12
t-value: -7.435699
alphaFWE: 0.000328
min p-value: 8.95e-11
t-value: -6.99311
alphaFWE: 0.00033
min p-value: 4.2e-05
t-value: -4.278384
alphaFWE: 0.00027
min p-value: 8.10e-07
t-value: 5.246775
alphaFWE: 0.000339
min p-value: 0.10191
t-value: 1.645883

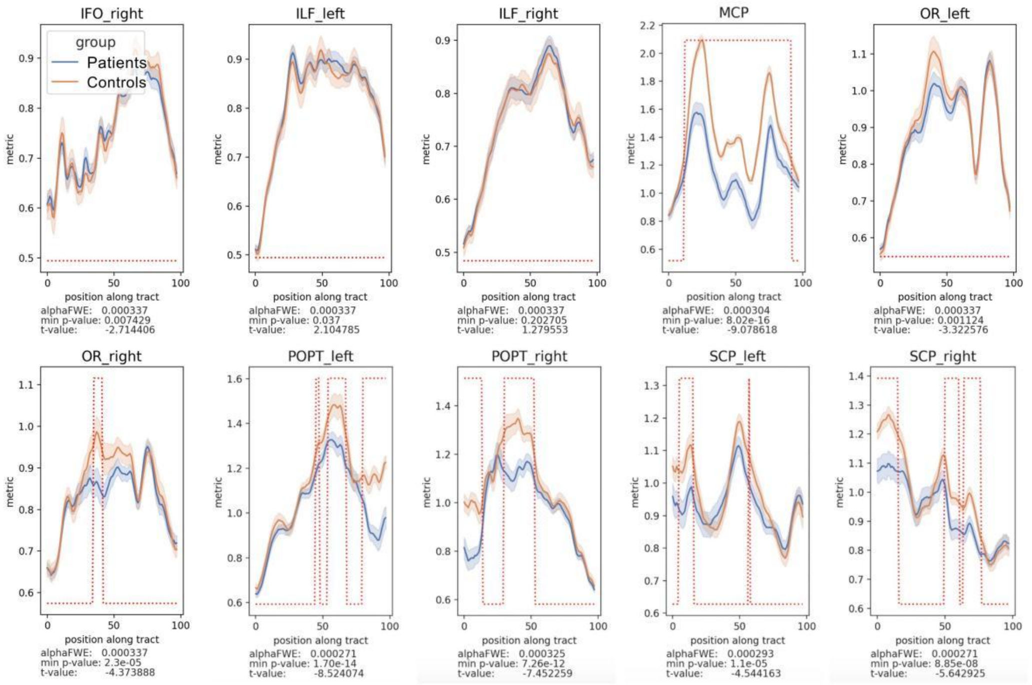
IFO_right
group
Patients
Controls
metric
position along tract
alphaFWE: 0.000337
min p-value: 0.007429
t-value: -2.714406
ILF_left
alphaFWE: 0.000337
min p-value: 0.037
t-value: 2.104785
ILF_right
alphaFWE: 0.000337
min p-value: 0.202705
t-value: 1.279553
MCP
alphaFWE: 0.000304
min p-value: 8.02e-16
t-value: -9.078618
OR_left
alphaFWE: 0.000337
min p-value: 0.001124
t-value: -3.322576
OR_right
alphaFWE: 0.000337
min p-value: 2.3e-05
t-value: -4.373888
POPT_left
alphaFWE: 0.000271
min p-value: 1.70e-14
t-value: -8.524074
POPT_right
alphaFWE: 0.000325
min p-value: 7.26e-12
t-value: -7.452259
SCP_left
alphaFWE: 0.000293
min p-value: 1.1e-05
t-value: -4.544163
SCP_right
alphaFWE: 0.000271
min p-value: 8.85e-08
t-value: -5.642925

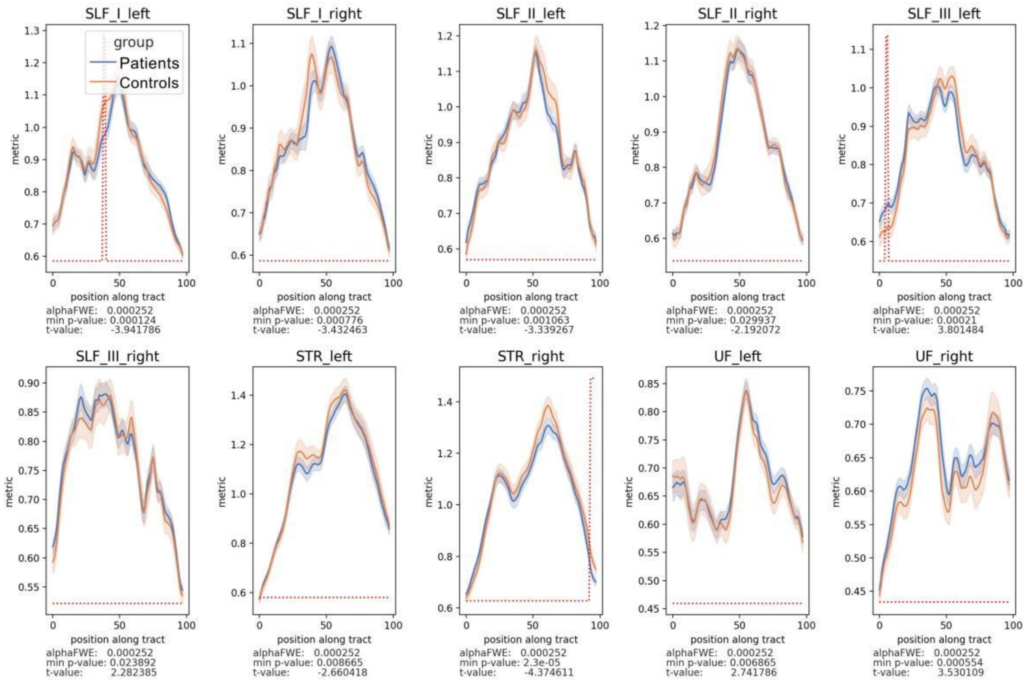
SLF_I_left
group
Patients
Controls
metric
position along tract
alphaFWE: 0.000252
min p-value: 0.000124
t-value: -3.941786
SLF_I_right
alphaFWE: 0.000252
min p-value: 0.000776
t-value: -3.432463
SLF_II_left
alphaFWE: 0.000252
min p-value: 0.001063
t-value: -3.339267
SLF_II_right
alphaFWE: 0.000252
min p-value: 0.029937
t-value: -2.192072
SLF_III_left
alphaFWE: 0.000252
min p-value: 0.00021
t-value: 3.801484
SLF_III_right
alphaFWE: 0.000252
min p-value: 0.023892
t-value: 2.282385
STR_left
alphaFWE: 0.000252
min p-value: 0.008665
t-value: -2.660418
STR_right
alphaFWE: 0.000252
min p-value: 2.3e-05
t-value: -4.374611
UF_left
alphaFWE: 0.000252
min p-value: 0.006865
t-value: 2.741786
UF_right
alphaFWE: 0.000252
min p-value: 0.000554
t-value: 3.530109

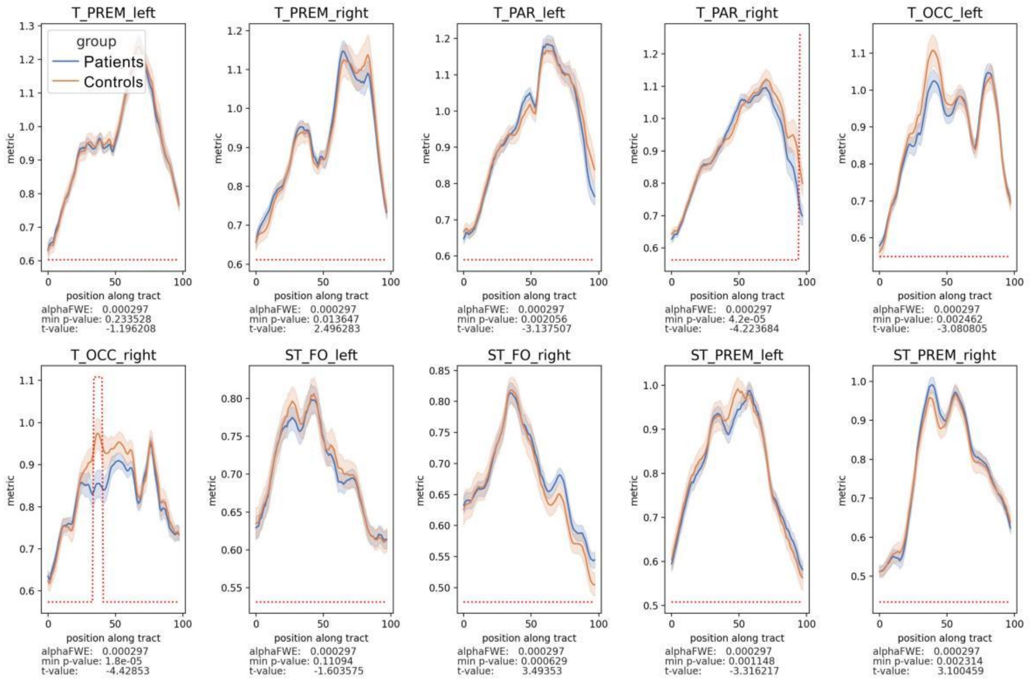

Supplementary Figure 6. Quantitative analysis of RK values of fifty white matter tracts in patients versus healthy controls.

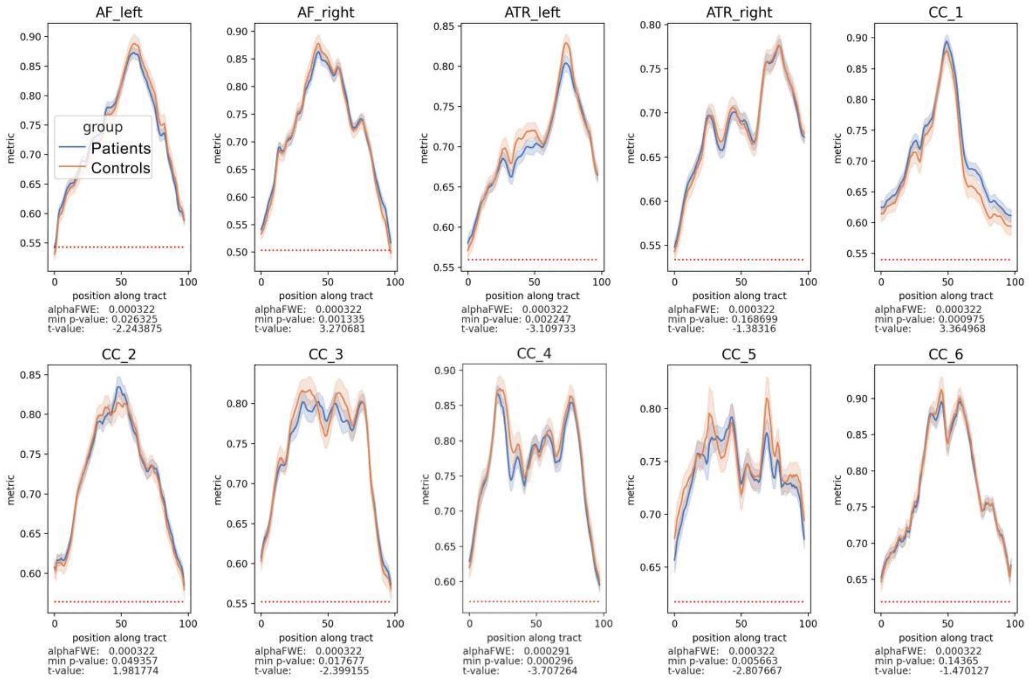
AF_left
group
Patients
Controls
metric
position along tract
alphaFWE: 0.000322
min p-value: 0.026325
t-value: -2.243875
AF_right
alphaFWE: 0.000322
min p-value: 0.001335
t-value: 3.270681
ATR_left
alphaFWE: 0.000322
min p-value: 0.002247
t-value: -3.109733
ATR_right
alphaFWE: 0.000322
min p-value: 0.168699
t-value: -1.38316
CC_1
alphaFWE: 0.000322
min p-value: 0.000975
t-value: 3.364968
CC_2
alphaFWE: 0.000322
min p-value: 0.049357
t-value: 1.981774
CC_3
alphaFWE: 0.000322
min p-value: 0.017677
t-value: -2.399155
CC_4
alphaFWE: 0.000291
min p-value: 0.000296
t-value: -3.707264
CC_5
alphaFWE: 0.000322
min p-value: 0.005663
t-value: -2.807667
CC_6
alphaFWE: 0.000322
min p-value: 0.14365
t-value: -1.470127

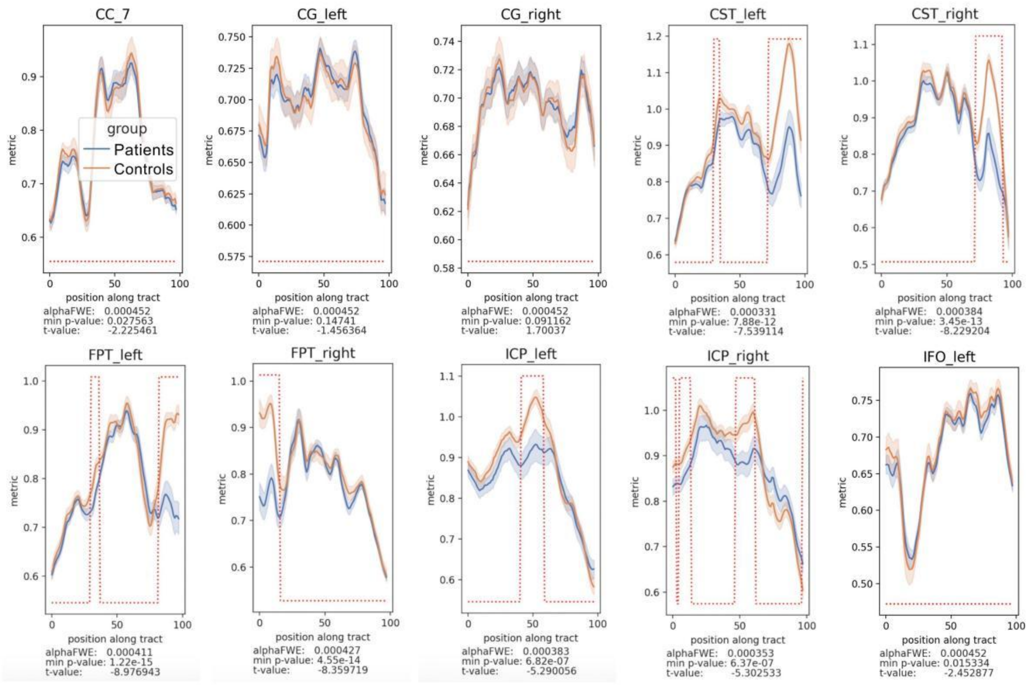
CC_7
group
Patients
Controls
metric
position along tract
alphaFWE: 0.000452
min p-value: 0.027563
t-value: -2.225461
CG_left
alphaFWE: 0.000452
min p-value: 0.14741
t-value: -1.456364
CG_right
alphaFWE: 0.000452
min p-value: 0.091162
t-value: 1.70037
CST_left
alphaFWE: 0.000331
min p-value: 7.88e-12
t-value: -7.539114
CST_right
alphaFWE: 0.000384
min p-value: 3.45e-13
t-value: -8.229204
FPT_left
alphaFWE: 0.000411
min p-value: 1.22e-15
t-value: -8.976943
FPT_right
alphaFWE: 0.000427
min p-value: 4.55e-14
t-value: -8.359719
ICP_left
alphaFWE: 0.000383
min p-value: 6.82e-07
t-value: -5.290056
ICP_right
alphaFWE: 0.000353
min p-value: 6.37e-07
t-value: -5.302533
IFO_left
alphaFWE: 0.000452
min p-value: 0.015334
t-value: -2.452877

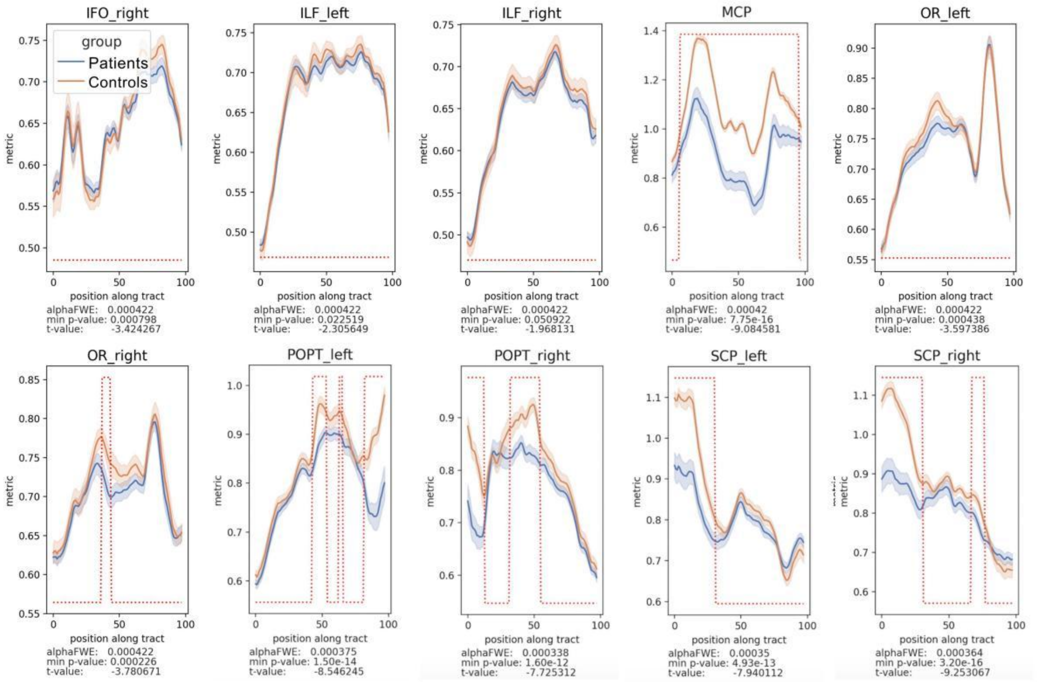
IFO_right
ILF_left
ILF_right
MCP
OR_left
group
Patients
Controls
metric
position along tract
alphaFWE: 0.000422
min p-value: 0.000798
t-value: -3.424267
alphaFWE: 0.000422
min p-value: 0.022519
t-value: -2.305649
alphaFWE: 0.000422
min p-value: 0.050922
t-value: -1.968131
alphaFWE: 0.00042
min p-value: 7.75e-16
t-value: -9.084581
alphaFWE: 0.000422
min p-value: 0.000438
t-value: -3.597386
OR_right
POPT_left
POPT_right
SCP_left
SCP_right
alphaFWE: 0.000422
min p-value: 0.000226
t-value: -3.780671
alphaFWE: 0.000375
min p-value: 1.50e-14
t-value: -8.546245
alphaFWE: 0.000338
min p-value: 1.60e-12
t-value: -7.725312
alphaFWE: 0.00035
min p-value: 4.93e-13
t-value: -7.940112
alphaFWE: 0.000364
min p-value: 3.20e-16
t-value: -9.253067

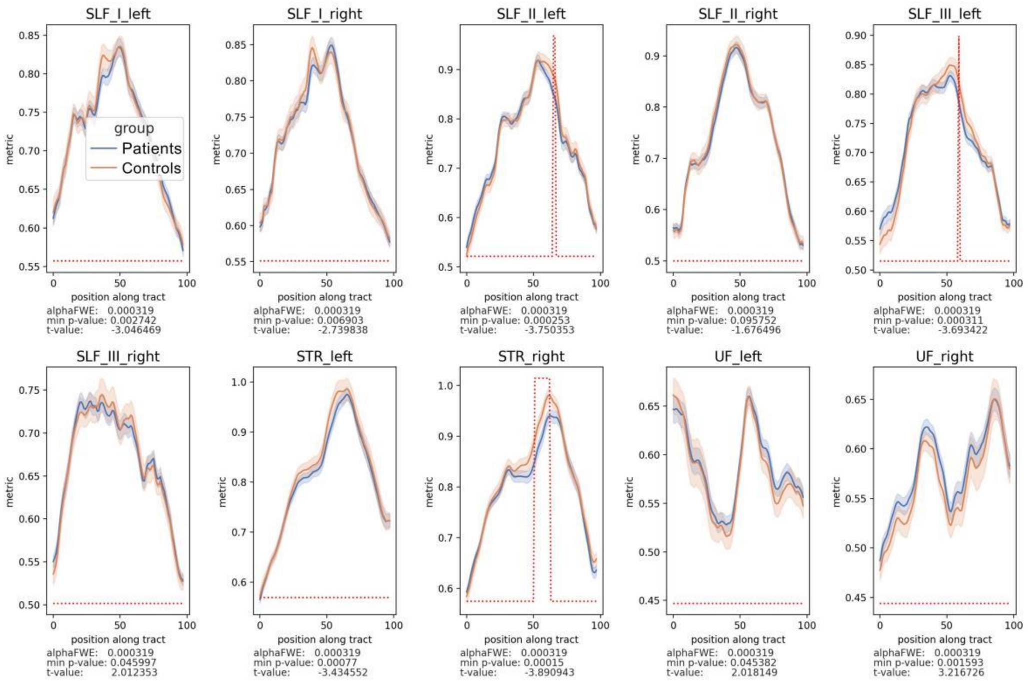
SLF_I_left
SLF_I_right
SLF_II_left
SLF_II_right
SLF_III_left
group
Patients
Controls
metric
position along tract
alphaFWE: 0.000319
min p-value: 0.002742
t-value: -3.046469
alphaFWE: 0.000319
min p-value: 0.006903
t-value: -2.739838
alphaFWE: 0.000319
min p-value: 0.000253
t-value: -3.750353
alphaFWE: 0.000319
min p-value: 0.095752
t-value: -1.676496
alphaFWE: 0.000319
min p-value: 0.000311
t-value: -3.693422
SLF_III_right
STR_left
STR_right
UF_left
UF_right
alphaFWE: 0.000319
min p-value: 0.045997
t-value: 2.012353
alphaFWE: 0.000319
min p-value: 0.00077
t-value: -3.434552
alphaFWE: 0.000319
min p-value: 0.00015
t-value: -3.890943
alphaFWE: 0.000319
min p-value: 0.045382
t-value: 2.018149
alphaFWE: 0.000319
min p-value: 0.001593
t-value: 3.216726

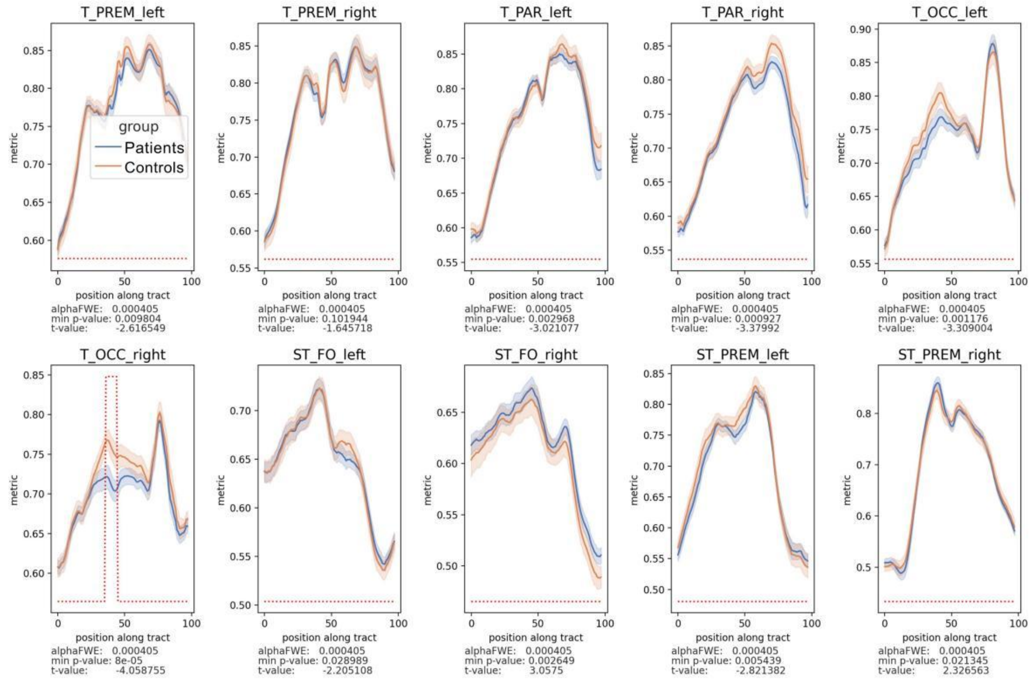

Supplementary Figure 7. Quantitative analysis of MK values of fifty white matter tracts in patients versus healthy controls.

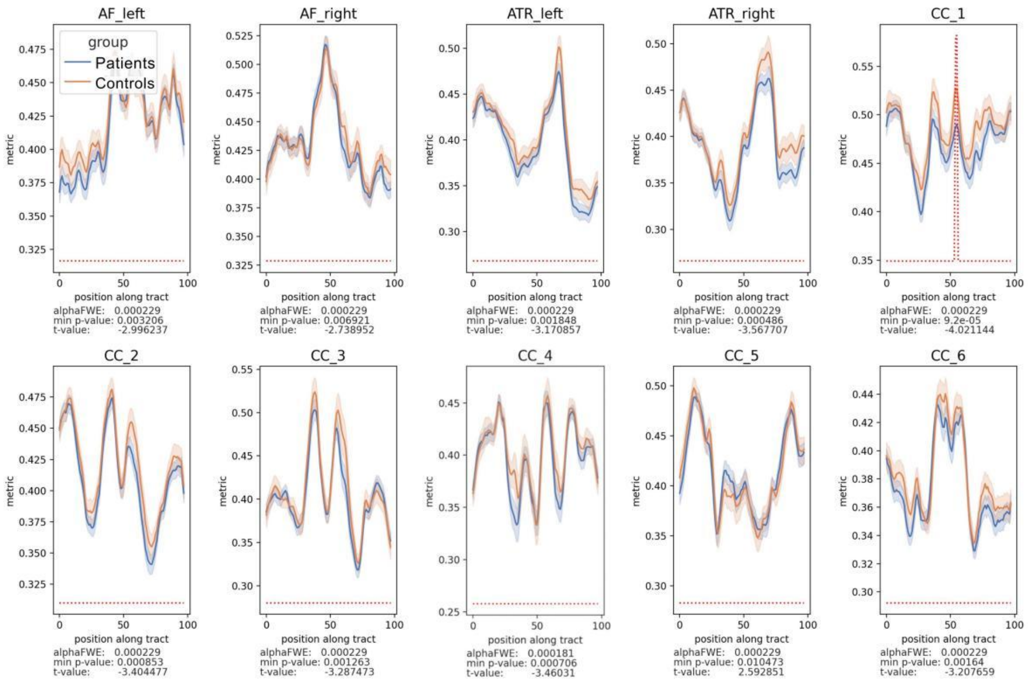
AF_left
group
Patients
Controls
metric
position along tract
alphaFWE: 0.000229
min p-value: 0.003206
t-value: -2.996237
AF_right
alphaFWE: 0.000229
min p-value: 0.006921
t-value: -2.738952
ATR_left
alphaFWE: 0.000229
min p-value: 0.001848
t-value: -3.170857
ATR_right
alphaFWE: 0.000229
min p-value: 0.000486
t-value: -3.567707
CC_1
alphaFWE: 0.000229
min p-value: 9.2e-05
t-value: -4.021144
CC_2
alphaFWE: 0.000229
min p-value: 0.000853
t-value: -3.404477
CC_3
alphaFWE: 0.000229
min p-value: 0.001263
t-value: -3.287473
CC_4
alphaFWE: 0.000181
min p-value: 0.000706
t-value: -3.46031
CC_5
alphaFWE: 0.000229
min p-value: 0.010473
t-value: 2.592851
CC_6
alphaFWE: 0.000229
min p-value: 0.00164
t-value: -3.207659

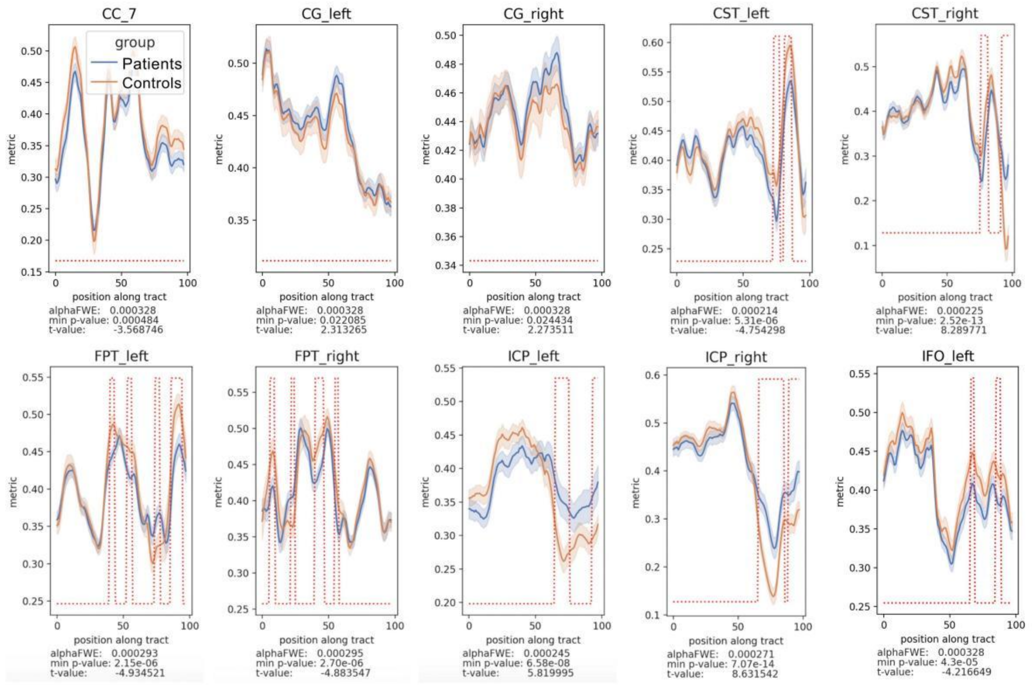
CC_7
group
Patients
Controls
metric
position along tract
alphaFWE: 0.000328
min p-value: 0.000484
t-value: -3.568746
CG_left
alphaFWE: 0.000328
min p-value: 0.022085
t-value: 2.313265
CG_right
alphaFWE: 0.000328
min p-value: 0.024434
t-value: 2.273511
CST_left
alphaFWE: 0.000214
min p-value: 5.31e-06
t-value: -4.754298
CST_right
alphaFWE: 0.000225
min p-value: 2.52e-13
t-value: 8.289771
FPT_left
alphaFWE: 0.000293
min p-value: 2.15e-06
t-value: -4.934521
FPT_right
alphaFWE: 0.000295
min p-value: 2.70e-06
t-value: -4.883547
ICP_left
alphaFWE: 0.000245
min p-value: 6.58e-08
t-value: 5.819995
ICP_right
alphaFWE: 0.000271
min p-value: 7.07e-14
t-value: 8.631542
IFO_left
alphaFWE: 0.000328
min p-value: 4.3e-05
t-value: -4.216649

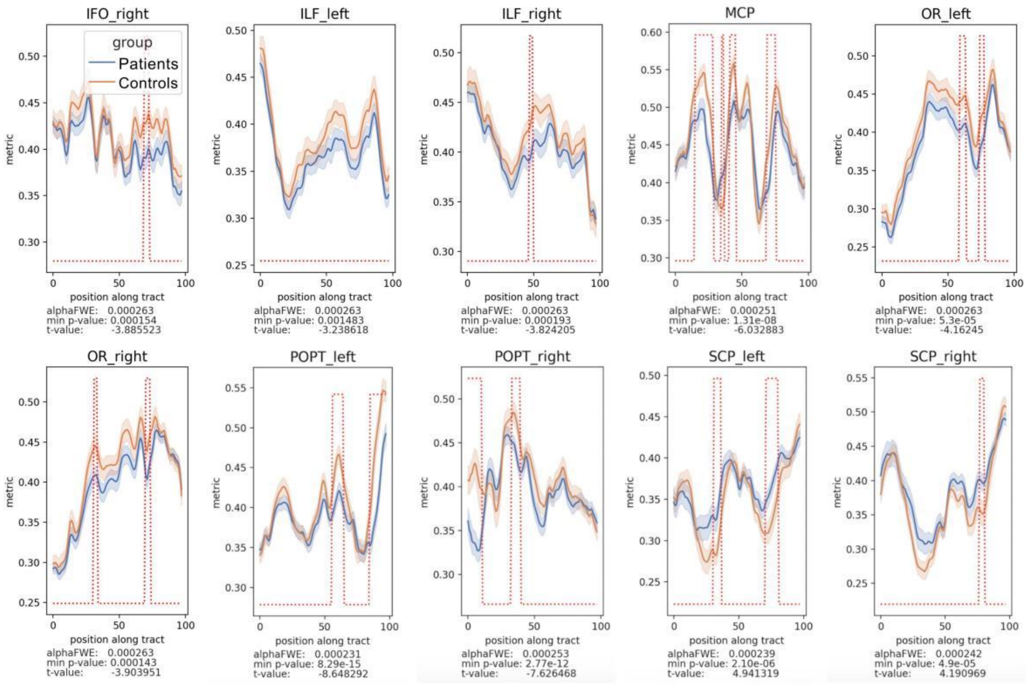
IFO_right
group
Patients
Controls
metric
position along tract
alphaFWE: 0.000263
min p-value: 0.000154
t-value: -3.885523
ILF_left
alphaFWE: 0.000263
min p-value: 0.001483
t-value: -3.238618
ILF_right
alphaFWE: 0.000263
min p-value: 0.000193
t-value: -3.824205
MCP
alphaFWE: 0.000251
min p-value: 1.31e-08
t-value: -6.032883
OR_left
alphaFWE: 0.000263
min p-value: 5.3e-05
t-value: -4.16245
OR_right
alphaFWE: 0.000263
min p-value: 0.000143
t-value: -3.903951
POPT_left
alphaFWE: 0.000231
min p-value: 8.29e-15
t-value: -8.648292
POPT_right
alphaFWE: 0.000253
min p-value: 2.77e-12
t-value: -7.626468
SCP_left
alphaFWE: 0.000239
min p-value: 2.10e-06
t-value: 4.941319
SCP_right
alphaFWE: 0.000242
min p-value: 4.9e-05
t-value: 4.190969

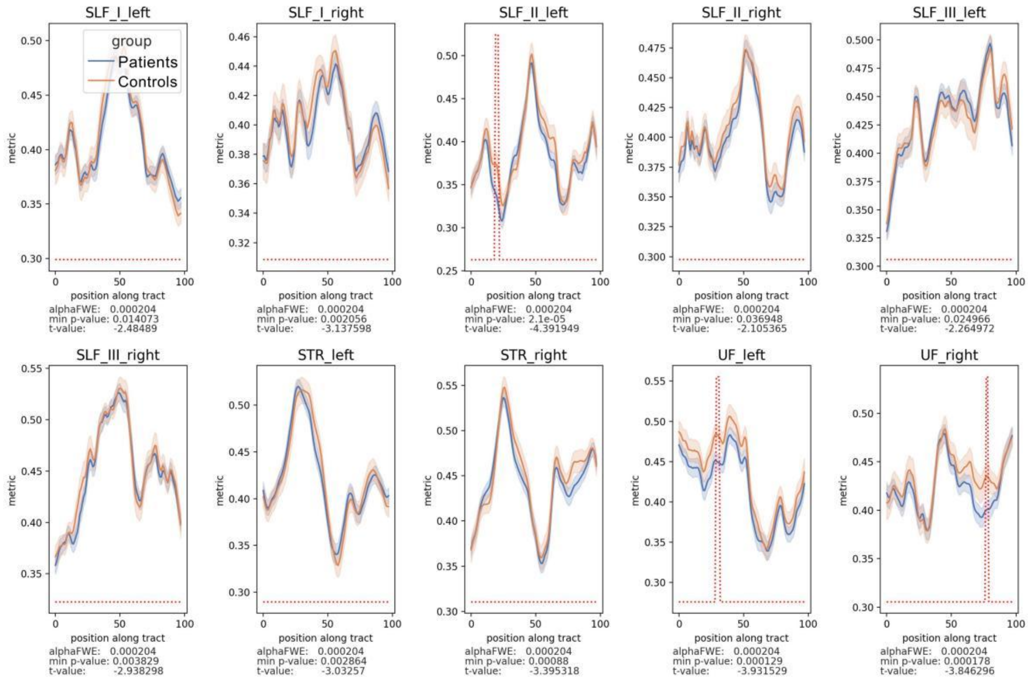
SLF_I_left
group
Patients
Controls
metric
position along tract
alphaFWE: 0.000204
min p-value: 0.014073
t-value: -2.48489
SLF_I_right
alphaFWE: 0.000204
min p-value: 0.002056
t-value: -3.137598
SLF_II_left
alphaFWE: 0.000204
min p-value: 2.1e-05
t-value: -4.391949
SLF_II_right
alphaFWE: 0.000204
min p-value: 0.036948
t-value: -2.105365
SLF_III_left
alphaFWE: 0.000204
min p-value: 0.024966
t-value: -2.264972
SLF_III_right
alphaFWE: 0.000204
min p-value: 0.003829
t-value: -2.938298
STR_left
alphaFWE: 0.000204
min p-value: 0.002864
t-value: -3.03257
STR_right
alphaFWE: 0.000204
min p-value: 0.00088
t-value: -3.395318
UF_left
alphaFWE: 0.000204
min p-value: 0.000129
t-value: -3.931529
UF_right
alphaFWE: 0.000204
min p-value: 0.000178
t-value: -3.846296

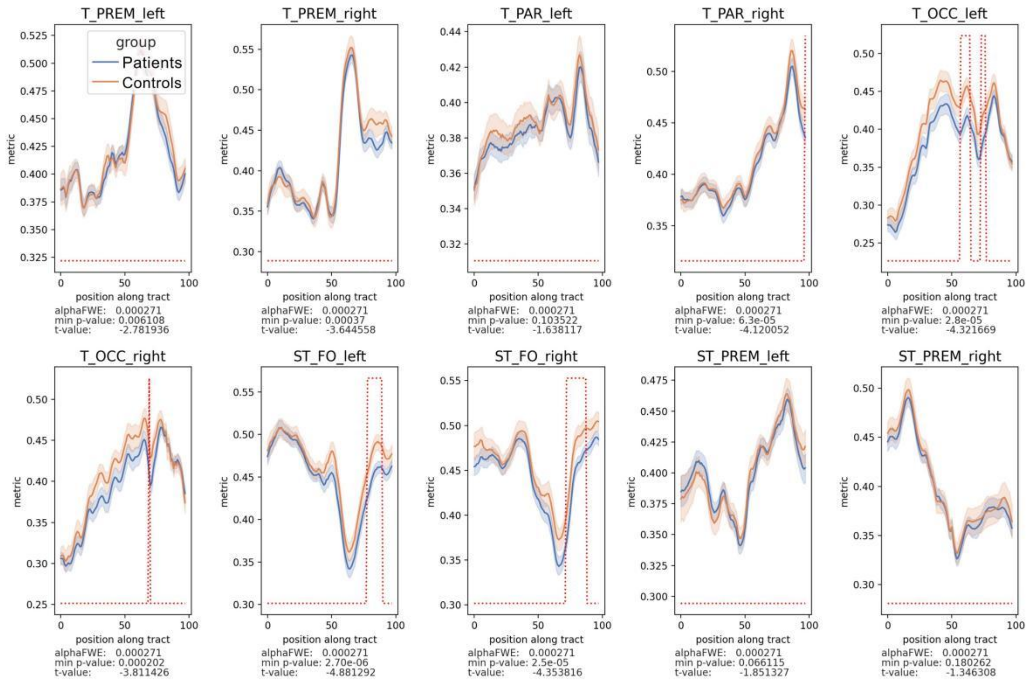

Supplementary Figure 8. Quantitative analysis of Fak values of fifty white matter tracts in patients versus healthy controls.

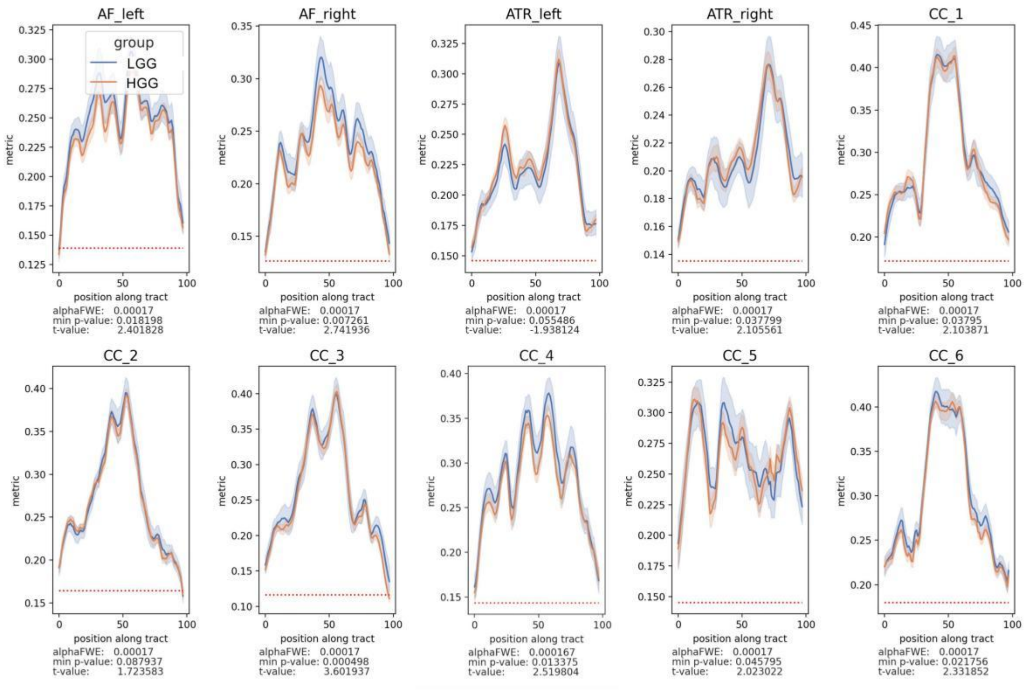
AF_left
group
LGG
HGG
metric
position along tract
alphaFWE: 0.00017
min p-value: 0.018198
t-value: 2.401828
AF_right
alphaFWE: 0.00017
min p-value: 0.007261
t-value: 2.741936
ATR_left
alphaFWE: 0.00017
min p-value: 0.055486
t-value: -1.938124
ATR_right
alphaFWE: 0.00017
min p-value: 0.037799
t-value: 2.105561
CC_1
alphaFWE: 0.00017
min p-value: 0.03795
t-value: 2.103871
CC_2
alphaFWE: 0.00017
min p-value: 0.087937
t-value: 1.723583
CC_3
alphaFWE: 0.00017
min p-value: 0.000498
t-value: 3.601937
CC_4
alphaFWE: 0.000167
min p-value: 0.013375
t-value: 2.519804
CC_5
alphaFWE: 0.00017
min p-value: 0.045795
t-value: 2.023022
CC_6
alphaFWE: 0.00017
min p-value: 0.021756
t-value: 2.331852

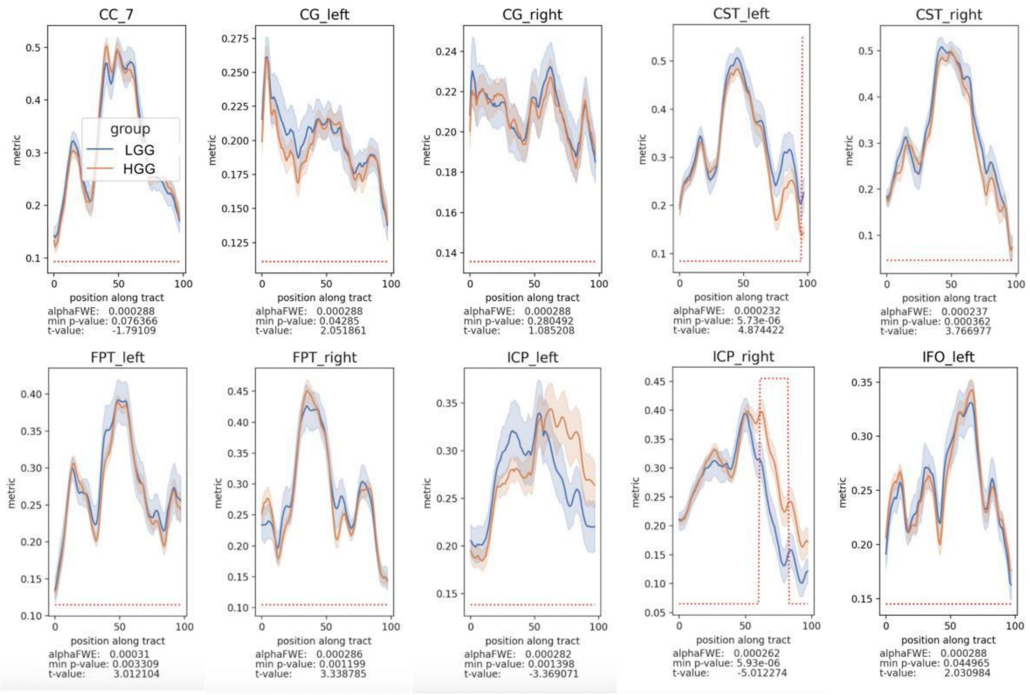
CC_7
group
LGG
HGG
metric
position along tract
alphaFWE: 0.000288
min p-value: 0.076366
t-value: -1.79109
CG_left
alphaFWE: 0.000288
min p-value: 0.04285
t-value: 2.051861
CG_right
alphaFWE: 0.000288
min p-value: 0.280492
t-value: 1.085208
CST_left
alphaFWE: 0.000232
min p-value: 5.73e-06
t-value: 4.874422
CST_right
alphaFWE: 0.000237
min p-value: 0.000362
t-value: 3.766977
FPT_left
alphaFWE: 0.00031
min p-value: 0.003309
t-value: 3.012104
FPT_right
alphaFWE: 0.000286
min p-value: 0.001199
t-value: 3.338785
ICP_left
alphaFWE: 0.000282
min p-value: 0.001398
t-value: -3.369071
ICP_right
alphaFWE: 0.000262
min p-value: 5.93e-06
t-value: -5.012274
IFO_left
alphaFWE: 0.000288
min p-value: 0.044965
t-value: 2.030984

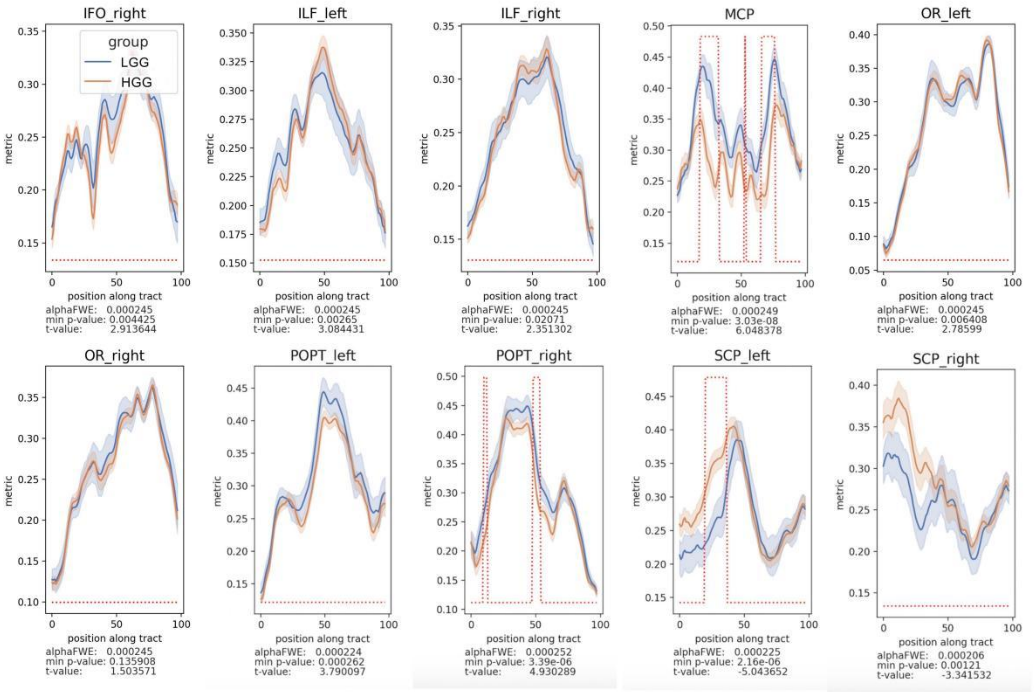
IFO_right
group
LGG
HGG
metric
position along tract
alphaFWE: 0.000245
min p-value: 0.004425
t-value: 2.913644
ILF_left
alphaFWE: 0.000245
min p-value: 0.00265
t-value: 3.084431
ILF_right
alphaFWE: 0.000245
min p-value: 0.02071
t-value: 2.351302
MCP
alphaFWE: 0.000249
min p-value: 3.03e-08
t-value: 6.048378
OR_left
alphaFWE: 0.000245
min p-value: 0.006408
t-value: 2.78599
OR_right
alphaFWE: 0.000245
min p-value: 0.135908
t-value: 1.503571
POPT_left
alphaFWE: 0.000224
min p-value: 0.000262
t-value: 3.790097
POPT_right
alphaFWE: 0.000252
min p-value: 3.39e-06
t-value: 4.930289
SCP_left
alphaFWE: 0.000225
min p-value: 2.16e-06
t-value: -5.043652
SCP_right
alphaFWE: 0.000206
min p-value: 0.00121
t-value: -3.341532

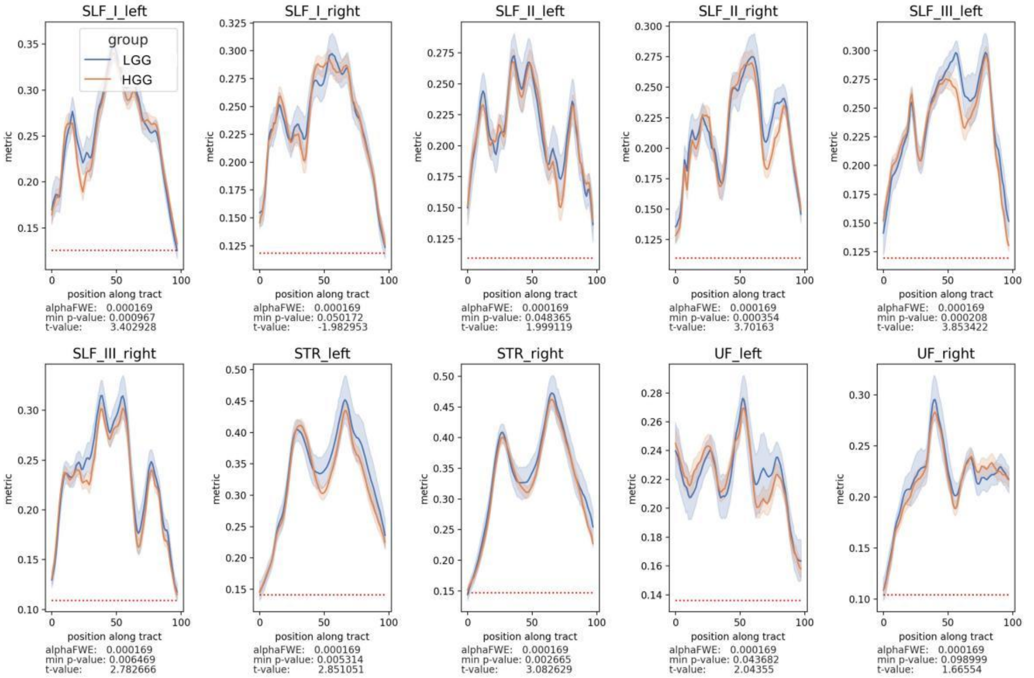
SLF_I_left
group
LGG
HGG
metric
position along tract
alphaFWE: 0.000169
min p-value: 0.000967
t-value: 3.402928
SLF_I_right
alphaFWE: 0.000169
min p-value: 0.050172
t-value: -1.982953
SLF_II_left
alphaFWE: 0.000169
min p-value: 0.048365
t-value: 1.999119
SLF_II_right
alphaFWE: 0.000169
min p-value: 0.000354
t-value: 3.70163
SLF_III_left
alphaFWE: 0.000169
min p-value: 0.000208
t-value: 3.853422
SLF_III_right
alphaFWE: 0.000169
min p-value: 0.006469
t-value: 2.782666
STR_left
alphaFWE: 0.000169
min p-value: 0.005314
t-value: 2.851051
STR_right
alphaFWE: 0.000169
min p-value: 0.002665
t-value: 3.082629
UF_left
alphaFWE: 0.000169
min p-value: 0.043682
t-value: 2.04355
UF_right
alphaFWE: 0.000169
min p-value: 0.098999
t-value: 1.66554

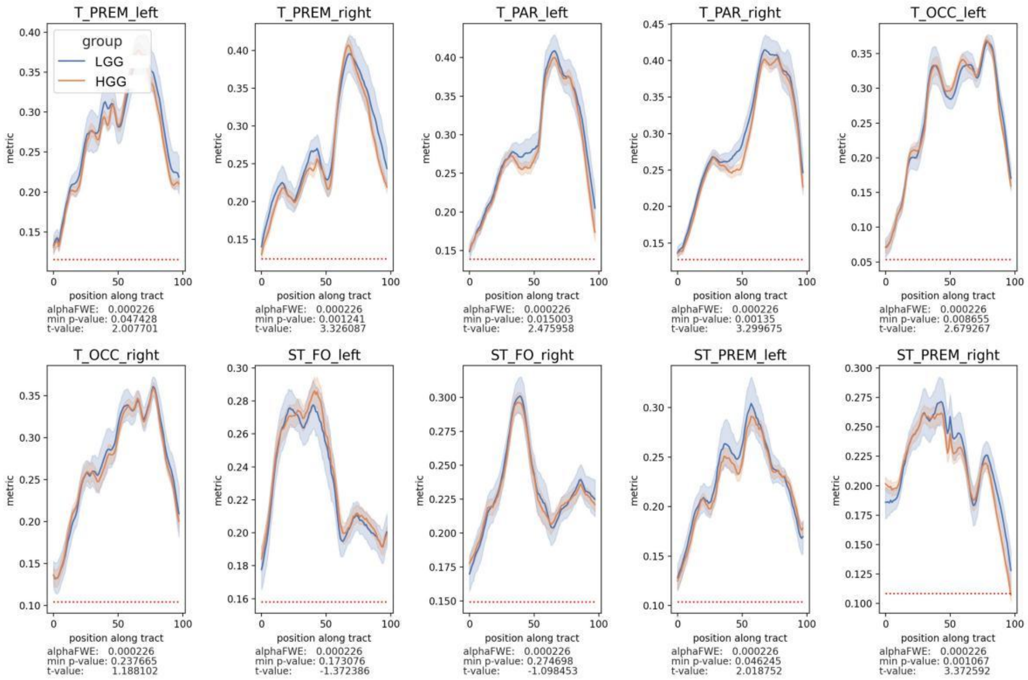

Supplementary Figure 9. Quantitative analysis of FA values of fifty white matter tracts in patients with LGG versus patients with HGG.

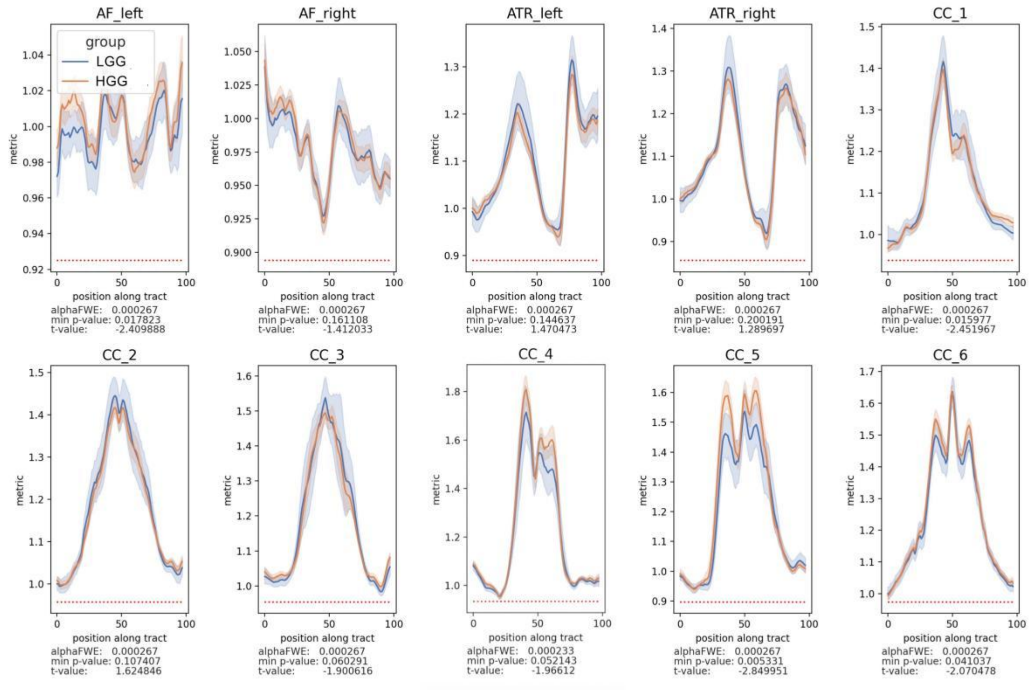
AF_left
group
LGG
HGG
alphaFWE: 0.000267
min p-value: 0.017823
t-value: -2.409888
AF_right
alphaFWE: 0.000267
min p-value: 0.161108
t-value: -1.412033
ATR_left
alphaFWE: 0.000267
min p-value: 0.144637
t-value: 1.470473
ATR_right
alphaFWE: 0.000267
min p-value: 0.200191
t-value: 1.289697
CC_1
alphaFWE: 0.000267
min p-value: 0.015977
t-value: -2.451967
CC_2
alphaFWE: 0.000267
min p-value: 0.107407
t-value: 1.624846
CC_3
alphaFWE: 0.000267
min p-value: 0.060291
t-value: -1.900616
CC_4
alphaFWE: 0.000233
min p-value: 0.052143
t-value: -1.96612
CC_5
alphaFWE: 0.000267
min p-value: 0.005331
t-value: -2.849951
CC_6
alphaFWE: 0.000267
min p-value: 0.041037
t-value: -2.070478
metric
position along tract

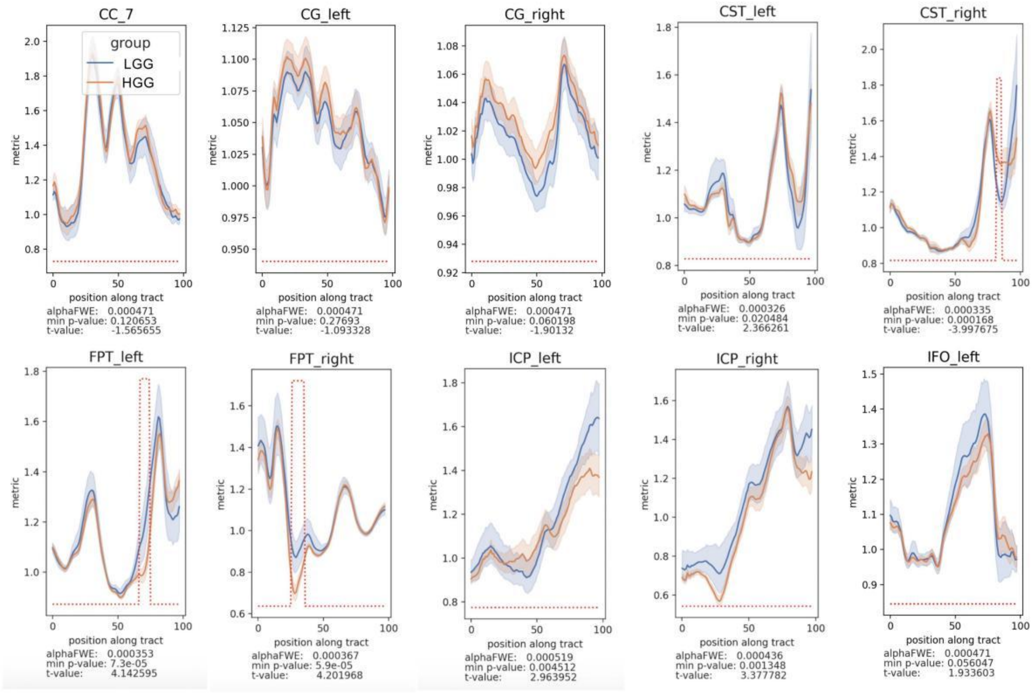
CC_7
group
LGG
HGG
alphaFWE: 0.000471
min p-value: 0.120653
t-value: -1.565655
CG_left
alphaFWE: 0.000471
min p-value: 0.27693
t-value: -1.093328
CG_right
alphaFWE: 0.000471
min p-value: 0.060198
t-value: -1.90132
CST_left
alphaFWE: 0.000326
min p-value: 0.020484
t-value: 2.366261
CST_right
alphaFWE: 0.000335
min p-value: 0.000168
t-value: -3.997675
FPT_left
alphaFWE: 0.000353
min p-value: 7.3e-05
t-value: 4.142595
FPT_right
alphaFWE: 0.000367
min p-value: 5.9e-05
t-value: 4.201968
ICP_left
alphaFWE: 0.000519
min p-value: 0.004512
t-value: 2.963952
ICP_right
alphaFWE: 0.000436
min p-value: 0.001348
t-value: 3.377782
IFO_left
alphaFWE: 0.000471
min p-value: 0.056047
t-value: 1.933603
metric
position along tract

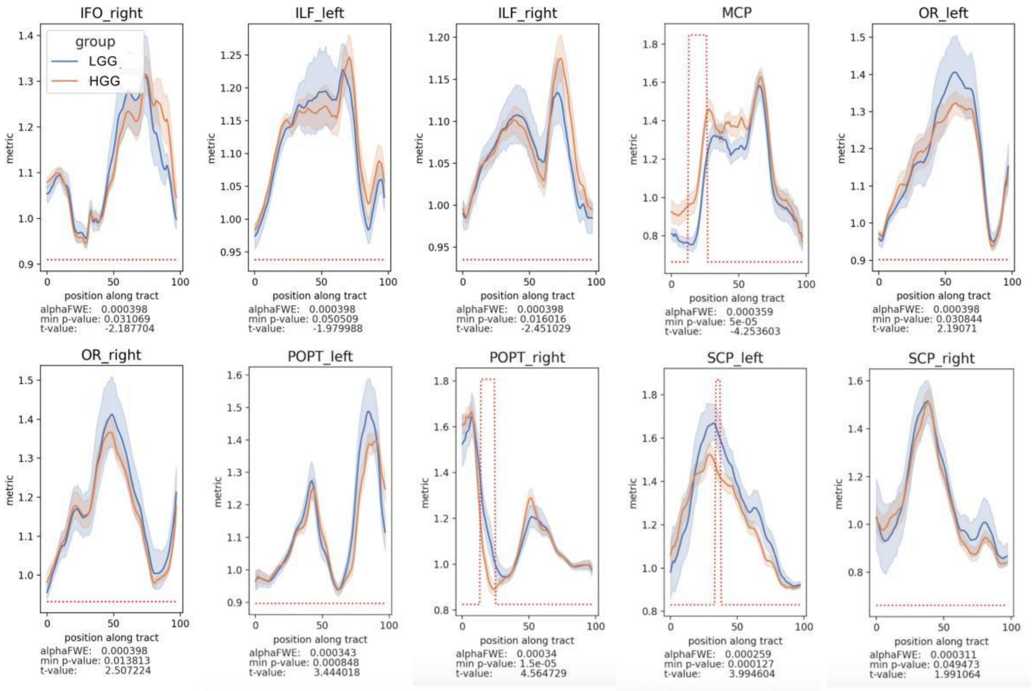
IFO_right
ILF_left
ILF_right
MCP
OR_left
group
LGG
HGG
metric
position along tract
alphaFWE: 0.000398
min p-value: 0.031069
t-value: -2.187704
alphaFWE: 0.000398
min p-value: 0.050509
t-value: -1.979988
alphaFWE: 0.000398
min p-value: 0.016016
t-value: -2.451029
alphaFWE: 0.000359
min p-value: 5e-05
t-value: -4.253603
alphaFWE: 0.000398
min p-value: 0.030844
t-value: 2.19071
OR_right
POPT_left
POPT_right
SCP_left
SCP_right
alphaFWE: 0.000398
min p-value: 0.013813
t-value: 2.507224
alphaFWE: 0.000343
min p-value: 0.000848
t-value: 3.444018
alphaFWE: 0.00034
min p-value: 1.5e-05
t-value: 4.564729
alphaFWE: 0.000259
min p-value: 0.000127
t-value: 3.994604
alphaFWE: 0.000311
min p-value: 0.049473
t-value: 1.991064

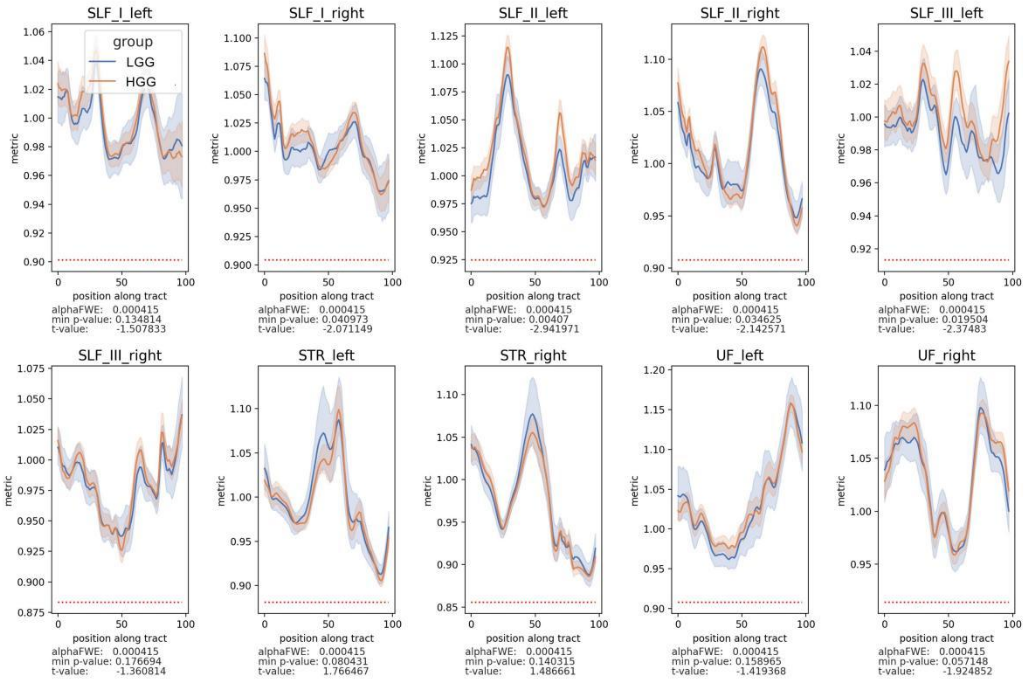
SLF_I_left
SLF_I_right
SLF_II_left
SLF_II_right
SLF_III_left
group
LGG
HGG
metric
position along tract
alphaFWE: 0.000415
min p-value: 0.134814
t-value: -1.507833
alphaFWE: 0.000415
min p-value: 0.040973
t-value: -2.071149
alphaFWE: 0.000415
min p-value: 0.00407
t-value: -2.941971
alphaFWE: 0.000415
min p-value: 0.034625
t-value: -2.142571
alphaFWE: 0.000415
min p-value: 0.019504
t-value: -2.37483
SLF_III_right
STR_left
STR_right
UF_left
UF_right
alphaFWE: 0.000415
min p-value: 0.176694
t-value: -1.360814
alphaFWE: 0.000415
min p-value: 0.080431
t-value: 1.766467
alphaFWE: 0.000415
min p-value: 0.140315
t-value: 1.486661
alphaFWE: 0.000415
min p-value: 0.158965
t-value: -1.419368
alphaFWE: 0.000415
min p-value: 0.057148
t-value: -1.924852

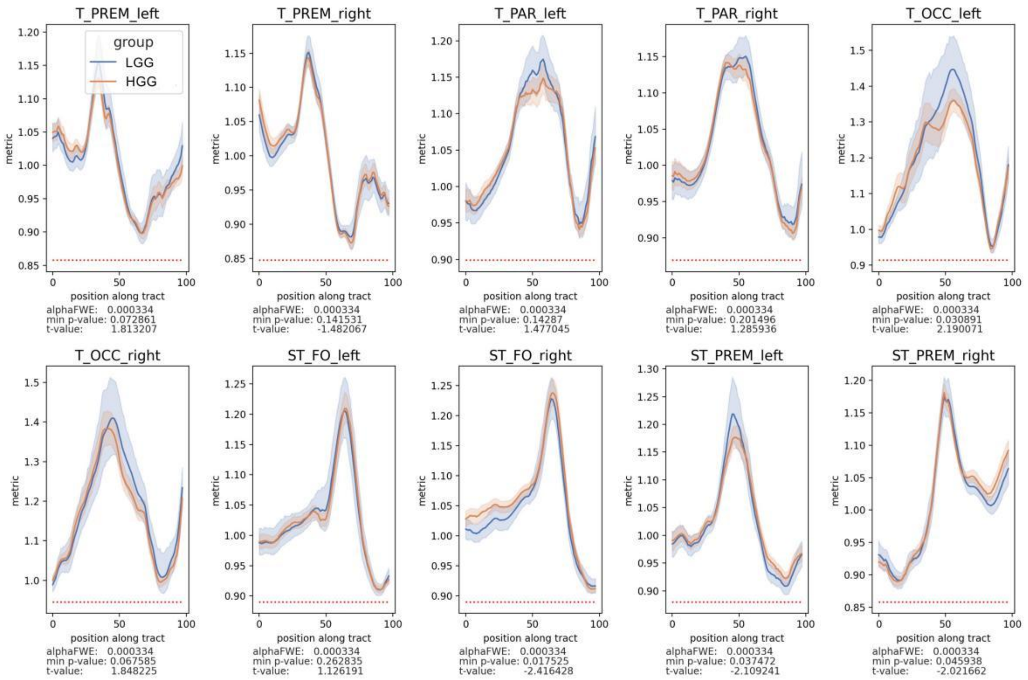

Supplementary Figure 10. Quantitative analysis of MD values of fifty white matter tracts in patients with LGG versus patients with HGG.

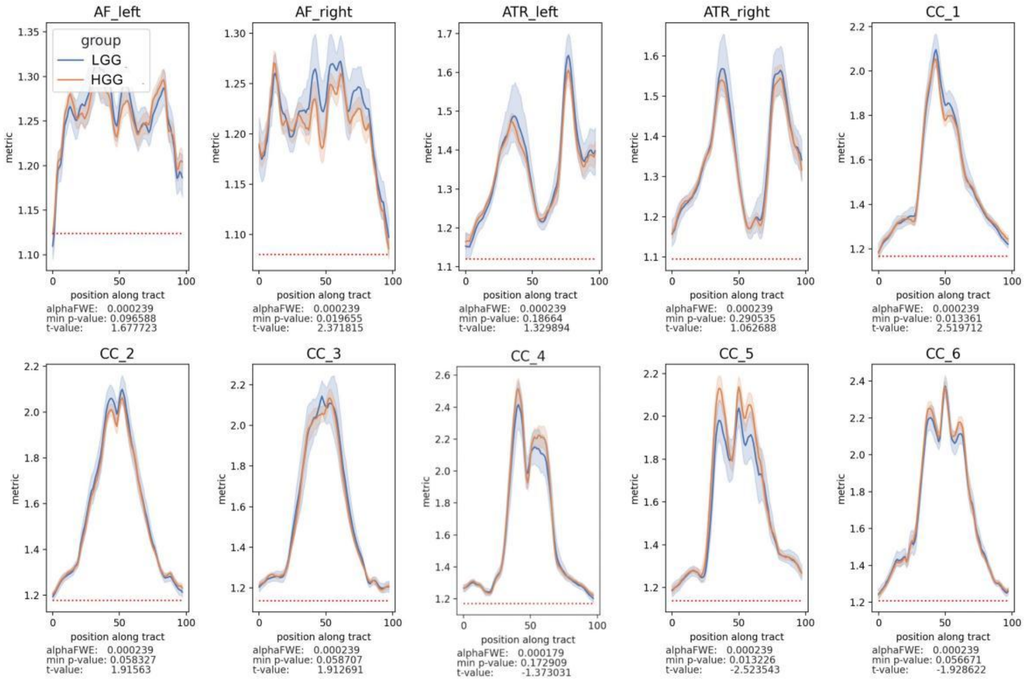
AF_left
group
LGG
HGG
metric
position along tract
alphaFWE: 0.000239
min p-value: 0.096588
t-value: 1.677723
AF_right
alphaFWE: 0.000239
min p-value: 0.019655
t-value: 2.371815
ATR_left
alphaFWE: 0.000239
min p-value: 0.18664
t-value: 1.329894
ATR_right
alphaFWE: 0.000239
min p-value: 0.290535
t-value: 1.062688
CC_1
alphaFWE: 0.000239
min p-value: 0.013361
t-value: 2.519712
CC_2
alphaFWE: 0.000239
min p-value: 0.058327
t-value: 1.91563
CC_3
alphaFWE: 0.000239
min p-value: 0.058707
t-value: 1.912691
CC_4
alphaFWE: 0.000179
min p-value: 0.172909
t-value: -1.373031
CC_5
alphaFWE: 0.000239
min p-value: 0.013226
t-value: -2.523543
CC_6
alphaFWE: 0.000239
min p-value: 0.056671
t-value: -1.928622

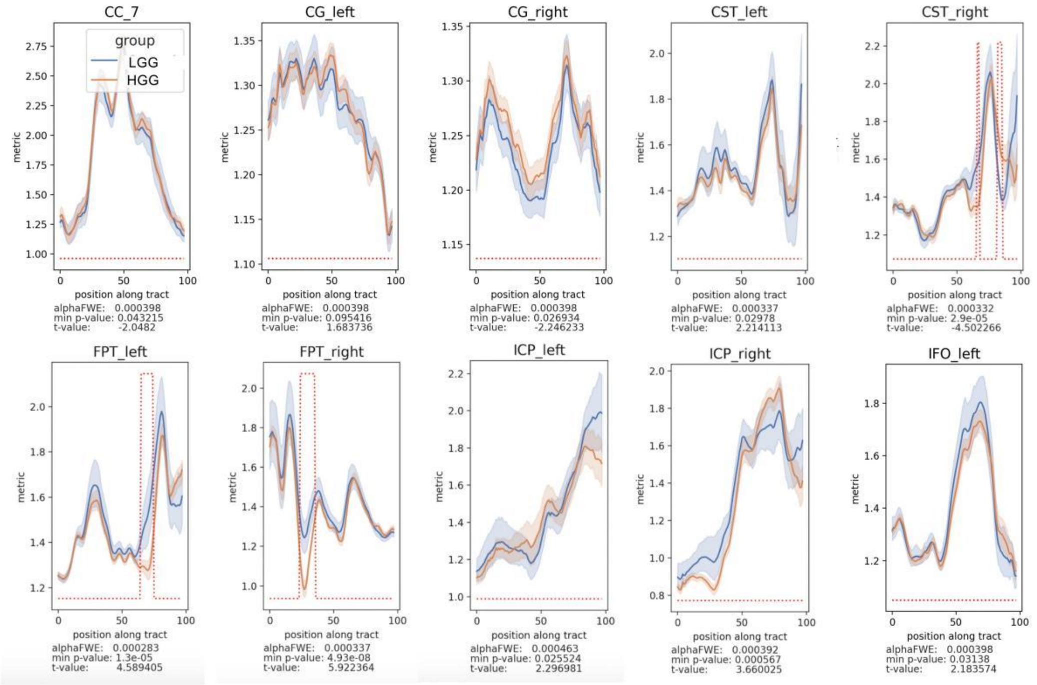
CC_7
group
LGG
HGG
metric
position along tract
alphaFWE: 0.000398
min p-value: 0.043215
t-value: -2.0482
CG_left
alphaFWE: 0.000398
min p-value: 0.095416
t-value: 1.683736
CG_right
alphaFWE: 0.000398
min p-value: 0.026934
t-value: -2.246233
CST_left
alphaFWE: 0.000337
min p-value: 0.02978
t-value: 2.214113
CST_right
alphaFWE: 0.000332
min p-value: 2.9e-05
t-value: -4.502266
FPT_left
alphaFWE: 0.000283
min p-value: 1.3e-05
t-value: 4.589405
FPT_right
alphaFWE: 0.000337
min p-value: 4.93e-08
t-value: 5.922364
ICP_left
alphaFWE: 0.000463
min p-value: 0.025524
t-value: 2.296981
ICP_right
alphaFWE: 0.000392
min p-value: 0.000567
t-value: 3.660025
IFO_left
alphaFWE: 0.000398
min p-value: 0.03138
t-value: 2.183574

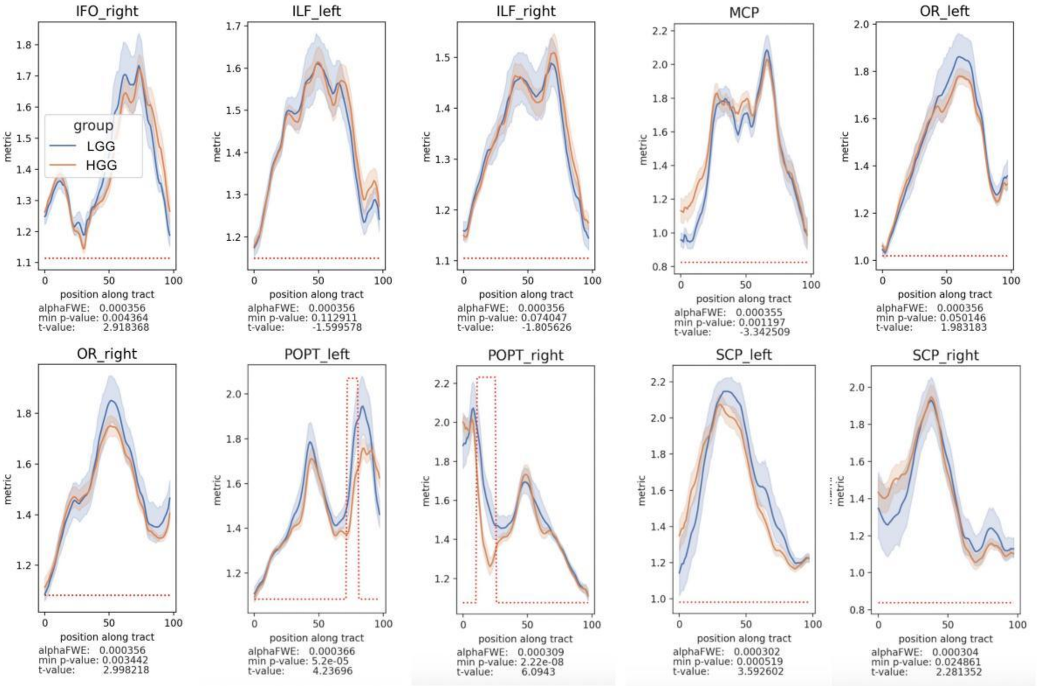

IFO_right
group
LGG
HGG
metric
position along tract
alphaFWE: 0.000356
min p-value: 0.004364
t-value: 2.918368
ILF_left
alphaFWE: 0.000356
min p-value: 0.112911
t-value: -1.599578
ILF_right
alphaFWE: 0.000356
min p-value: 0.074047
t-value: -1.805626
MCP
alphaFWE: 0.000355
min p-value: 0.001197
t-value: -3.342509
OR_left
alphaFWE: 0.000356
min p-value: 0.050146
t-value: 1.983183
OR_right
alphaFWE: 0.000356
min p-value: 0.003442
t-value: 2.998218
POPT_left
alphaFWE: 0.000366
min p-value: 5.2e-05
t-value: 4.23696
POPT_right
alphaFWE: 0.000309
min p-value: 2.22e-08
t-value: 6.0943
SCP_left
alphaFWE: 0.000302
min p-value: 0.000519
t-value: 3.592602
SCP_right
alphaFWE: 0.000304
min p-value: 0.024861
t-value: 2.281352

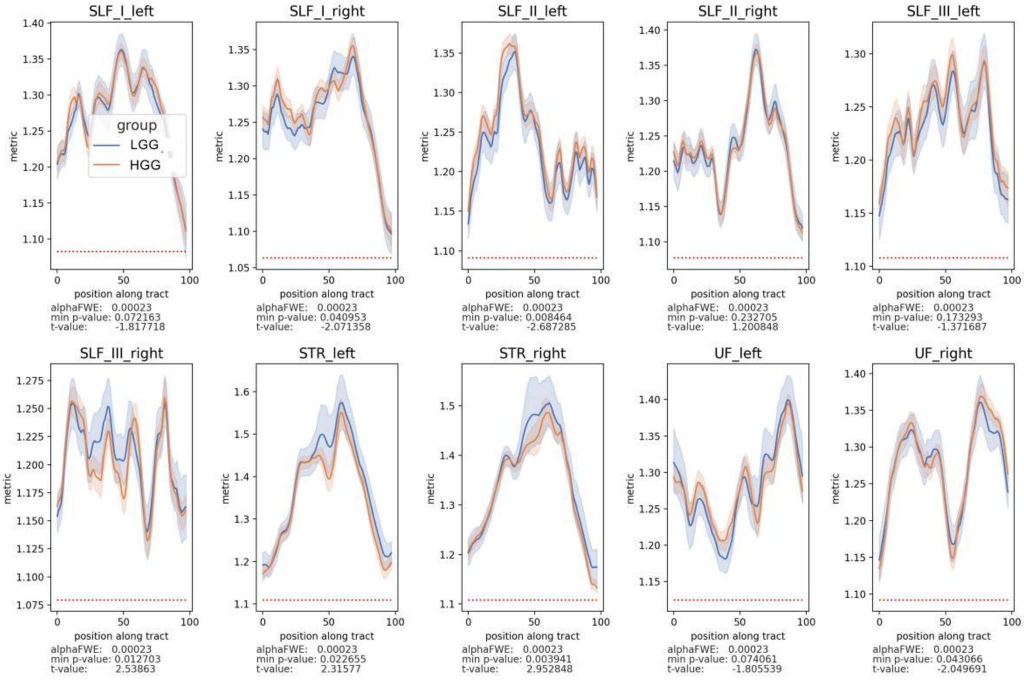

SLF_I_left
group
LGG
HGG
metric
position along tract
alphaFWE: 0.00023
min p-value: 0.072163
t-value: -1.817718
SLF_I_right
alphaFWE: 0.00023
min p-value: 0.040953
t-value: -2.071358
SLF_II_left
alphaFWE: 0.00023
min p-value: 0.008464
t-value: -2.687285
SLF_II_right
alphaFWE: 0.00023
min p-value: 0.232705
t-value: 1.200848
SLF_III_left
alphaFWE: 0.00023
min p-value: 0.173293
t-value: -1.371687
SLF_III_right
alphaFWE: 0.00023
min p-value: 0.012703
t-value: 2.53863
STR_left
alphaFWE: 0.00023
min p-value: 0.022655
t-value: 2.31577
STR_right
alphaFWE: 0.00023
min p-value: 0.003941
t-value: 2.952848
UF_left
alphaFWE: 0.00023
min p-value: 0.074061
t-value: -1.805539
UF_right
alphaFWE: 0.00023
min p-value: 0.043066
t-value: -2.049691

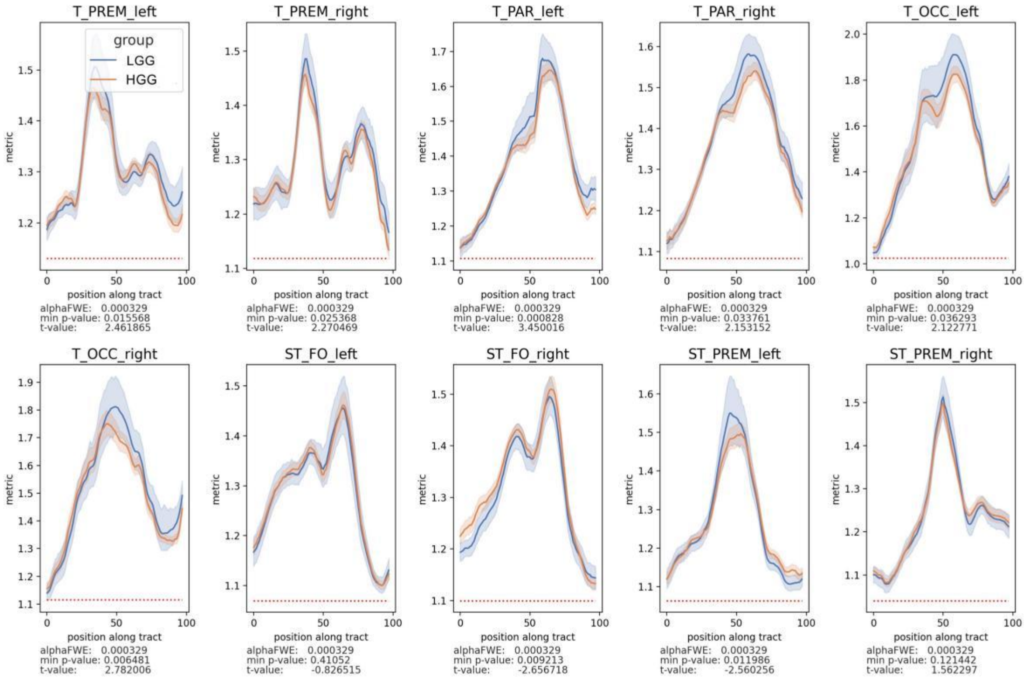

Supplementary Figure 11. Quantitative analysis of AD values of fifty white matter tracts in patients with LGG versus patients with HGG.

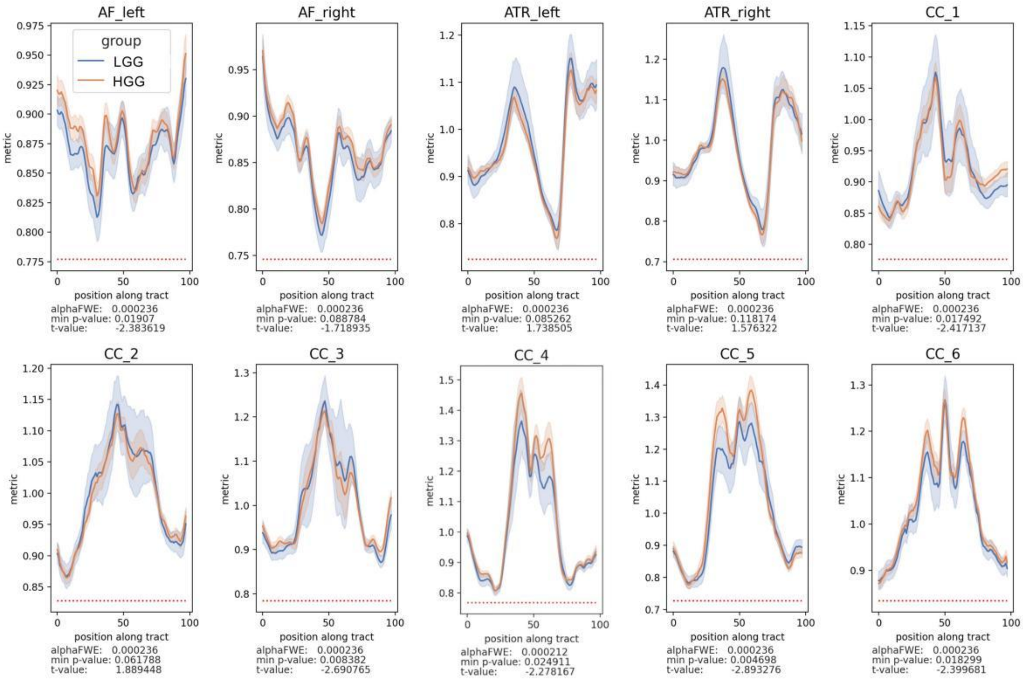
AF_left
group
LGG
HGG
alphaFWE: 0.000236
min p-value: 0.01907
t-value: -2.383619
AF_right
alphaFWE: 0.000236
min p-value: 0.088784
t-value: -1.718935
ATR_left
alphaFWE: 0.000236
min p-value: 0.085262
t-value: 1.738505
ATR_right
alphaFWE: 0.000236
min p-value: 0.118174
t-value: 1.576322
CC_1
alphaFWE: 0.000236
min p-value: 0.017492
t-value: -2.417137
CC_2
alphaFWE: 0.000236
min p-value: 0.061788
t-value: 1.889448
CC_3
alphaFWE: 0.000236
min p-value: 0.008382
t-value: -2.690765
CC_4
alphaFWE: 0.000212
min p-value: 0.024911
t-value: -2.278167
CC_5
alphaFWE: 0.000236
min p-value: 0.004698
t-value: -2.893276
CC_6
alphaFWE: 0.000236
min p-value: 0.018299
t-value: -2.399681
metric
position along tract

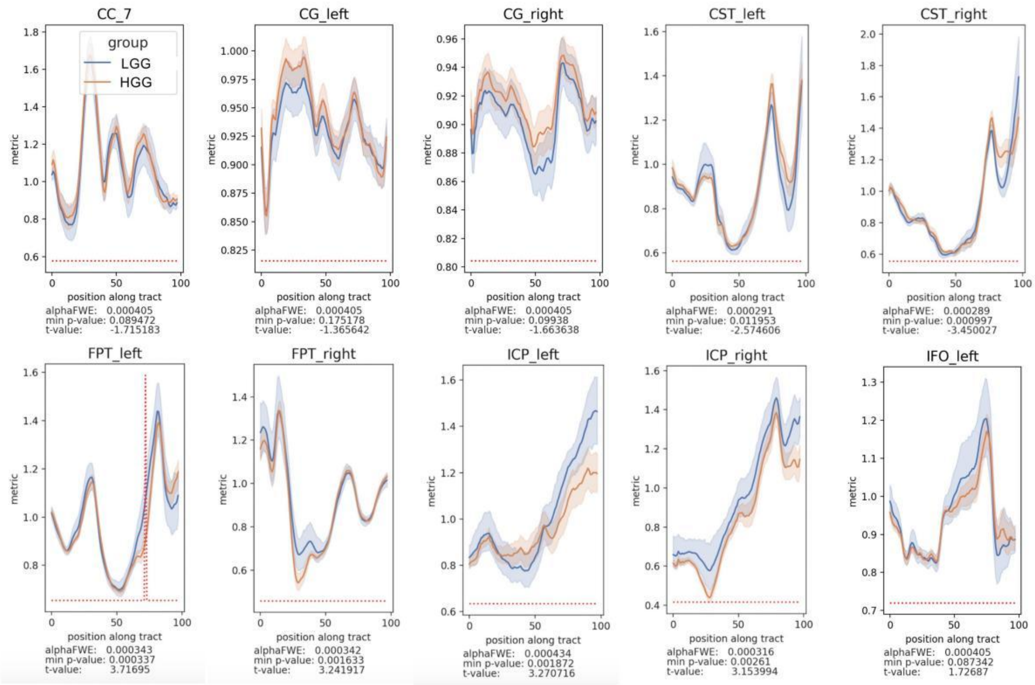
CC_7
group
LGG
HGG
alphaFWE: 0.000405
min p-value: 0.089472
t-value: -1.715183
CG_left
alphaFWE: 0.000405
min p-value: 0.175178
t-value: -1.365642
CG_right
alphaFWE: 0.000405
min p-value: 0.09938
t-value: -1.663638
CST_left
alphaFWE: 0.000291
min p-value: 0.011953
t-value: -2.574606
CST_right
alphaFWE: 0.000289
min p-value: 0.000997
t-value: -3.450027
FPT_left
alphaFWE: 0.000343
min p-value: 0.000337
t-value: 3.71695
FPT_right
alphaFWE: 0.000342
min p-value: 0.001633
t-value: 3.241917
ICP_left
alphaFWE: 0.000434
min p-value: 0.001872
t-value: 3.270716
ICP_right
alphaFWE: 0.000316
min p-value: 0.00261
t-value: 3.153994
IFO_left
alphaFWE: 0.000405
min p-value: 0.087342
t-value: 1.72687
metric
position along tract

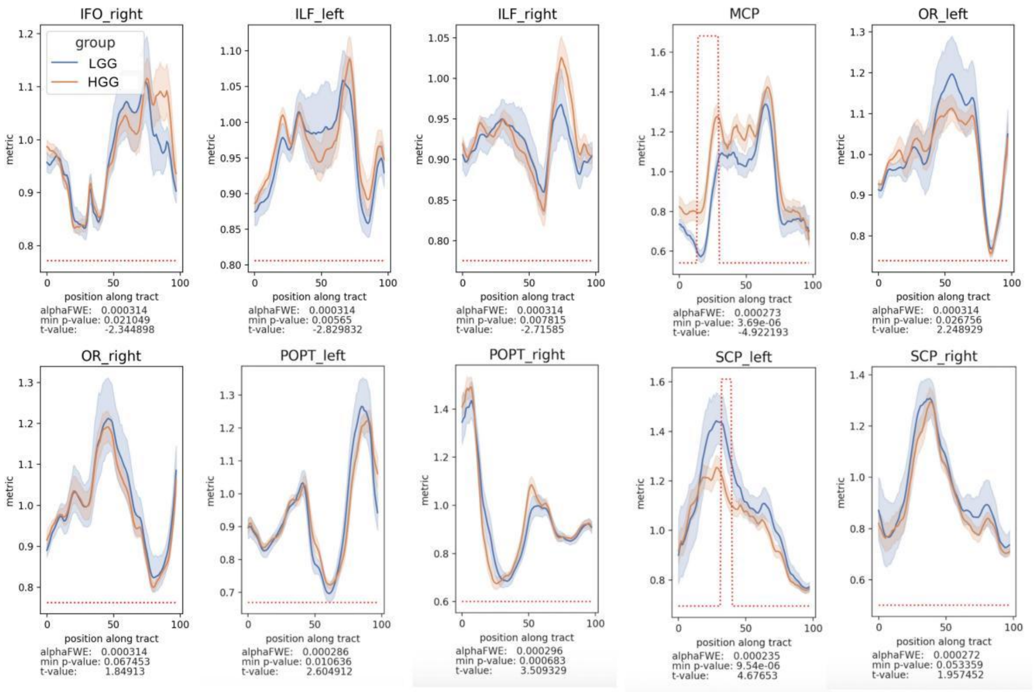

IFO_right
group
LGG
HGG
metric
position along tract
alphaFWE: 0.000314
min p-value: 0.021049
t-value: -2.344898
ILF_left
alphaFWE: 0.000314
min p-value: 0.00565
t-value: -2.829832
ILF_right
alphaFWE: 0.000314
min p-value: 0.007815
t-value: -2.71585
MCP
alphaFWE: 0.000273
min p-value: 3.69e-06
t-value: -4.922193
OR_left
alphaFWE: 0.000314
min p-value: 0.026756
t-value: 2.248929
OR_right
alphaFWE: 0.000314
min p-value: 0.067453
t-value: 1.84913
POPT_left
alphaFWE: 0.000286
min p-value: 0.010636
t-value: 2.604912
POPT_right
alphaFWE: 0.000296
min p-value: 0.000683
t-value: 3.509329
SCP_left
alphaFWE: 0.000235
min p-value: 9.54e-06
t-value: 4.67653
SCP_right
alphaFWE: 0.000272
min p-value: 0.053359
t-value: 1.957452

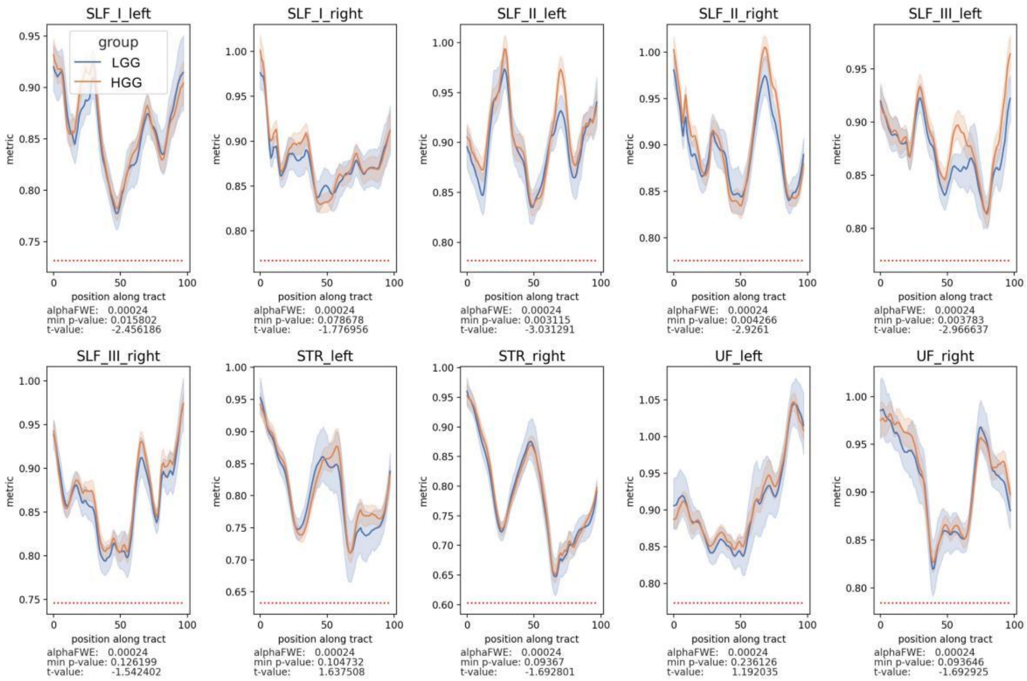

SLF_I_left
group
LGG
HGG
metric
position along tract
alphaFWE: 0.00024
min p-value: 0.015802
t-value: -2.456186
SLF_I_right
alphaFWE: 0.00024
min p-value: 0.078678
t-value: -1.776956
SLF_II_left
alphaFWE: 0.00024
min p-value: 0.003115
t-value: -3.031291
SLF_II_right
alphaFWE: 0.00024
min p-value: 0.004266
t-value: -2.9261
SLF_III_left
alphaFWE: 0.00024
min p-value: 0.003783
t-value: -2.966637
SLF_III_right
alphaFWE: 0.00024
min p-value: 0.126199
t-value: -1.542402
STR_left
alphaFWE: 0.00024
min p-value: 0.104732
t-value: 1.637508
STR_right
alphaFWE: 0.00024
min p-value: 0.09367
t-value: -1.692801
UF_left
alphaFWE: 0.00024
min p-value: 0.236126
t-value: 1.192035
UF_right
alphaFWE: 0.00024
min p-value: 0.093646
t-value: -1.692925

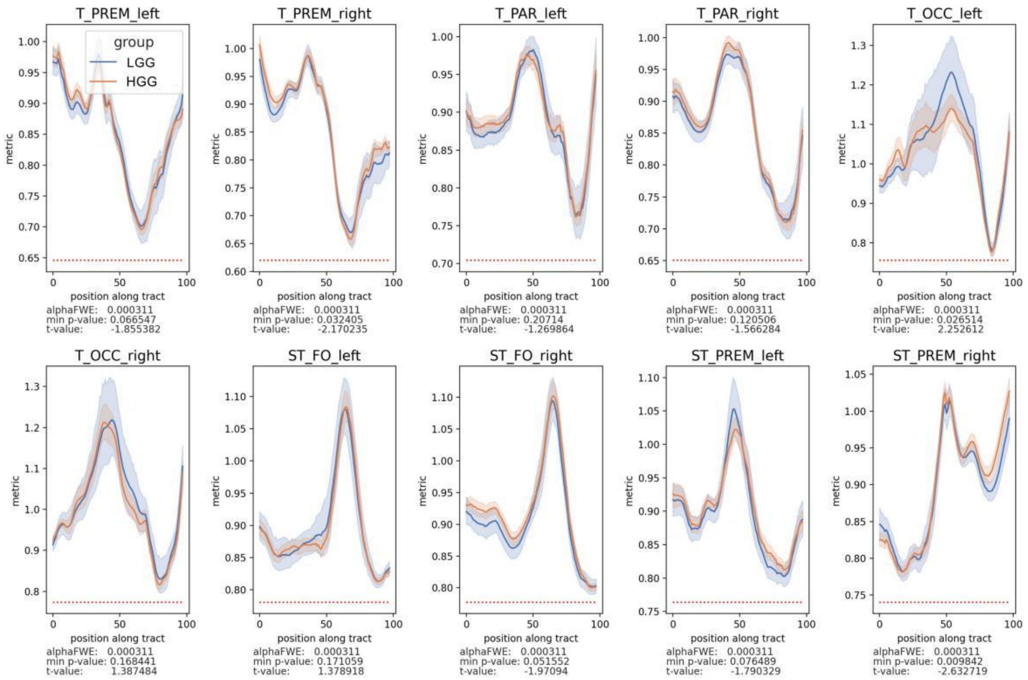

Supplementary Figure 12. Quantitative analysis of RD values of fifty white matter tracts in patients with LGG versus patients with HGG.

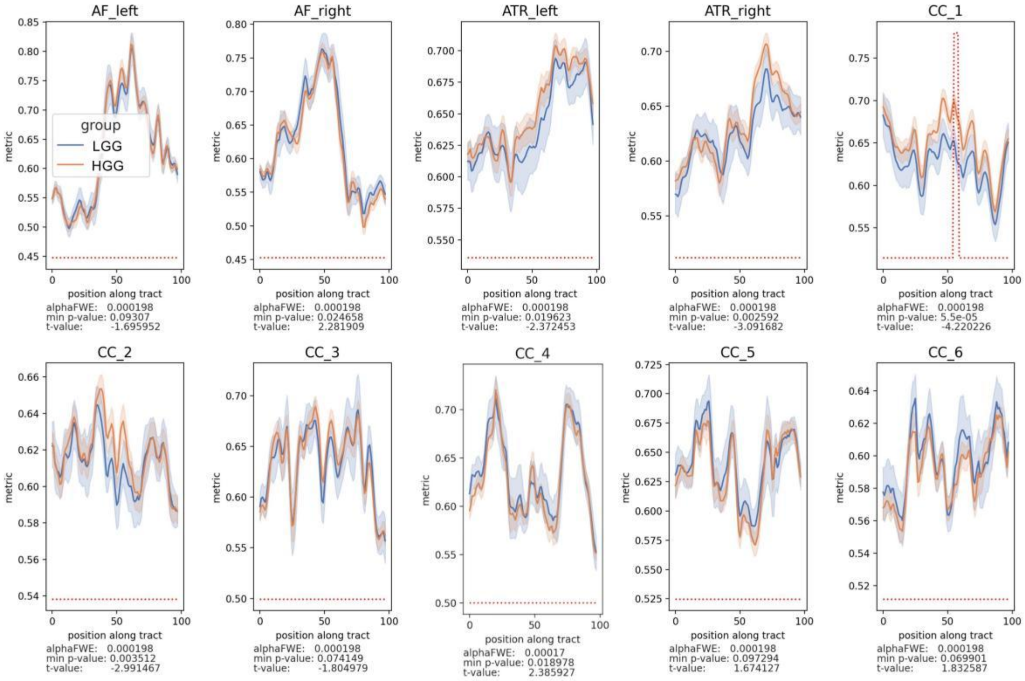

AF_left
group
LGG
HGG
metric
position along tract
alphaFWE: 0.000198
min p-value: 0.09307
t-value: -1.695952
AF_right
alphaFWE: 0.000198
min p-value: 0.024658
t-value: 2.281909
ATR_left
alphaFWE: 0.000198
min p-value: 0.019623
t-value: -2.372453
ATR_right
alphaFWE: 0.000198
min p-value: 0.002592
t-value: -3.091682
CC_1
alphaFWE: 0.000198
min p-value: 5.5e-05
t-value: -4.220226
CC_2
alphaFWE: 0.000198
min p-value: 0.003512
t-value: -2.991467
CC_3
alphaFWE: 0.000198
min p-value: 0.074149
t-value: -1.804979
CC_4
alphaFWE: 0.00017
min p-value: 0.018978
t-value: 2.385927
CC_5
alphaFWE: 0.000198
min p-value: 0.097294
t-value: 1.674127
CC_6
alphaFWE: 0.000198
min p-value: 0.069901
t-value: 1.832587

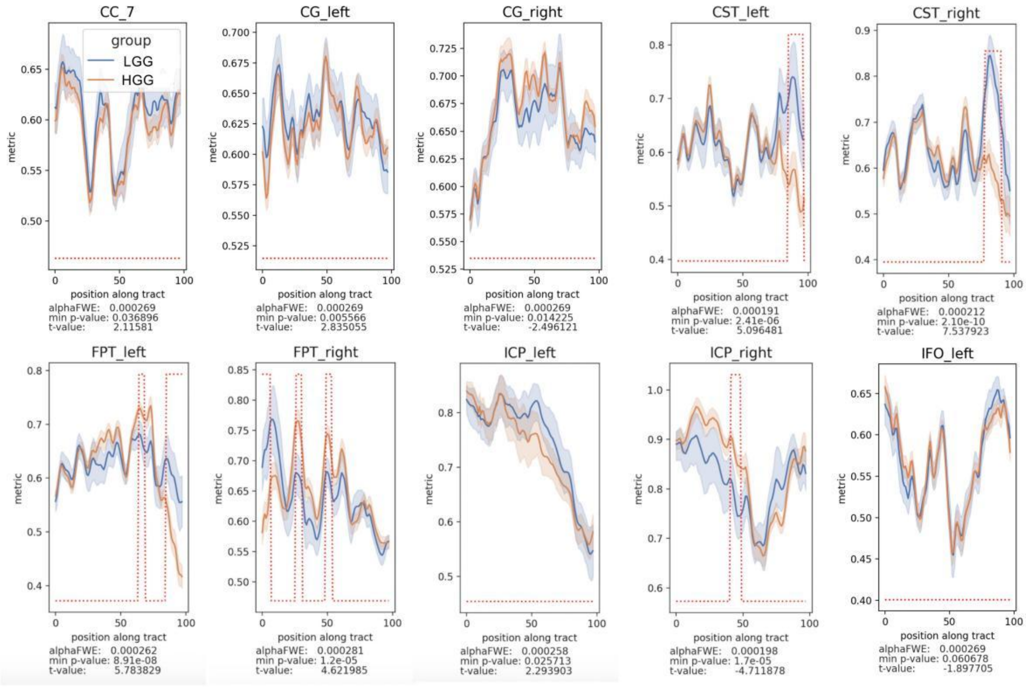

CC_7
group
LGG
HGG
metric
position along tract
alphaFWE: 0.000269
min p-value: 0.036896
t-value: 2.11581
CG_left
alphaFWE: 0.000269
min p-value: 0.005566
t-value: 2.835055
CG_right
alphaFWE: 0.000269
min p-value: 0.014225
t-value: -2.496121
CST_left
alphaFWE: 0.000191
min p-value: 2.41e-06
t-value: 5.096481
CST_right
alphaFWE: 0.000212
min p-value: 2.10e-10
t-value: 7.537923
FPT_left
alphaFWE: 0.000262
min p-value: 8.91e-08
t-value: 5.783829
FPT_right
alphaFWE: 0.000281
min p-value: 1.2e-05
t-value: 4.621985
ICP_left
alphaFWE: 0.000258
min p-value: 0.025713
t-value: 2.293903
ICP_right
alphaFWE: 0.000198
min p-value: 1.7e-05
t-value: -4.711878
IFO_left
alphaFWE: 0.000269
min p-value: 0.060678
t-value: -1.897705

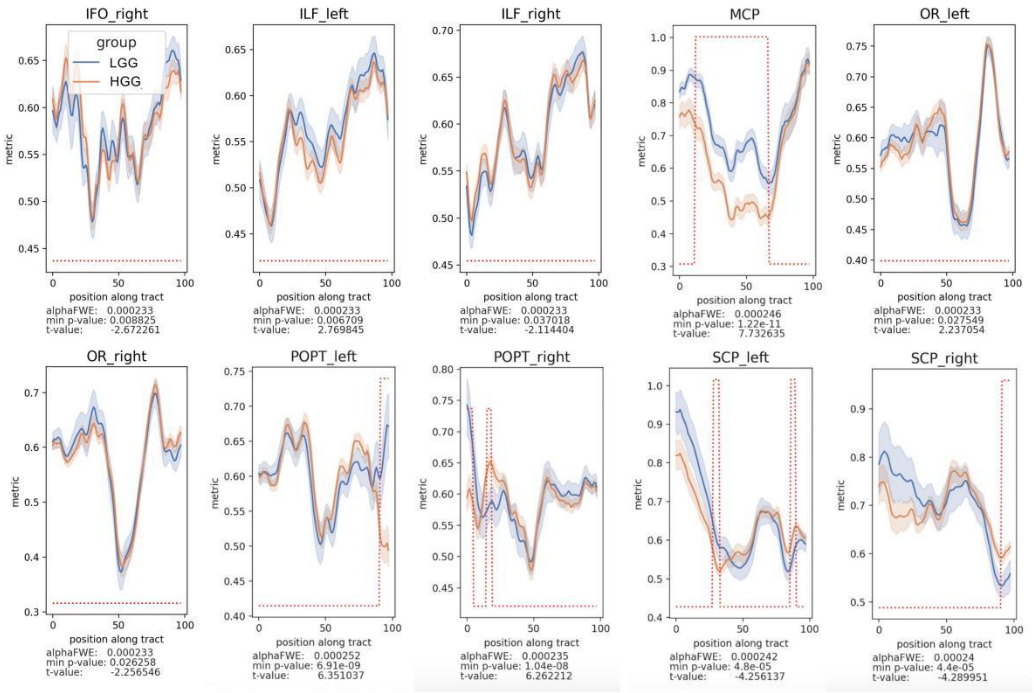
IFO_right
group
LGG
HGG
alphaFWE: 0.000233
min p-value: 0.008825
t-value: -2.672261
ILF_left
alphaFWE: 0.000233
min p-value: 0.006709
t-value: 2.769845
ILF_right
alphaFWE: 0.000233
min p-value: 0.037018
t-value: -2.114404
MCP
alphaFWE: 0.000246
min p-value: 1.22e-11
t-value: 7.732635
OR_left
alphaFWE: 0.000233
min p-value: 0.027549
t-value: 2.237054
OR_right
alphaFWE: 0.000233
min p-value: 0.026258
t-value: -2.256546
POPT_left
alphaFWE: 0.000252
min p-value: 6.91e-09
t-value: 6.351037
POPT_right
alphaFWE: 0.000235
min p-value: 1.04e-08
t-value: 6.262212
SCP_left
alphaFWE: 0.000242
min p-value: 4.8e-05
t-value: -4.256137
SCP_right
alphaFWE: 0.00024
min p-value: 4.4e-05
t-value: -4.289951
metric
position along tract

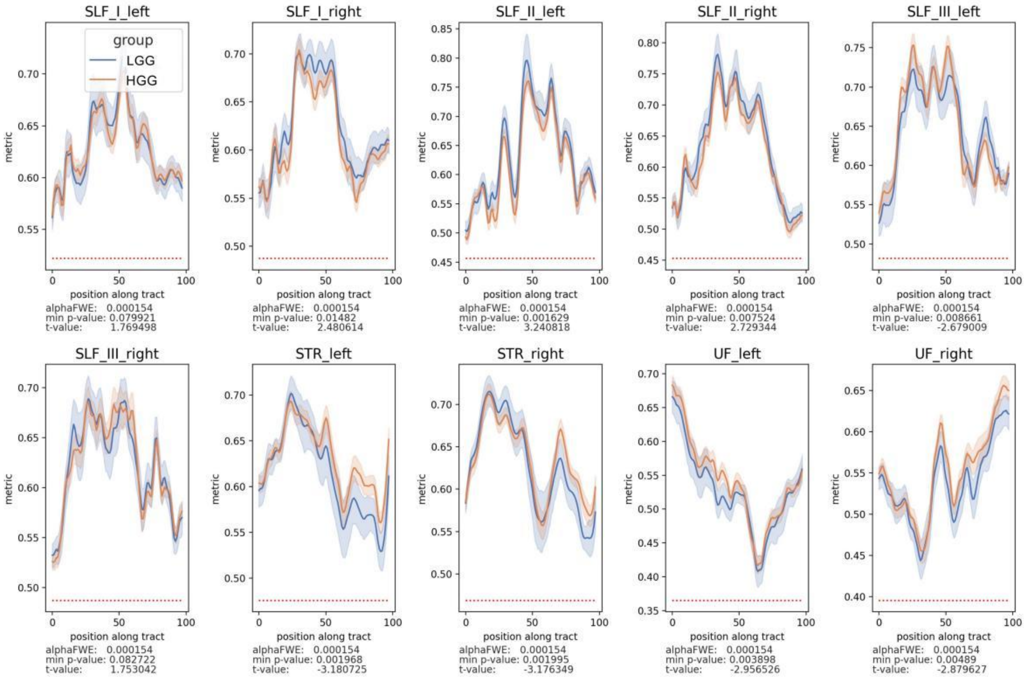
SLF_I_left
group
LGG
HGG
alphaFWE: 0.000154
min p-value: 0.079921
t-value: 1.769498
SLF_I_right
alphaFWE: 0.000154
min p-value: 0.01482
t-value: 2.480614
SLF_II_left
alphaFWE: 0.000154
min p-value: 0.001629
t-value: 3.240818
SLF_II_right
alphaFWE: 0.000154
min p-value: 0.007524
t-value: 2.729344
SLF_III_left
alphaFWE: 0.000154
min p-value: 0.008661
t-value: -2.679009
SLF_III_right
alphaFWE: 0.000154
min p-value: 0.082722
t-value: 1.753042
STR_left
alphaFWE: 0.000154
min p-value: 0.001968
t-value: -3.180725
STR_right
alphaFWE: 0.000154
min p-value: 0.001995
t-value: -3.176349
UF_left
alphaFWE: 0.000154
min p-value: 0.003898
t-value: -2.956526
UF_right
alphaFWE: 0.000154
min p-value: 0.00489
t-value: -2.879627
metric
position along tract

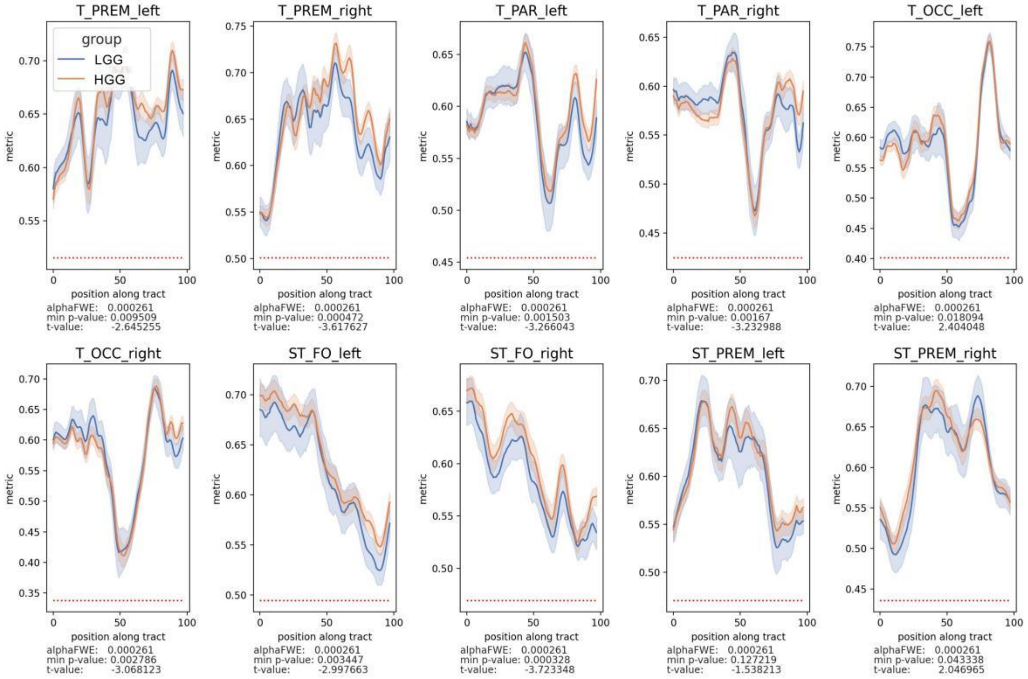

Supplementary Figure 13. Quantitative analysis of AK values of fifty white matter tracts in patients with LGG versus patients with HGG.

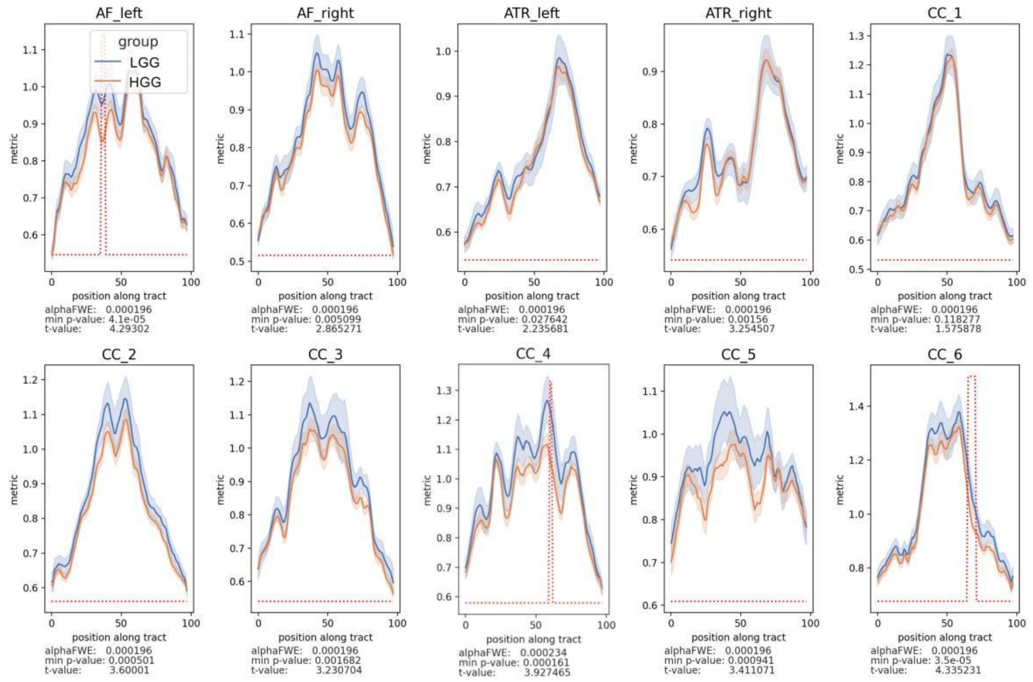

AF_left
group
LGG
HGG
metric
position along tract
alphaFWE: 0.000196
min p-value: 4.1e-05
t-value: 4.29302
AF_right
alphaFWE: 0.000196
min p-value: 0.005099
t-value: 2.865271
ATR_left
alphaFWE: 0.000196
min p-value: 0.027642
t-value: 2.235681
ATR_right
alphaFWE: 0.000196
min p-value: 0.00156
t-value: 3.254507
CC_1
alphaFWE: 0.000196
min p-value: 0.118277
t-value: 1.575878
CC_2
alphaFWE: 0.000196
min p-value: 0.000501
t-value: 3.60001
CC_3
alphaFWE: 0.000196
min p-value: 0.001682
t-value: 3.230704
CC_4
alphaFWE: 0.000234
min p-value: 0.000161
t-value: 3.927465
CC_5
alphaFWE: 0.000196
min p-value: 0.000941
t-value: 3.411071
CC_6
alphaFWE: 0.000196
min p-value: 3.5e-05
t-value: 4.335231

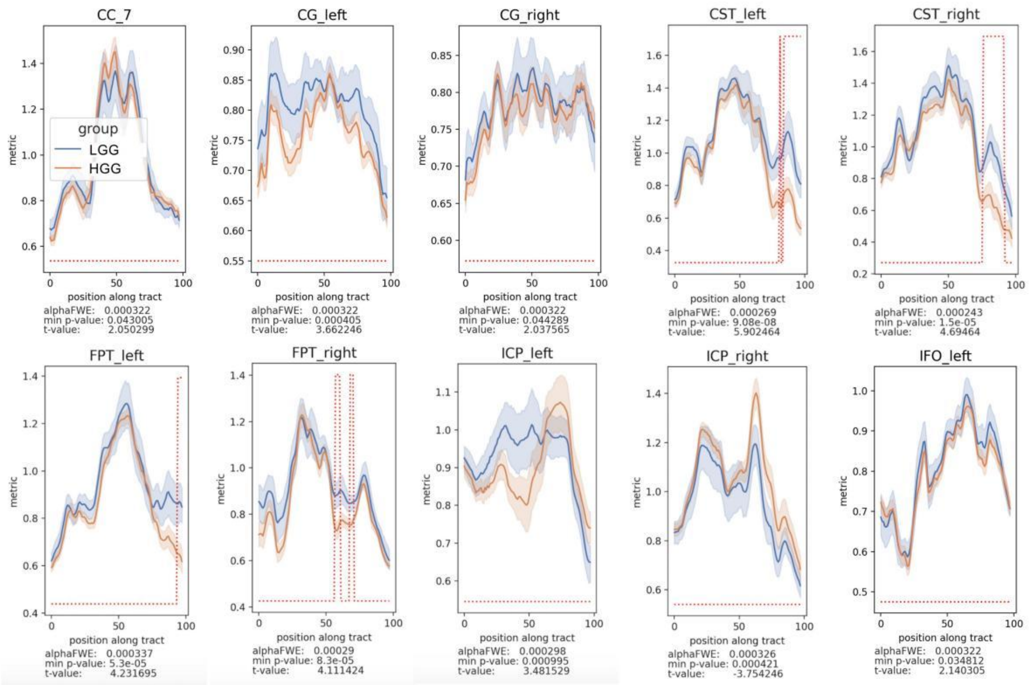

CC_7
group
LGG
HGG
metric
position along tract
alphaFWE: 0.000322
min p-value: 0.043005
t-value: 2.050299
CG_left
alphaFWE: 0.000322
min p-value: 0.000405
t-value: 3.662246
CG_right
alphaFWE: 0.000322
min p-value: 0.044289
t-value: 2.037565
CST_left
alphaFWE: 0.000269
min p-value: 9.08e-08
t-value: 5.902464
CST_right
alphaFWE: 0.000243
min p-value: 1.5e-05
t-value: 4.69464
FPT_left
alphaFWE: 0.000337
min p-value: 5.3e-05
t-value: 4.231695
FPT_right
alphaFWE: 0.00029
min p-value: 8.3e-05
t-value: 4.111424
ICP_left
alphaFWE: 0.000298
min p-value: 0.000995
t-value: 3.481529
ICP_right
alphaFWE: 0.000326
min p-value: 0.000421
t-value: -3.754246
IFO_left
alphaFWE: 0.000322
min p-value: 0.034812
t-value: 2.140305

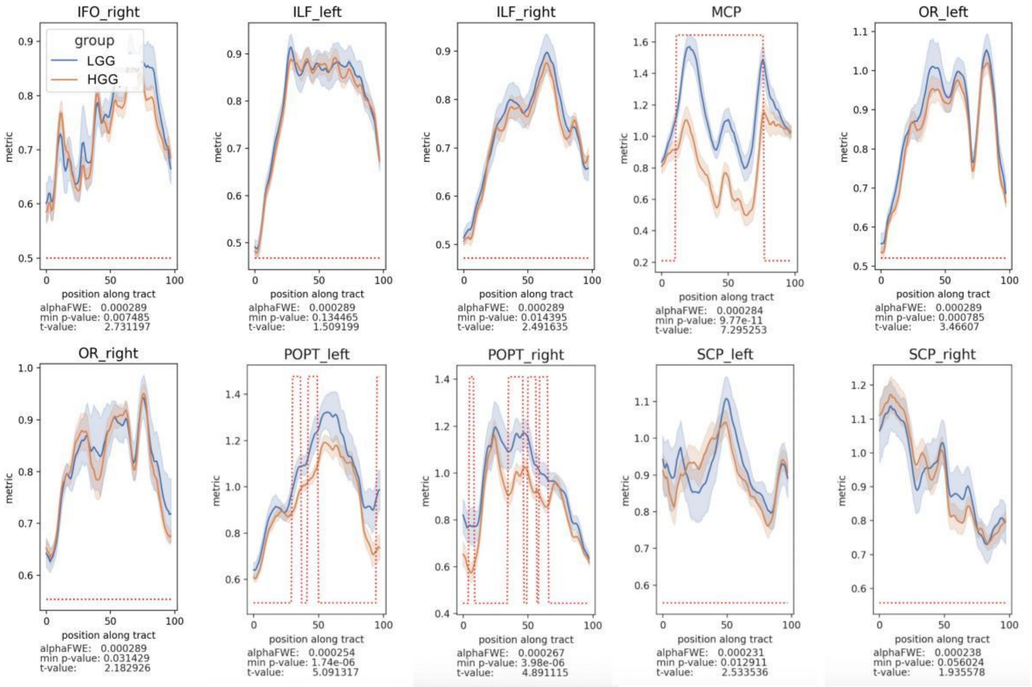
IFO_right
group
LGG
HGG
metric
position along tract
alphaFWE: 0.000289
min p-value: 0.007485
t-value: 2.731197
ILF_left
alphaFWE: 0.000289
min p-value: 0.134465
t-value: 1.509199
ILF_right
alphaFWE: 0.000289
min p-value: 0.014395
t-value: 2.491635
MCP
alphaFWE: 0.000284
min p-value: 9.77e-11
t-value: 7.295253
OR_left
alphaFWE: 0.000289
min p-value: 0.000785
t-value: 3.46607
OR_right
alphaFWE: 0.000289
min p-value: 0.031429
t-value: 2.182926
POPT_left
alphaFWE: 0.000254
min p-value: 1.74e-06
t-value: 5.091317
POPT_right
alphaFWE: 0.000267
min p-value: 3.98e-06
t-value: 4.891115
SCP_left
alphaFWE: 0.000231
min p-value: 0.012911
t-value: 2.533536
SCP_right
alphaFWE: 0.000238
min p-value: 0.056024
t-value: 1.935578

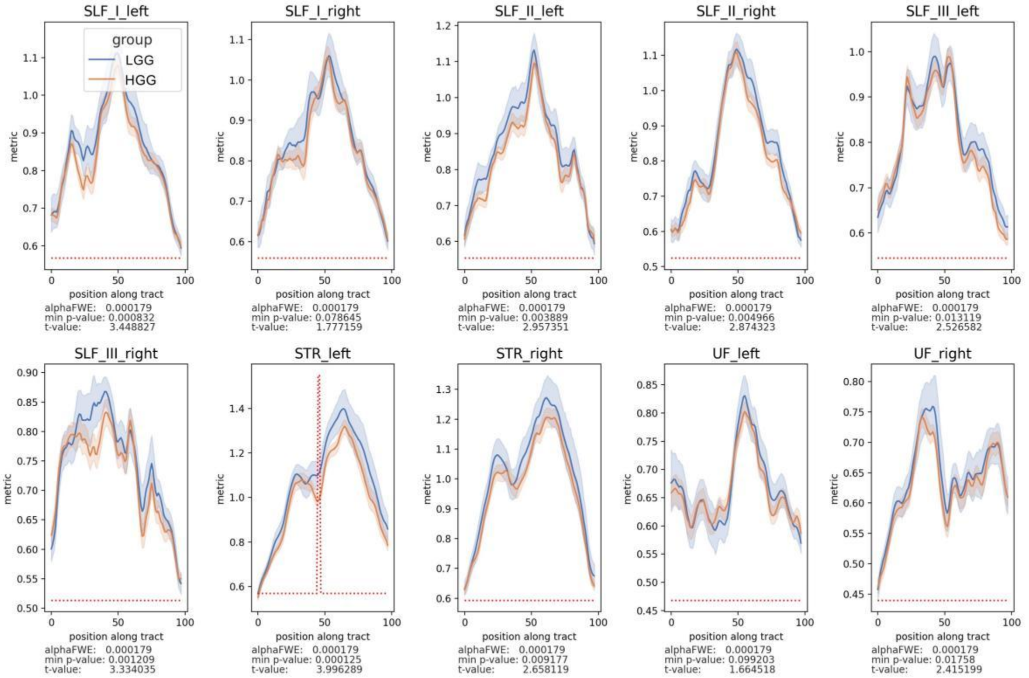
SLF_I_left
group
LGG
HGG
metric
position along tract
alphaFWE: 0.000179
min p-value: 0.000832
t-value: 3.448827
SLF_I_right
alphaFWE: 0.000179
min p-value: 0.078645
t-value: 1.777159
SLF_II_left
alphaFWE: 0.000179
min p-value: 0.003889
t-value: 2.957351
SLF_II_right
alphaFWE: 0.000179
min p-value: 0.004966
t-value: 2.874323
SLF_III_left
alphaFWE: 0.000179
min p-value: 0.013119
t-value: 2.526582
SLF_III_right
alphaFWE: 0.000179
min p-value: 0.001209
t-value: 3.334035
STR_left
alphaFWE: 0.000179
min p-value: 0.000125
t-value: 3.996289
STR_right
alphaFWE: 0.000179
min p-value: 0.009177
t-value: 2.658119
UF_left
alphaFWE: 0.000179
min p-value: 0.099203
t-value: 1.664518
UF_right
alphaFWE: 0.000179
min p-value: 0.01758
t-value: 2.415199

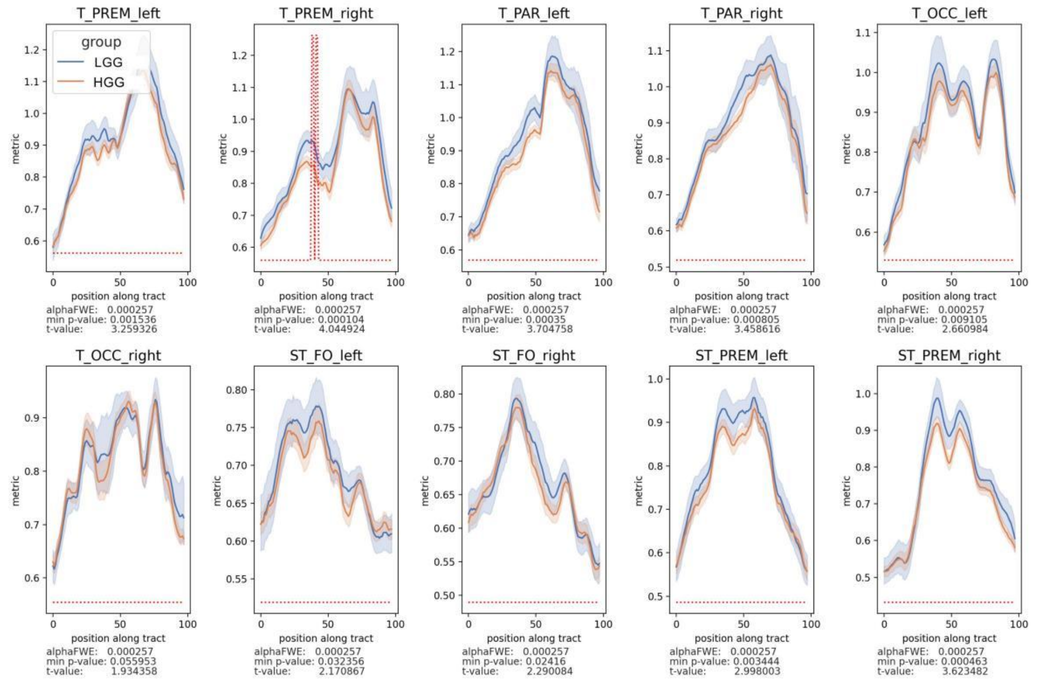

Supplementary Figure 14. Quantitative analysis of RK values of fifty white matter tracts in patients with LGG versus patients with HGG.

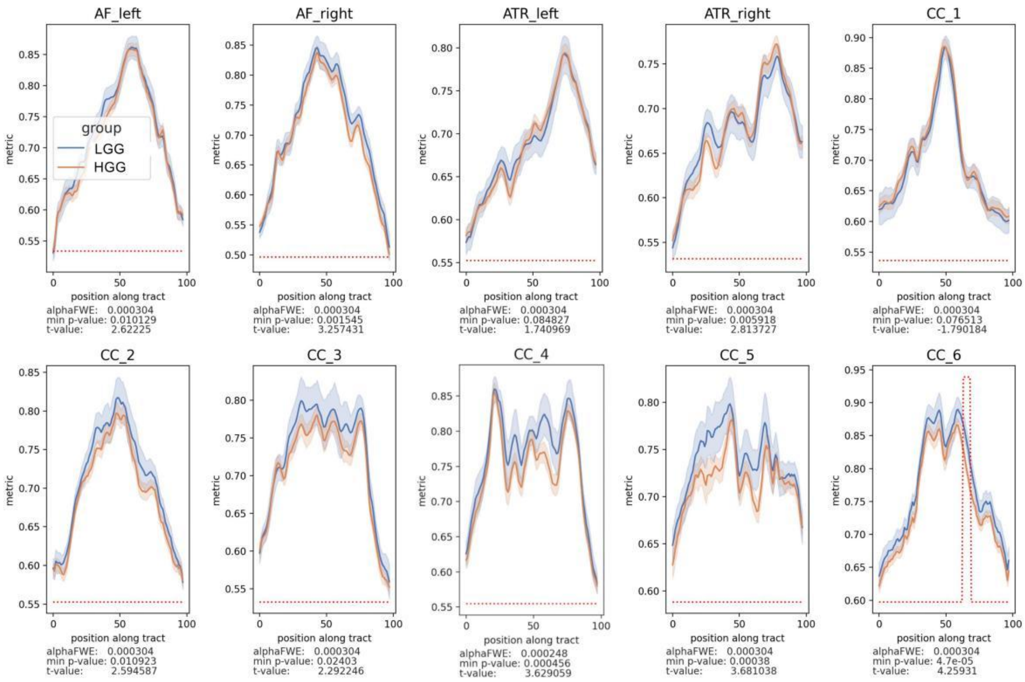
AF_left
group
LGG
HGG
metric
position along tract
alphaFWE: 0.000304
min p-value: 0.010129
t-value: 2.62225
AF_right
alphaFWE: 0.000304
min p-value: 0.001545
t-value: 3.257431
ATR_left
alphaFWE: 0.000304
min p-value: 0.084827
t-value: 1.740969
ATR_right
alphaFWE: 0.000304
min p-value: 0.005918
t-value: 2.813727
CC_1
alphaFWE: 0.000304
min p-value: 0.076513
t-value: -1.790184
CC_2
alphaFWE: 0.000304
min p-value: 0.010923
t-value: 2.594587
CC_3
alphaFWE: 0.000304
min p-value: 0.02403
t-value: 2.292246
CC_4
alphaFWE: 0.000248
min p-value: 0.000456
t-value: 3.629059
CC_5
alphaFWE: 0.000304
min p-value: 0.00038
t-value: 3.681038
CC_6
alphaFWE: 0.000304
min p-value: 4.7e-05
t-value: 4.25931

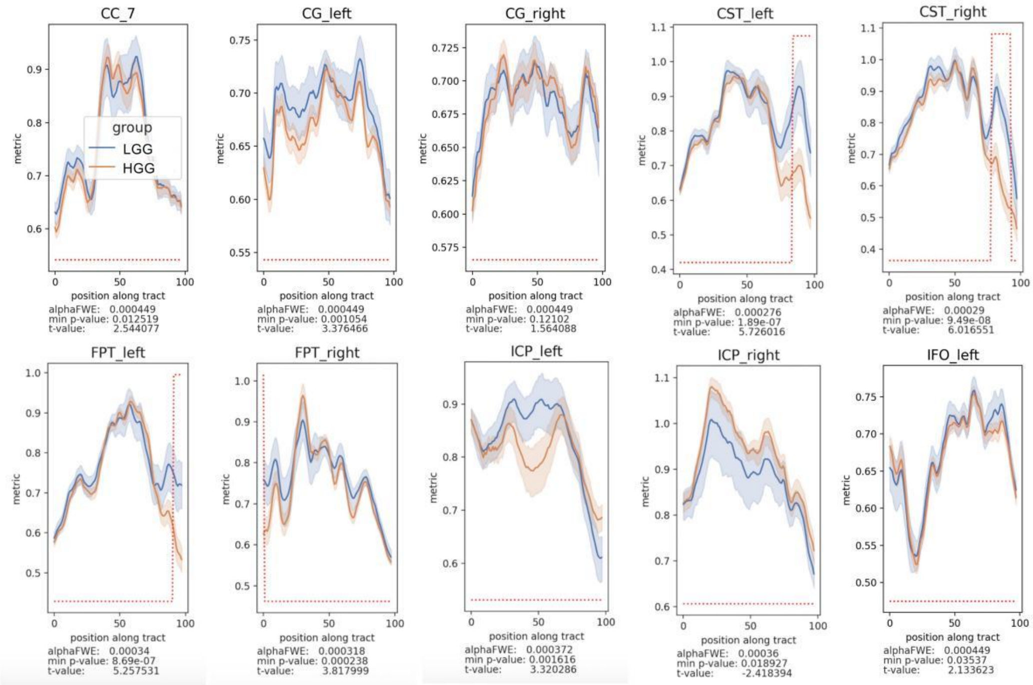
CC_7
group
LGG
HGG
metric
position along tract
alphaFWE: 0.000449
min p-value: 0.012519
t-value: 2.544077
CG_left
alphaFWE: 0.000449
min p-value: 0.001054
t-value: 3.376466
CG_right
alphaFWE: 0.000449
min p-value: 0.12102
t-value: 1.564088
CST_left
alphaFWE: 0.000276
min p-value: 1.89e-07
t-value: 5.726016
CST_right
alphaFWE: 0.00029
min p-value: 9.49e-08
t-value: 6.016551
FPT_left
alphaFWE: 0.00034
min p-value: 8.69e-07
t-value: 5.257531
FPT_right
alphaFWE: 0.000318
min p-value: 0.000238
t-value: 3.817999
ICP_left
alphaFWE: 0.000372
min p-value: 0.001616
t-value: 3.320286
ICP_right
alphaFWE: 0.00036
min p-value: 0.018927
t-value: -2.418394
IFO_left
alphaFWE: 0.000449
min p-value: 0.03537
t-value: 2.133623

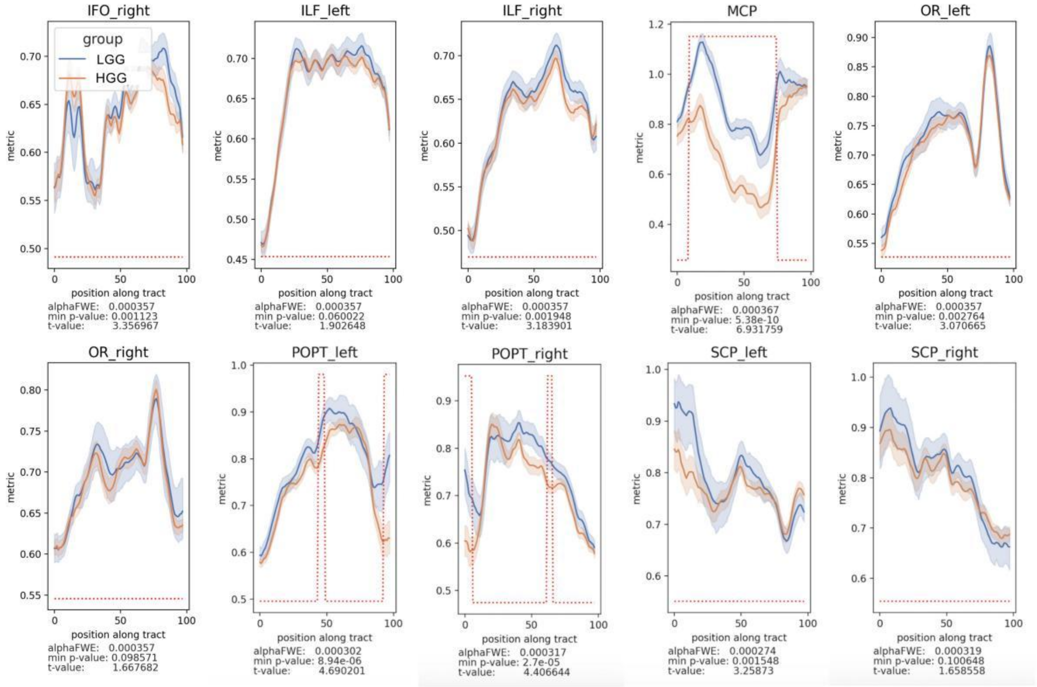
IFO_right
group
LGG
HGG
metric
position along tract
alphaFWE: 0.000357
min p-value: 0.001123
t-value: 3.356967
ILF_left
alphaFWE: 0.000357
min p-value: 0.060022
t-value: 1.902648
ILF_right
alphaFWE: 0.000357
min p-value: 0.001948
t-value: 3.183901
MCP
alphaFWE: 0.000367
min p-value: 5.38e-10
t-value: 6.931759
OR_left
alphaFWE: 0.000357
min p-value: 0.002764
t-value: 3.070665
OR_right
alphaFWE: 0.000357
min p-value: 0.098571
t-value: 1.667682
POPT_left
alphaFWE: 0.000302
min p-value: 8.94e-06
t-value: 4.690201
POPT_right
alphaFWE: 0.000317
min p-value: 2.7e-05
t-value: 4.406644
SCP_left
alphaFWE: 0.000274
min p-value: 0.001548
t-value: 3.25873
SCP_right
alphaFWE: 0.000319
min p-value: 0.100648
t-value: 1.658558

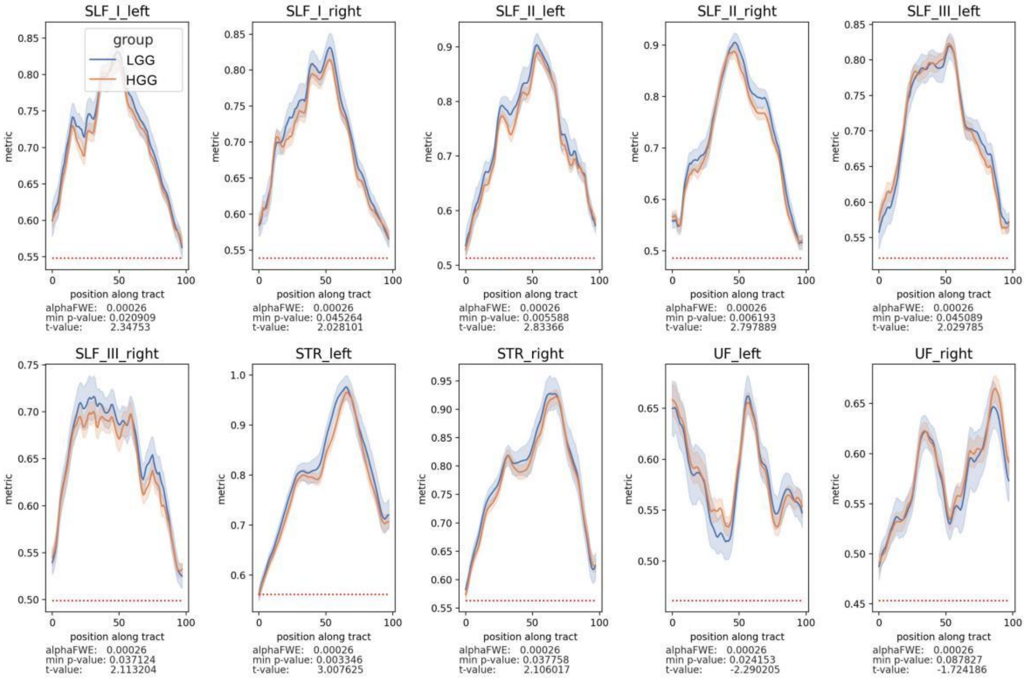
SLF_I_left
group
LGG
HGG
metric
position along tract
alphaFWE: 0.00026
min p-value: 0.020909
t-value: 2.34753
SLF_I_right
alphaFWE: 0.00026
min p-value: 0.045264
t-value: 2.028101
SLF_II_left
alphaFWE: 0.00026
min p-value: 0.005588
t-value: 2.83366
SLF_II_right
alphaFWE: 0.00026
min p-value: 0.006193
t-value: 2.797889
SLF_III_left
alphaFWE: 0.00026
min p-value: 0.045089
t-value: 2.029785
SLF_III_right
alphaFWE: 0.00026
min p-value: 0.037124
t-value: 2.113204
STR_left
alphaFWE: 0.00026
min p-value: 0.003346
t-value: 3.007625
STR_right
alphaFWE: 0.00026
min p-value: 0.037758
t-value: 2.106017
UF_left
alphaFWE: 0.00026
min p-value: 0.024153
t-value: -2.290205
UF_right
alphaFWE: 0.00026
min p-value: 0.087827
t-value: -1.724186

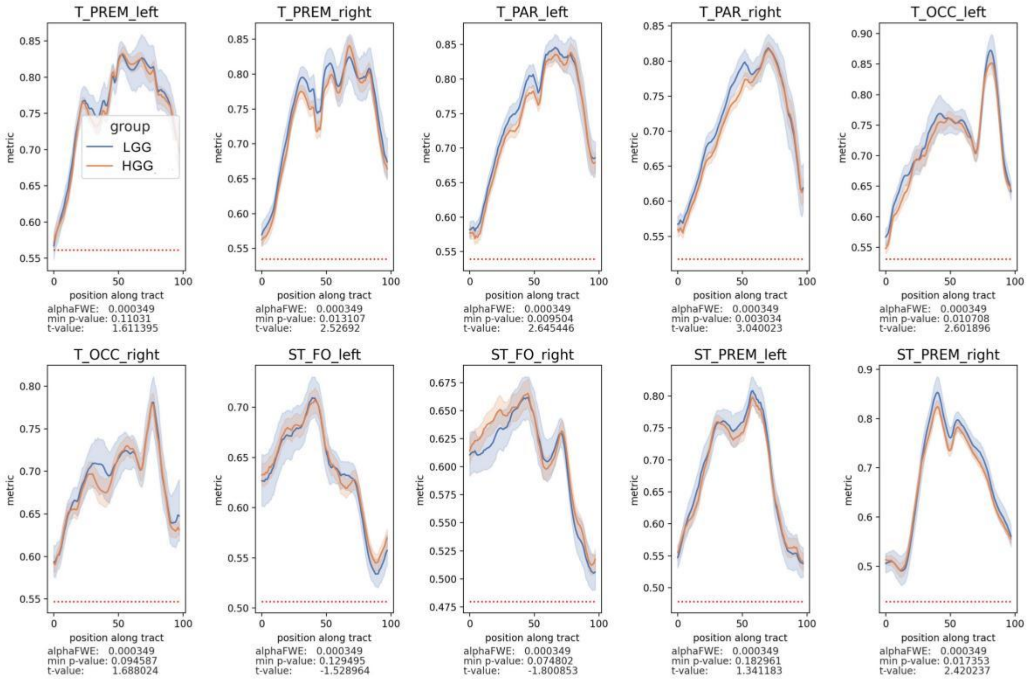

Supplementary Figure 15. Quantitative analysis of MK values of fifty white matter tracts in patients with LGG versus patients with HGG.

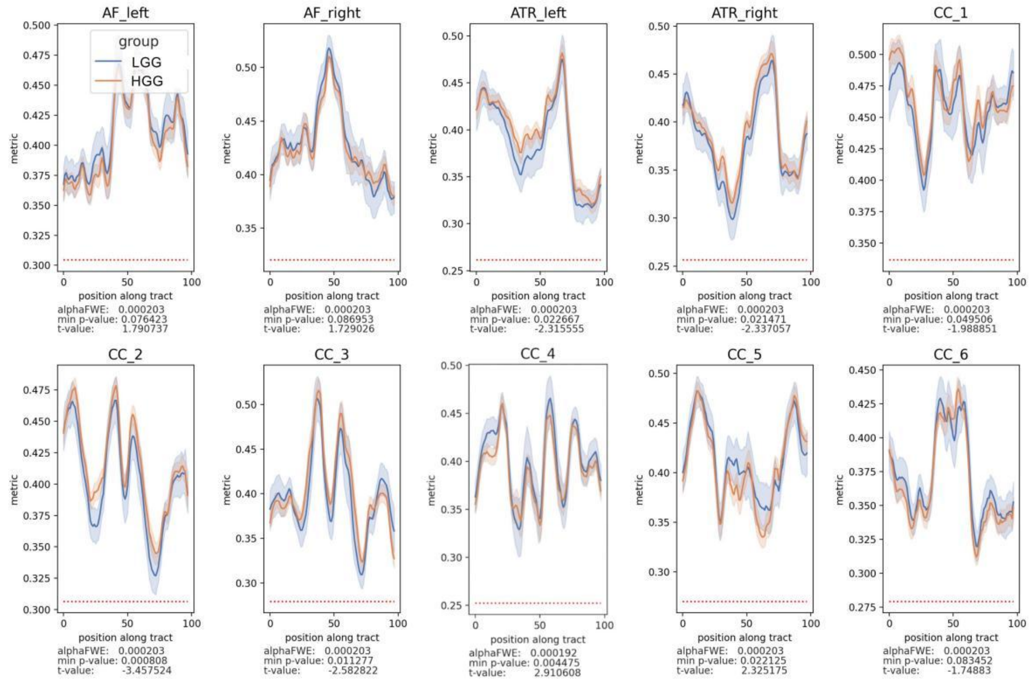

AF_left
group
LGG
HGG
metric
position along tract
alphaFWE: 0.000203
min p-value: 0.076423
t-value: 1.790737
AF_right
alphaFWE: 0.000203
min p-value: 0.086953
t-value: 1.729026
ATR_left
alphaFWE: 0.000203
min p-value: 0.022667
t-value: -2.315555
ATR_right
alphaFWE: 0.000203
min p-value: 0.021471
t-value: -2.337057
CC_1
alphaFWE: 0.000203
min p-value: 0.049506
t-value: -1.988851
CC_2
alphaFWE: 0.000203
min p-value: 0.000808
t-value: -3.457524
CC_3
alphaFWE: 0.000203
min p-value: 0.011277
t-value: -2.582822
CC_4
alphaFWE: 0.000192
min p-value: 0.004475
t-value: 2.910608
CC_5
alphaFWE: 0.000203
min p-value: 0.022125
t-value: 2.325175
CC_6
alphaFWE: 0.000203
min p-value: 0.083452
t-value: -1.74883

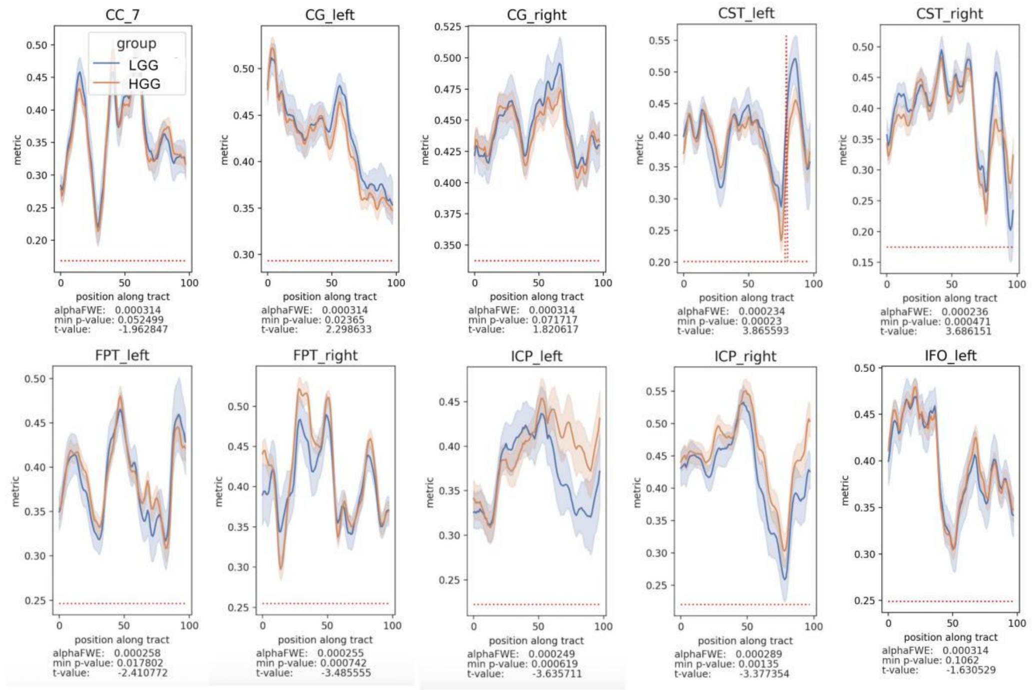

CC_7
group
LGG
HGG
metric
position along tract
alphaFWE: 0.000314
min p-value: 0.052499
t-value: -1.962847
CG_left
alphaFWE: 0.000314
min p-value: 0.02365
t-value: 2.298633
CG_right
alphaFWE: 0.000314
min p-value: 0.071717
t-value: 1.820617
CST_left
alphaFWE: 0.000234
min p-value: 0.00023
t-value: 3.865593
CST_right
alphaFWE: 0.000236
min p-value: 0.000471
t-value: 3.686151
FPT_left
alphaFWE: 0.000258
min p-value: 0.017802
t-value: -2.410772
FPT_right
alphaFWE: 0.000255
min p-value: 0.000742
t-value: -3.485555
ICP_left
alphaFWE: 0.000249
min p-value: 0.000619
t-value: -3.635711
ICP_right
alphaFWE: 0.000289
min p-value: 0.00135
t-value: -3.377354
IFO_left
alphaFWE: 0.000314
min p-value: 0.1062
t-value: -1.630529

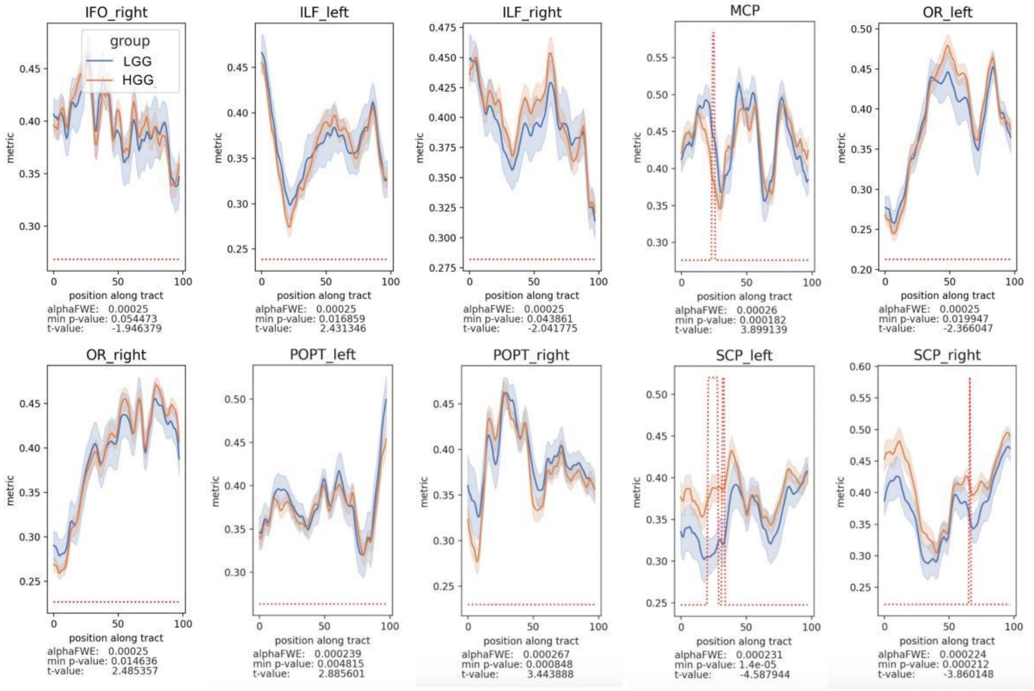
IFO_right
group
LGG
HGG
metric
position along tract
alphaFWE: 0.00025
min p-value: 0.054473
t-value: -1.946379
ILF_left
alphaFWE: 0.00025
min p-value: 0.016859
t-value: 2.431346
ILF_right
alphaFWE: 0.00025
min p-value: 0.043861
t-value: -2.041775
MCP
alphaFWE: 0.00026
min p-value: 0.000182
t-value: 3.899139
OR_left
alphaFWE: 0.00025
min p-value: 0.019947
t-value: -2.366047
OR_right
alphaFWE: 0.00025
min p-value: 0.014636
t-value: 2.485357
POPT_left
alphaFWE: 0.000239
min p-value: 0.004815
t-value: 2.885601
POPT_right
alphaFWE: 0.000267
min p-value: 0.000848
t-value: 3.443888
SCP_left
alphaFWE: 0.000231
min p-value: 1.4e-05
t-value: -4.587944
SCP_right
alphaFWE: 0.000224
min p-value: 0.000212
t-value: -3.860148

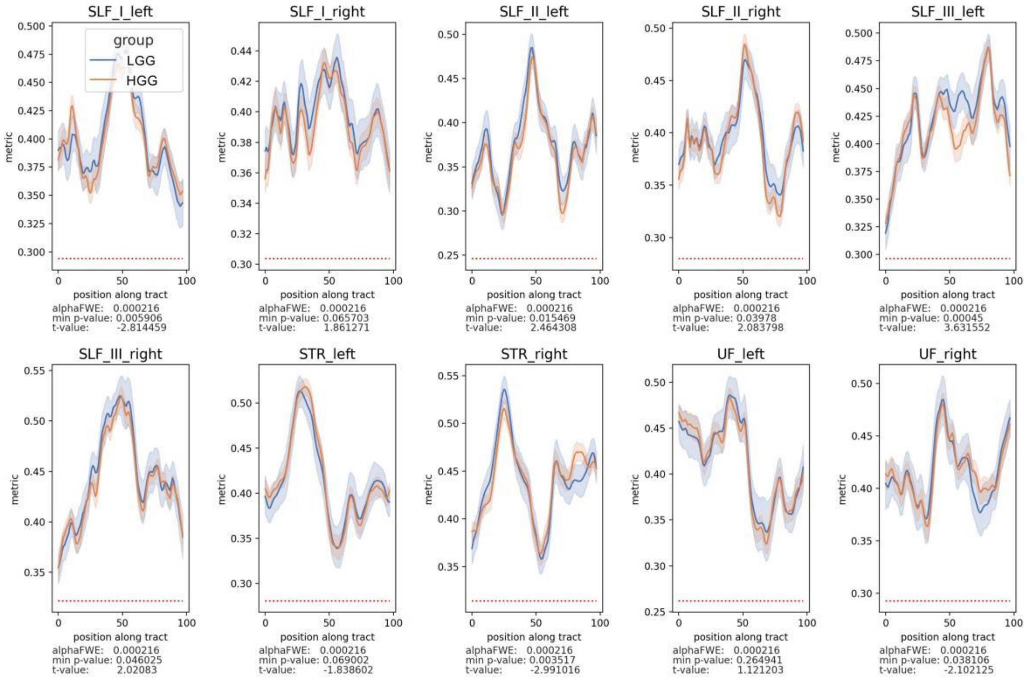
SLF_I_left
group
LGG
HGG
metric
position along tract
alphaFWE: 0.000216
min p-value: 0.005906
t-value: -2.814459
SLF_I_right
alphaFWE: 0.000216
min p-value: 0.065703
t-value: 1.861271
SLF_II_left
alphaFWE: 0.000216
min p-value: 0.015469
t-value: 2.464308
SLF_II_right
alphaFWE: 0.000216
min p-value: 0.03978
t-value: 2.083798
SLF_III_left
alphaFWE: 0.000216
min p-value: 0.00045
t-value: 3.631552
SLF_III_right
alphaFWE: 0.000216
min p-value: 0.046025
t-value: 2.02083
STR_left
alphaFWE: 0.000216
min p-value: 0.069002
t-value: -1.838602
STR_right
alphaFWE: 0.000216
min p-value: 0.003517
t-value: -2.991016
UF_left
alphaFWE: 0.000216
min p-value: 0.264941
t-value: 1.121203
UF_right
alphaFWE: 0.000216
min p-value: 0.038106
t-value: -2.102125

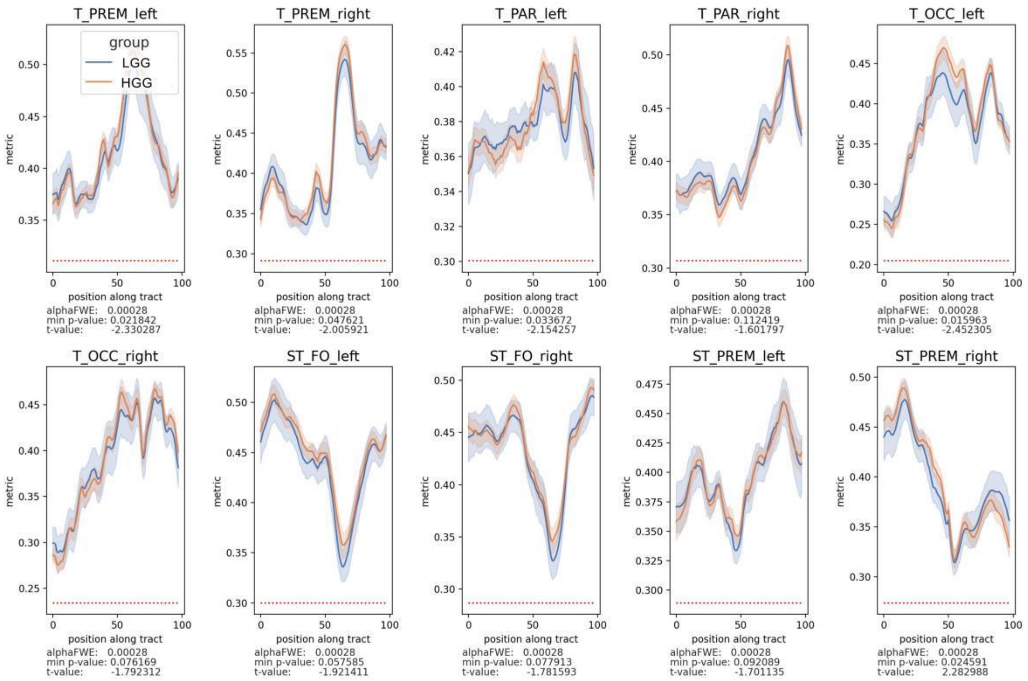

Supplementary Figure 16. Quantitative analysis of Fak values of fifty white matter tracts in patients with LGG versus patients with HGG.
